# Unraveling the Concept of Childhood Adversity in Psychosis Research: A Systematic Review

**DOI:** 10.1093/schbul/sbae085

**Published:** 2024-05-30

**Authors:** Sjur S Sætren, Jone R Bjørnestad, Akiah A Ottesen, Helen L Fisher, Daniel A S Olsen, Kari Hølland, Wenche ten Velden Hegelstad

**Affiliations:** Department for Child and Adolescent Research, Norwegian Centre for Violence and Traumatic Stress Studies, Oslo, Norway; TIPS Centre for Clinical Research in Psychosis, Stavanger University Hospital, Stavanger, Norway; TIPS Centre for Clinical Research in Psychosis, Stavanger University Hospital, Stavanger, Norway; Institute of Social Studies, Faculty of Social Sciences, University of Stavanger, Stavanger, Norway; Department of Psychiatry, District General Hospital of Førde, Førde, Norway; NORMENT, Division of Mental Health and Addiction, Oslo University Hospital & Institute of Clinical Medicine, University of Oslo, Oslo, Norway; Social, Genetic & Developmental Psychiatry Centre, Institute of Psychiatry, Psychology & Neuroscience, King’s College London, London, UK; ESRC Centre for Society and Mental Health, King’s College London, London, UK; TIPS Centre for Clinical Research in Psychosis, Stavanger University Hospital, Stavanger, Norway; Institute of Social Studies, Faculty of Social Sciences, University of Stavanger, Stavanger, Norway; TIPS Centre for Clinical Research in Psychosis, Stavanger University Hospital, Stavanger, Norway; Institute of Social Studies, Faculty of Social Sciences, University of Stavanger, Stavanger, Norway

**Keywords:** psychosis, childhood adversity, childhood trauma, child maltreatment, measurement, assessment

## Abstract

**Background:**

During the last decades, an abundance of studies has investigated childhood adversity in relation to psychosis. This systematic review critically examines the methodologies employed to investigate childhood adversity in psychosis over the past decade, including operational definitions, measurement tools and characteristics, and psychometric properties of instruments used in these studies.

**Study Design:**

This systematic review followed the PRISMA guidelines (registration number CRD42022307096), and the search used the following electronic databases: PsychINFO, SCOPUS, Web of Science, African Index Medicus (AIM), LILACS, CINAHL, EMBASE, and MEDLINE. The search included variations and combinations of the terms targeting childhood adversity and psychosis.

**Study Results:**

Out of 585 identified studies published between 2010 and 2023, 341 employed a validated instrument to investigate childhood adversity. Our findings show “childhood trauma” being the most frequently examined construct, followed by “child maltreatment” or “child abuse.” The short version of the Childhood Trauma Questionnaire was the dominant instrument. Physical abuse, emotional abuse, and sexual abuse were most frequently investigated, and indeed the field appears generally to focus on child abuse and neglect over other adversities. Significant psychometric heterogeneity was observed in the selection and summarization of instrument items, with only 59% of studies documenting original psychometric validation and 22% reporting reliability in their datasets.

**Conclusion:**

This review highlights substantial methodological heterogeneity in the field, pointing out biases in the research on childhood adversity and psychosis. These findings underline the need for standardized definitions and high-quality measurement tools to enhance the validity of future research in this area.

## Introduction

Over the past several decades, extensive research has explored the link between childhood adversity and psychosis.^1–3^ However, childhood adversity often used synonymously with constructs such as childhood trauma, describes exposure to a wide range of detrimental childhood environments. These can include abuse, neglect, social deprivation, war, natural disasters, peer sexual abuse, bullying, life-threatening accidents, witnessing death or violence, and more. Childhood adversity as a multifaceted construct has been extensively investigated as a risk factor for various negative outcomes,^4^ including psychosis.^1,5,6^ However, problems of interpreting and comparing existing findings have been ascribed to conceptual and methodological differences across studies and a lack of consensus about defining, assessing, and operationalizing childhood adversity.^7–9^ This is common to most research fields, and psychosis research is no exception.^10^ The lack of uniformity threatens the comparability of existing evidence and can lead to biases in future studies.

The literature reports different approaches to studying the association between childhood adversity and psychosis. Several studies report on the prevalence of “adverse childhood events,” defined in numerous ways.^11^ Others have investigated how childhood adversity contributes to the onset and course of psychosis,^1^ among which the dose-response relationship of type of adversities on psychosis is the most well-known.^1,12^ For example, increased exposure to emotional abuse significantly elevates the likelihood of experiencing psychosis symptoms.^12^ The most conventional approach has been to separate and compare the effects of specific types of adversities on psychosis.^1^ Yet, because of the overlap and co-occurrence of different types of exposures,^13,14^ there has been a growing emphasis on understanding how other dimensions of adversity, including developmental timing, duration, and social context, may relate to psychosis.^15^ Finally, some studies have treated those with and without histories of adversity as specific phenotypes within psychosis.^16^

There is also a lack of agreement on defining, assessing, and operationalizing childhood adversity.^7–9^ First, investigators have used a variety of terms to describe and differentiate exposure to adverse childhood events.^17,18^ Terms such as childhood adversity, childhood trauma, victimization, and child maltreatment or abuse are used synonymously or interchangeably without any clear operational definition.^18^ Second, the heterogeneity may reflect disagreement about what should be assessed. Measures of childhood adversity vary greatly regarding the dimensions of childhood adversity being measured, in terms of *types* (eg, physical abuse, emotional abuse, sexual abuse, and neglect) and other *characteristics* of childhood adversities (eg, severity, frequency, timing, and duration). Categorizing childhood adversity into different types of adversities, such as physical, psychological, or sexual abuse, has resulted in several methodological limitations in investigating the impact on mental health.^17^ Studies cannot rule out that such categories include overlapping constructs. In line with this, evidence of specific effects for neurobiological or psychological responses to one type of exposure over another thus remains unclear.^17^ It may be that different types of childhood adversity equally contribute to the risk for psychopathology,^4^ while specific characteristics such as timing, duration, social context, or relationship to a perpetrator could have a particular influence on development.^19^ Third, there is a lack of psychometric accountability.^2,20^ Most studies have been criticized for using assessments of low quality and for heterogeneity of measures.^2,20^ A recent systematic review of the psychometric quality of instruments used in psychosis research found psychometric validity and reliability to be lacking overall.^20^ Variations in assessment format, such as self-report vs interview or the number of items used, may further bias findings and explain diverging results.^9^ This methodological heterogeneity may have biased the existing evidence on childhood adversity and psychosis in several ways. Therefore, it may be useful to clarify these concepts and assessments of childhood adversity in psychosis research.

The aim of this review was to explore and clarify how childhood adversity has been operationalized and assessed in psychosis research along the spectrum from psychotic-like experiences to psychotic disorders. More specifically, in individuals at risk for psychosis (P), how is childhood adversity operationalized and measured (E) in relationship with the development of and course in psychosis (O).^21^ Following this, we defined 4 research questions: **(1)** what constructs (eg, childhood adversity, childhood trauma, child abuse, etc.) are measured; **(2)** what instruments are used; **(3)** what types and characteristics of childhood adversity are measured; **(4)** if and how psychometric characteristics of instruments are reported within studies exploring associations between childhood adversity and psychosis.

## Method

### Operationalization of Terms

#### Childhood Adversity

Consistent with recommendations for future directions in childhood adversity research,^18^ we adopted the following definition of childhood adversity: “Isolated, repeated, or chronic exposure during childhood or adolescence to environmental circumstances necessitating significant psychological, social, or neurobiological adaptation by an average child and representing a deviation from the expectable environment” (p. 6). This encompasses exposures like maltreatment, abuse, neglect, domestic violence, assault, bullying, discrimination, violence, victimization, parental loss, and bereavement. We employed this broad definition to ensure the inclusion of a wide range of studies.

#### Psychosis

We refer to psychosis as a continuum along the psychosis spectrum,^22^ which includes mental health conditions characterized by disruptions in thought and perception, spanning full psychotic disorders like schizophrenia to psychosis-like symptoms.^23^ Symptoms, varying in type and intensity, include hallucinations, delusions, and disorganized thinking, influencing both diagnosis and treatment.

### Search Strategy

This systematic review followed the PRISMA guidelines,^24^ and the protocol was registered in the International Register of Systematic Reviews (PROSPERO) in March 2022 (registration number CRD42022307096). The initial search was conducted in March 2022 and an updated search was performed in October 2023. The search used the following electronic databases: PsychINFO, SCOPUS, Web of Science, African Index Medicus (AIM), LILACS, CINAHL, EMBASE, and MEDLINE. Variations and combinations of the terms targeting childhood adversity AND psychosis were included in the search. A complete overview of the search strategy is presented in supplementary table S1.

### Eligibility Criteria

We searched for studies investigating the relationship between childhood adversity and psychosis from the past decade (until the date of our initial systematic search) to ensure that our systematic review reflects the most current methodologies on childhood adversity assessment in psychosis research. For inclusion, articles had to meet the following criteria: (1) empirical study published in English in a peer-reviewed journal between 01.01.10 and 19.10.23; (2) any exposure defined under the domains of childhood adversity (details provided in supplementary table S1) that occurred prior to the age of 18, as the predictor/independent or mediator/moderator variable; (3) psychotic disorder, schizotypal traits, psychotic symptoms, psychotic/psychosis-like experience (PLEs), or clinical- or ultra-high-risk as the outcome/dependent variable. Also eligible were studies investigating associations between childhood adversity and psychosis within the same sample, and prevalence studies of childhood adversity within a sample of individuals with psychotic/psychosis-like symptoms. Articles were excluded if: (1) they had a case report or review design; (2) the timing of adversity was not specified (i.e., over/under the age of 18); (3) samples with organic psychosis with no separate data provided for non-organic psychosis. For the final analysis, studies using a validated instrument were selected by examining references to their validation studies. In cases where these references were not explicit, we reviewed the original sources in accordance with established guidelines for psychometric validity.^25^

### Screening

All potential studies found through the initial search were exported into Endnote,^26^ which was used to remove duplicates. Articles were exported into Rayyan,^27^ a web app for organizing systematic reviews. In the second step, 4 authors independently screened titles and abstracts for initial inclusion (S.S.S. all articles, J.B., W.T.V., and A.A.O. 1/3 each). The authors reached interrater agreement through several consensus meetings. In the case of disagreements, full-text papers were read and discussed between S.S.S. and J.B., W.T.V., and A.A.O. to achieve consensus. In the third step, the same 4 authors independently assessed articles at full-text level (S.S.S. all articles, J.B., W.T.V., and A.A.O. 1/3 each). The authors discussed disagreements to achieve consensus through 2 consensus meetings. An updated systematic search and review following the same steps was conducted (by S.S.S. and D.S.O.).

### Data Extraction and Analysis

Authors S.S.S. and D.S.O. performed a narrative synthesis to map and organize operationalizations of childhood adversity. They summarized the findings to answer the research questions and to interpret results^28^ by producing descriptive paragraphs covering the name, concept of interest, and measure. They also recorded how many instruments were used and if non-validated extra questions had been included. A modified template to describe the way in which child abuse and neglect are measured in prevalence studies^7^ was applied to tabulate operationalizations across studies. This included recording the concept being measured, format (self-report or semi-structured interview), type of adversity, characteristics of adversity (social context, relation to offender, timing, duration, frequency, threat vs. deprivation), and outcome. They also recorded documentation of psychometric data (reliability and validity). All items were dichotomized into 0 or 1. Descriptive tables and graphs were then generated for each category, summarizing the findings according to each research question.

The next step consisted of systematically organizing the studies into distinct clusters or themes guided by the research questions. Data synthesis of frequencies, tables, and figures was conducted in Excel, version 2018,^29^ and SPSS version 26.^30^ To address our first research question, we categorized the studies based on the specific construct of interest being investigated. For our second research question, we focused on identifying the instruments utilized to measure the construct under investigation. Studies employing non-validated instruments, including clinical diagnostic interviews or registry data, were labeled and excluded from the final analysis. For our third research question, we organized the studies based on the specific types and characteristics of childhood adversity being measured. Answering our last research question involved documenting whether studies reported the psychometric quality of the measures. Furthermore, we present an overview of instruments used to measure childhood adversity across studies, as well as information pertaining to their validity in patients with psychosis. This was based on a recent meta-analytic review.^20^

## Results

**Fig. 1.**
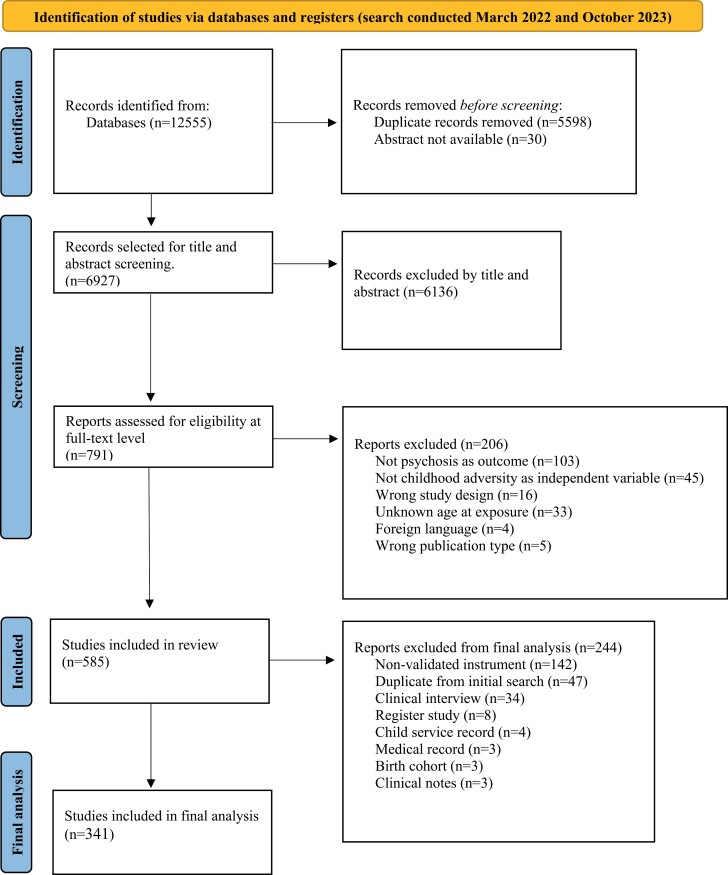
PRISMA flowchart of literature screening of papers examining the association between childhood adversity and psychosis.

The systematic search across databases identified 12 555 records, which after the removal of duplicates resulted in 6927 articles for further screening. Subsequently, 6136 articles were excluded through title and abstract screening, resulting in 791 articles for full-text screening (for an overview of excluded studies with reasons, see supplementary table S4). In line with our eligibility criteria, 585 articles were included in the final review. This revealed that 163 studies did not use a validated instrument, 34 used clinical interviews, 8 used register data, and 6 used child-service records. We identified 341 studies using a validated instrument, which were further analyzed in line with our research questions. See figure 1 for a summary of the literature screening process.

### Construct of Interest

**Fig. 2.**
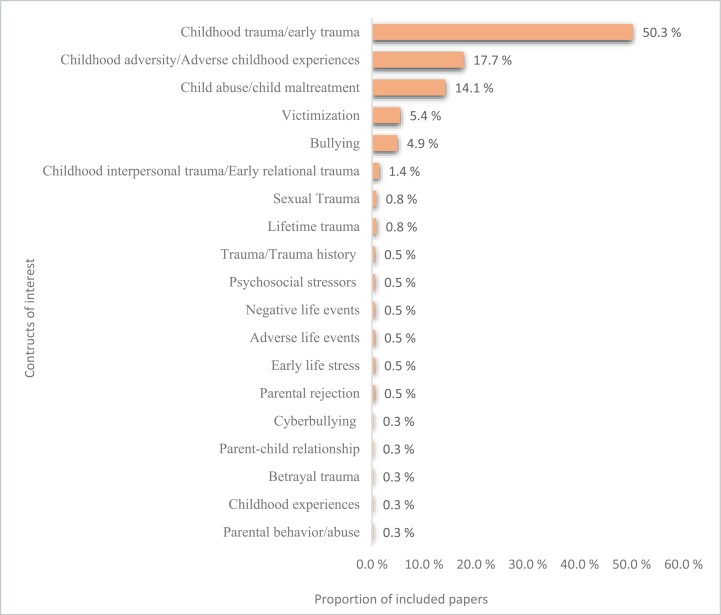
Percentage of constructs utilized to define childhood adversity across included studies.

We identified 21 different constructs. “Childhood trauma” or “early trauma” were used most frequently (50%), followed by “childhood adversity” or “adverse childhood events” (18%) and “childhood abuse/child maltreatment” (14%). For a complete overview see figure 2.

### Instruments, Format, Number of Items, and Psychometric Properties

**Fig. 3.**
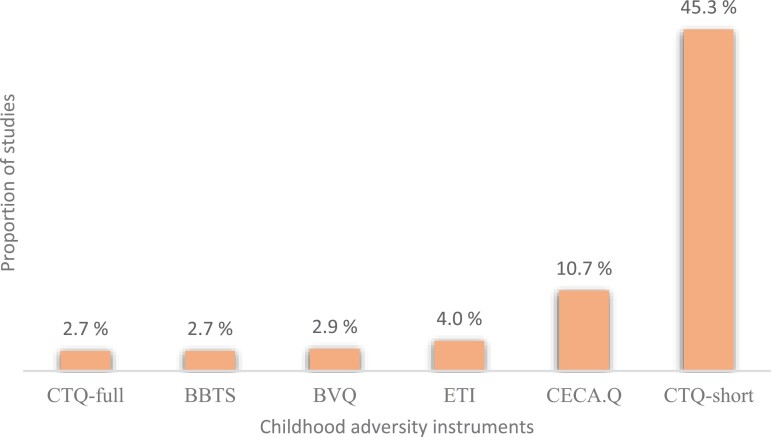
Top 6 instruments used to measure childhood adversity across studies. *Note*: BBTS, Brief Betrayal Trauma Survey; BVQ, Bully/Victim Questionnaire; CECA.Q, Childhood Experience of Care and Abuse Questionnaire; CTQ-full, Childhood Trauma Questionnaire full version; CTQ-short, Childhood Trauma Questionnaire short version; ETI, Early Trauma Inventory.

We identified 49 different instruments in studies using validated assessments. For a complete overview including measure, type of adversity, administration method, response format, number of items, and validation for psychosis, see supplementary table S5. Eighty-six percent of studies used self-report measures, 12% used semi-structured interviews, and 2% a combination of both. The Childhood Trauma Questionnaire-short (CTQ-short)^31^ was the most widely used instrument, followed by the Childhood Experience of Care and Abuse Questionnaire^32^ (CECA.Q) and the Early Trauma Inventory^33^ (ETI; see figure 3). Different instruments varied considerably in the number of items they included, ranging from 6 to 75 (see also supplementary table S2). About 30% of the studies did not report the number of items used, 8% used more than 1 instrument, while 13% reported that they had used non-validated extra questions or only a restricted number of items from an instrument.

In terms of response summarization, sum scores (31%) were most frequently used, followed by predefined cutoff scores (20%), yes/no (10%), and mean scores (5%). While 59% (*n* = 203) of the studies referenced the original psychometric validation for their chosen instrument, a mere 22% (*n* = 76) reported reliability based on their own dataset. Consequently, approximately 40% of the studies neither documented nor referred to any psychometric properties of the instruments they utilized.

### Types of Childhood Adversity

**Fig. 4.**
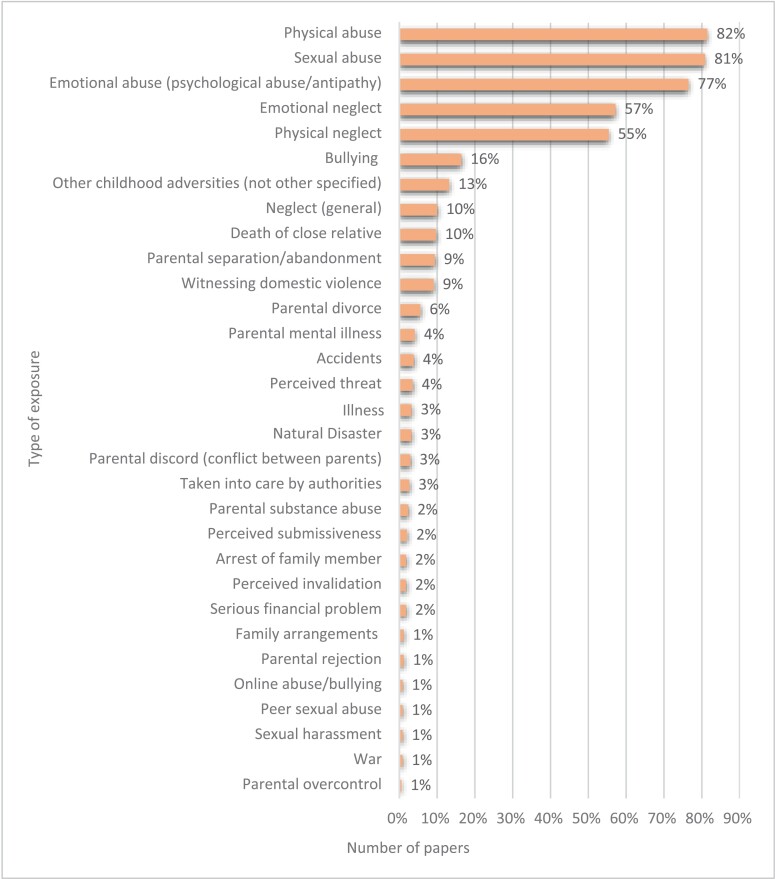
Prevalence of the type of adversities being measured across studies.

Type of adversity was reported in all the included studies using a validated instrument. Figure 4 presents an overview of the types of childhood adversities that were measured. Physical (82%; *n* = 278) and sexual abuse (81%; *n* = 276) were most often investigated, followed by emotional abuse (77%; *n* = 261), emotional neglect (57%; *n* = 195), and physical neglect (55%; *n* = 189). Most studies on abuse and neglect did not investigate other adversities.

Other adversities, such as the death of a close relative (10%; *n* = 33), parental separation/abandonment (9%; *n* = 32), witnessing domestic violence (9%; *n* = 31), parental divorce (6%; *n* = 19), and parental mental illness (4%; *n* = 14), were rarely investigated. We also observed this for being placed away from the birth family by child protection services (*n* = 8) and for other changes in family arrangements (1%; *n* = 4). Adversities such as war (1%; *n* = 3), natural disasters (3%; *n* = 11), accidents (4%; *n* = 13), or family financial problems (2%; *n* = 6) were rarely investigated.

Bullying was also investigated relatively infrequently (*n* = 47), often studied in isolation, and not taking into account physical (57%) and sexual (55%) abuse, and even less often physical (31%) and emotional (42%) neglect. Very few studies investigated online bullying (1%; *n* = 3), sexual abuse by peers (1%; *n* = 3), or sexual harassment (1%; *n* = 3).

### Other Characteristics of Childhood Adversity

Other characteristics of childhood adversity were scarcely documented. Severity (13%; *n* = 56) and frequency (11%; *n* = 47) are most often reported, followed by timing of exposure (5%; *n* = 23), multiform exposure/cumulative exposure (3%; *n* = 12), and duration of exposure (3%; *n* = 12). We did not identify any studies investigating threat or deprivation as underlying dimensions of childhood adversity exposure.^19^ Very few of the studies specified a relationship to the perpetrator (8%; *n* = 29), where 5% specified a mother (*n* = 16) and 5% a father (*n* = 15).

## Discussion

This systematic review explored 585 studies published between 2010 and 2023 examining associations between childhood adversity and psychosis ranging from subclinical psychotic-like manifestations to clinically diagnosed psychotic disorders. The primary objective was to explore how childhood adversity is operationalized in psychosis research. In pursuit of a comprehensive understanding of the existing evidence, this review refrained from imposing any restrictions regarding methodological quality, except for the requirement that a validated self-report instrument or semi-structured interview had been used.

### Ambiguity in constructs (1)

Studies conceptualize and assess childhood adversity in a wide range of ways. When this is not explicitly specified, it hampers the reliability and validity of conclusions regarding associations between childhood adversity and psychosis. We identified 18 different childhood adversity constructs, often used interchangeably to describe the same type or related type of exposure (eg, physical abuse, sexual abuse, etc.). “Childhood trauma” for instance, was very often used synonymously with “child maltreatment/child abuse,” even when they were measured using the same tool. Using terms such as “childhood trauma” and “child maltreatment/child abuse” interchangeably can lead to a misunderstanding of concepts, as they are overlapping but not equal. For instance, all forms of child maltreatment, including abuse and neglect, are potentially traumatic, but not all childhood trauma results from child maltreatment.^18^

Furthermore, in research on bullying and psychosis, the terms “bullying” and “victimization” were concurrently used in 8 studies. In certain instances, “victimization” was used as a standalone term to denote bullying or peer victimization.^34^ Yet, the term “victimization” was also applied in broader contexts, referring to experiences of “childhood trauma” or “child abuse,” potentially leading to ambiguities in interpretation.^35,36^

### Selection of instruments (2)

Our review highlights the predominant use of the CTQ-short^31^ across studies. This offers methodological advantages such as consistent data collection, enhanced comparability of results, and suitability for meta-analyses. CTQ-short is one of the few instruments validated for research in psychosis.^20^ This increases the quality of the data and ensures construct validity and reliability. Thus, this instrument can produce consistent results and accurately measure what it intends to measure. However, the CTQ-short^31^ focuses primarily on child abuse and neglect (ie, physical abuse, emotional abuse, sexual abuse, emotional and physical neglect), which undeniably introduces a methodological limitation when investigating a broader spectrum of childhood adversities. When the current evidence on childhood adversity and psychosis is primarily founded on CTQ-short,^31^ we cannot know how other childhood adversities beyond abuse and neglect might play a role in psychosis. By focusing predominantly on certain childhood adversities, we may create a narrative about childhood adversity and psychosis that could be biased or incomplete. The narrow range of adversities that are included in instruments such as CTQ-short^31^ or CECA.Q,^37^ also reduces the possibility to compare exposure to other prevalent adversities in the psychosis population, such as bullying.^38^ In addition, instruments such as CTQ do not include other dimensions of childhood adversity, such as timing of exposure and duration of exposure.

### Type and characteristics of childhood adversities (3)

As highlighted by our results, “childhood adversity” is a multifaceted construct in psychosis research, mainly investigated by measuring types of exposure. This confirms findings from a previous methodological review,^39^ in that subtypes of adversities have been the most commonly studied dimensions of childhood adversity. Despite the wide range of exposures identified in our review, it is noted that certain types of exposures are disproportionately represented. In the context of psychosis, the most thoroughly investigated exposures are physical and sexual abuse, followed by emotional abuse, physical neglect, and emotional neglect. The explanation of the main focus on abuse and neglect exposure within the literature is the dominant use of the CTQ-short,^31^ which only includes these types of exposures.

This dominant emphasis on a restricted range of adversities represents a methodological issue for various reasons. First, it leads to an incomplete understanding of the true range and complexity of childhood adversity in relationship to psychosis. If the evidence is heavily weighted toward specific exposures such as physical or sexual abuse, other forms of adversity (eg, bullying, witnessing domestic violence, war, gang violence, accidents, natural catastrophes) might be undervalued or overlooked. This could limit our comprehension of whether and how these less-studied forms of adversity contribute to the development of psychosis. Second, this unbalanced focus leads to skewed results in studies of childhood adversity. Although certain types of adversity could have a unique impact on psychosis, exposure to multiple types occurs more commonly than being exposed to single events.^4^ Given the potential inter-relatedness and/or overlap of different forms of adversity,^17^ studying certain exposures in isolation may result in a misinterpretation of their true effects. Further, there is a lack of consistent evidence for specific effects of specific types of adversities.^17^ The literature reports differing findings, for instance, that physical or sexual abuse is associated with overall symptoms^40^ but also with aggressive behavior,^41^ whereas this type of adversity was not associated with general functioning.^42^ Regarding neglect, some findings indicate an association with negative and depressive symptoms but not with manic or positive symptoms,^40^ while others indicate associations with general functioning.^42^ Studies that adjust for co-occurrence tend to find that different types of adversities have equal effects on psychiatric and behavioral outcomes.^4^ For instance, a child who experiences bullying might also be living in a home with parental substance abuse, and these combined adversities could have a multiplying or interactive effect on his or her mental health outcomes. Additionally, our findings indicate that other types of exposures, including parental separation or loss, problematic family dynamics or arrangements, parental divorce, or having a parent with mental illness or substance abuse, are considerably less studied within the existing body of research. Finally, the categorization of adversities is frequently ambiguous, with the type of adversity and number of items showing variability across different studies and instruments used to measure specific exposures.^17^

Other dimensions beyond the mere presence or absence of types, such as timing, frequency duration/chronicity, or the context in which the adversity occurs, might provide distinct perspectives on the evolution of psychopathology.^17,19^ However, as our review highlights, the existing evidence is biased toward the study of types and lacks studies investigating specifically whether and the degree to which timing, duration, or multi-exposure is associated with psychosis.

Finally, some notes on the term “childhood trauma.” This term does not distinguish between the event(s) or adversities on the one hand, and the psychological reactions to it on the other hand. There is little to suggest that all childhood adversities also confer potential psychological injury or damage. Thus, while childhood adversity and childhood trauma often are used synonymously, some important distinctions should be clarified. The contemporary definition of trauma, as outlined in the Diagnostic and Statistical Manual of Mental Disorders, Fifth Edition (DSM-5)^43^ or the International Classification of Diseases and Related Health Problems (ICD-11^44^), builds on the definition of exposure to actual or threatened death, severe injury, or sexual violence, either directly or indirectly through witnessing such events or being exposed to them in close relatives or friends. Consequently, exposure to childhood adversity may encompass, but is not limited to, exposure to childhood trauma in this strict sense of the term. For example, exposures to emotional or physical neglect or repeated parental separations or rejections represent childhood adversities but need not be traumatic according to this definition. Indeed, “trauma” implies potential traumatic reactions to adverse events, also reflected by diagnostic criteria for post-traumatic stress disorder, whereas childhood adversity describes events or exposures that deviate significantly from expected environments without necessarily fulfilling the criteria of being traumatic.^18^ To sum up, the concept of childhood trauma refers to events eliciting post-traumatic reactions, while childhood adversity describes exposures that significantly deviate from the expected environments. Specifying focus on either the events, the psychological consequences, or both can help bring clarity to this field of research.

### Psychometric heterogeneity (4)

The vast majority (86%) of studies used self-report formats. While self-report questionnaires are advantageous due to easy administration and the ability to gather data from large cohorts rapidly, responses may be biased by individual factors such as cognitive impairment or psychotic symptoms.^45^ On the other hand, although these concerns should be explored further in psychosis populations, evidence suggests a fair agreement between self-report and interview format,^46^ and thus that self-report measures may be reasonably valid for assessing childhood adversity in samples with psychosis. However, semi-structured interviews can more comprehensively capture contextual details of childhood adversity and identify potential reactions to adverse events that are difficult to ascertain with self-report instruments.^46^ Nonetheless, interviews also have drawbacks: they are time-consuming, and reliable execution usually demands extensive interviewer training. In many cases, a more cost-effective means of collecting data will be needed. In these cases, self-report questionnaires serve as viable alternatives.

Our review indicated a broad variability in the number of items used from each instrument. In addition, nearly one-third of the studies did not specify how many items they used. This may imply the use of the full scale, but the lack of transparency challenges the reproducibility and comparability of studies. The use of multiple instruments by 8% of studies may indicate an attempt to achieve a more comprehensive assessment of a diverse spectrum of adversities. Still, studies adding non-validated questions or cherry-picking items from validated tools raise concerns about consistency and validity.

Sum scores in several studies served as a cumulative metric for adversity and typically indicated frequency or intensity. Conversely, predefined cutoff scores produced dichotomous yes/no values and straightforward binary categorizations. Mean scores in turn captured an average magnitude. This range of approaches to establish childhood adversity reflects the diverse research objectives in studies examining associations between childhood adversity and psychosis and a lack of methodological cohesion. Our findings resonate with prior systematic reviews and meta-analyses,^2,3^ underscoring the difficulty in comparing results across studies.

A methodological concern emerges from the observation that approximately a quarter of the identified studies used non-validated procedures to assess childhood adversity. For the studies using a validated instrument, 59% referred to the original psychometric validations, and only a modest 22% conducted reliability assessments with their own datasets. Remarkably, about 40% of the studies did not mention psychometric properties, casting doubts on the validity of their results. This point resonates with findings from another systematic review on childhood adversity and psychosis, highlighting the imperative for stringent validation of instruments in psychosis research.^20^ This challenge predominantly stems from the varied methodological approaches adopted in measuring childhood adversity.

### Recommendations for Future Research

1. Defining childhood adversity

The construct of childhood adversity still lacks a definitive, universally accepted definition, and remains an ongoing area of exploration. To increase the consistency and validity of research outcomes, it is fundamental to first explicitly define the construct being examined. A precise definition provides a scaffold for the entire investigation, ensuring that all subsequent stages of research align with the initial intent. We believe that establishing standardized terminology would be helpful for greater clarity in the field. A consistent and universally accepted set of definitions would enhance the comparability across various studies, strengthening the reliability and validity of the subsequent findings and conclusions. Furthermore, we argue that studies should explicitly delineate the types of adversities they encompass when utilizing broad constructs like “childhood adversity” or “childhood trauma.” Such transparency will bolster the potential for comparable results across different research endeavors. Several attempts have been made to build a scientific consensus.^18^ Following suggestions from these attempts, childhood adversity is considered to include several components:

*Deviation from an expected environment characterized by threat or deprivation*: An expected environment refers to environmental inputs required to develop normally, and includes sensory inputs, exposure to language and communicative stimuli, and sensitive responsive caregiver relationships.^47^ An unexpected environment can be separated along 2 dimensions: deprivation of expected inputs or basic needs (eg, neglect from emotional support), or the presence of unexpected inputs presenting a threat to the individual (eg, physical, emotional and sexual abuse, or life-threatening accidents^18^).

*Requires significant psychological, social, or neurobiological adaptation*: Childhood adversity includes exposures that demand significant adaptation from an individual, thereby excluding events that represent typical, normative stressors or minor challenges. To elucidate this distinction, researchers have been encouraged to explicitly clarify the differentiation between adversity and normative stressors in their studies,^18^ as this may vary across cultures and countries.

*Isolated, repeated, or chronic exposure*: Childhood adversity encompasses a spectrum of experiences, ranging from isolated incidents to recurring or chronic exposure to adverse conditions. These experiences can lead to a deviation from the expected normative environment.

*During childhood or adolescence*: The most definitive criteria for childhood adversity pertain to the timing of the exposure, specifically occurring during childhood or adolescence before the age of 18. In addition, childhood adversity can also be specified according to developmental periods.

1. Expanding the framework of childhood adversity in psychosis research

Childhood adversity is a multifaceted and multidimensional construct and there is an ongoing debate on how childhood adversity best can be conceptualized.^17,48^ While child abuse and neglect are important domains of childhood adversity, the present review illustrates that there are many adversities being less investigated. For instance, in an increasingly globalized world, many children migrate under severely stressful circumstances such as war, natural disasters, human trafficking, and others, and the associations to mental health generally and psychosis specifically, in our view deserve more study. Furthermore, other dimensions of childhood adversity apart from type concern timing, duration, and the context and relationship within which the exposure occurs. These have also been surprisingly under-investigated in psychosis research. Recent models of childhood adversity suggest also to investigate the underlying mechanisms of adversity exposure along dimensions of threat and deprivation.^19^ Future investigations are warranted to explore the application of this approach within the domain of psychosis research, as our review did not reveal any studies currently employing this methodology.

In addition, traditional methods of assessing childhood adversity fail to capture the unique challenges and stressors that children face in an increasingly connected and digitalized environment. For instance, cyber-bullying and exposure to online abuse are contemporary issues that can significantly impact a child's mental health and development.^49^ Furthermore, the measurement instruments need to be sensitive to cultural differences in the perception and impact of adversity. The integration of digital exposure along with a more culturally inclusive framework, is essential to provide a comprehensive and accurate understanding of the impact of childhood adversity and risk for psychosis. Recent research also acknowledges the importance of measuring psychosis-related adversities.^50^

1. Co-occurrence of adversity exposures

Exposure to adversity rarely occurs in isolation.^13,14^ The high co-occurrence between adversities presents several statistical problems (eg, multicollinearity) when calculating effect sizes for specific types of adversities in relationship with psychosis. Models designed to predict specific effects of adversities on risk for psychosis should therefore apply strategies to account for such co-occurrences.^48^ Ignoring these overlaps might lead to skewed interpretations, possibly overestimating or underestimating the true impact of an individual exposure.

1. Psychometric evaluation for psychosis populations

The tools and instruments used in research should not just be accurate but also appropriate for the population in question. In general, there is a lack of good quality evidence regarding the psychometric quality of childhood adversity measures for the psychosis population. A recent review has addressed the psychometric quality of adversity measures for use with psychosis populations.^20^ Only 24 studies evaluating 17 instruments were identified, of which 8 measured exposure to childhood adversity.^20^ The Childhood Trauma Questionnaire (CTQ) was shown to be the best-performing instrument, and the only instrument rated higher than “low quality” on multiple psychometric properties. Reviews have put forth recommendations for specific measures.^51^ In the context of psychosis, future research is encouraged to incorporate these recommendations in the selection of methods for assessing childhood adversity. Building upon these insights in subsequent studies will contribute to a better understanding of the relevance of childhood adversity in relation to psychosis.

### Limitations

Although our comprehensive systematic review exhibits several significant strengths, it is not without methodological limitations. Primarily, our search strategy was restricted to automated tools within electronic databases, excluding manual literature searches. This methodology, while extensive and efficient, could potentially overlook studies not indexed in the main databases or those situated within the gray literature, thereby possibly limiting the scope of our review. Furthermore, our study did not undertake an assessment of the evidence quality. The scope of this systematic review was the documentation of the quality of childhood adversity assessments within psychosis research, congruent with our paper’s key objectives. Consequently, broader appraisals of study quality, including evaluations of bias risk or the aggregate strength of the evidence base, were not performed.

While our comprehensive exploration of the literature is a significant strength, our decision to perform a narrative synthesis approach may have come at the cost of a more detailed examination of the psychometric properties of the included measures. Other systematic review methodologies might provide a more exhaustive insight, but we argue that this is being covered by recent meta-analytic studies.^20^

We did not focus on the details of item formulation or analyze the number of items corresponding to each type of adversity. This may have limited our view of certain nuances regarding different operationalizations. The review did not distinguish between psychotic disorders and psychosis-like symptoms. Consequently, potential differences in childhood adversity measures relating to clinical vs nonclinical populations might have gone undetected in our review. While we aimed to cover an extensive range of childhood adversities, the possibility remains that not all relevant exposures were captured. Our investigation did not encompass types of neglect beyond emotional and physical neglect. Other forms of neglect, such as educational or medical neglect, that might have a bearing on the relationship with psychosis were not explored. Our review did not delve into specific sub-constructs of bullying or peer victimization beyond online abuse and peer sexual abuse. Distinctions such as between verbal, physical, and indirect bullying were not made, potentially missing specific nuances in how these different forms of bullying have been measured in relation to psychosis outcomes. However, our comprehensive literature search strategy was designed to ensure that the significant literature on childhood adversity and psychosis was captured.

## Conclusion

This systematic review indicates a need for a standardized and universally accepted set of definitions and terminologies of childhood adversity to improve the reliability and validity of research into associations with psychosis. While there is a noticeable trend toward the use of validated instruments such as the CTQ-short, it appears that a broader spectrum of instruments is needed to capture the wide range of adversities and other characteristics of adversity that children may experience. Over-reliance on specific types of adversities without adequately addressing others has resulted in a skewed narrative around the association between childhood adversity and psychosis.

## Supplementary Material

Supplementary material is available at https://academic.oup.com/schizophreniabulletin/.

sbae085_suppl_Supplementary_Tables_1-4

sbae085_suppl_Supplementary_Tables_5
