# Supplementary material for: Unraveling the Concept of Childhood Adversity in Psychosis Research: A Systematic Review
Source: Schizophr Bull. 2024 May 30;50(5):1055–66. doi: 10.1093/schbul/sbae085 (PMC11349006; doi:10.1093/schbul/sbae085)
Supplement: sbae085_suppl_Supplementary_Tables_1-4 [file sbae085_suppl_supplementary_tables_1-4.docx]

# Supplementary Materials

**Table 1:** Search history …………………………………...…………………............................................................................... **2 – 16**

**Table 2:** Instrument characteristics…………………...…………………...................................................................................... **17 – 24**

**Table 3:** Refence list for included studies…………………...…………………............................................................................ **25 – 38**

**Table 4:** Refence list for excluded studies with reason……...…………………............................................................................ **39 – 52**

**Table 1** Search histories for initial and updated search

Both first search and update search strategies are listed for the searches in the databases PsycInfo, Embase and CINAHL. For PsycInfo and Embase, there had been updates to their thesauri at the time of the update search, and searches were edited accordingly. The search strategy for CINAHL is shown twice due to the way search strategies are printed, to show the specifics for both first search and update search.

**PsycInfo Ovid, March 2022, first search**

| 1 | psychosis/ or exp acute psychosis/ or affective psychosis/ or exp childhood psychosis/ or chronic psychosis/ or exp schizophrenia/ or exp "paranoia (psychosis)"/ or prodrome/ |
| --- | --- |
| 2 | hallucinations/ or auditory hallucinations/ or hypnagogic hallucinations/ or visual hallucinations/ or hallucinosis/ |
| 3 | thought disturbances/ or confabulation/ or delusions/ or "fantasies (thought disturbances)"/ or "fragmentation (schizophrenia)"/ |
| 4 | "3213".cc. |
| 5 | (psychos#s or schizo* or psychotic* or paranoi* or prodrom* or delusion* or hallucin* or thought disturbance? or thought disorder? or confabulation* or attenuated psycho* or attenuated symptom* or subclinical symptom* or sub-clinical symptom* or "at risk mental state*" or FEP).ab,ti,id. |
| 6 | 1 or 2 or 3 or 4 or 5 |
| 7 | childhood adversity/ |
| 8 | child abuse/ or child neglect/ |
| 9 | physical abuse/ |
| 10 | emotional abuse/ |
| 11 | sexual abuse/ or incest/ or rape/ |
| 12 | (violence/ or domestic violence/ or intimate partner violence/ or aggressive behavior/) and (witness* or expos*).ab,ti,id. |
| 13 | exposure to violence/ |
| 14 | bullying/ or cyberbullying/ or victimization/ |
| 15 | punishment/ or physical discipline/ |
| 16 | trauma/ or emotional trauma/ or trauma reactions/ or traumatic experiences/ or traumatic loss/ |
| 17 | disasters/ or natural disasters/ or accidents/ or war/ or terrorism/ |
| 18 | 9 or 10 or 11 or 12 or 13 or 14 or 15 or 16 or 17 |
| 19 | child*.ab,ti,id. |
| 20 | 18 and 19 |
| 21 | limit 18 to childhood <birth to 12 years> |
| 22 | 20 or 21 |
| 23 | parental death/ or parental absence/ or father absence/ or mother absence/ |
| 24 | (child* adj11 (adversit* or (adverse* adj3 experience*) or trauma* or psychotrauma* or abus* or assault* or rape? or incest* or maltreat* or exploit* or molest* or neglect* or punish* or bully* or cyberbull* or stressful life event* or loss* or victim* or physical discipl* or disaster* or accident or accidents or war or wars or terror* or catastroph*)).ab,ti,id. |
| 25 | (adversit* or (adverse* adj3 experience*) or trauma* or psychotrauma* or abus* or assault* or rape? or incest* or maltreat* or exploit* or molest* or neglect* or punish* or bully* or cyberbull* or stressful life event* or loss* or victim* or physical discipl* or disaster* or accident or accidents or war or wars or terror* or catastroph*).ab,ti,id. |
| 26 | limit 25 to childhood |
| 27 | ((violen* or spous* abus* or aggress* or agonistic*) and (witness* or expos*) and child*).ab,ti,id. |
| 28 | ((violen* or spous* abus* or aggress* or agonistic*) and (witness* or expos*)).ab,ti,id. |
| 29 | limit 28 to childhood |
| 30 | ((parent* or paternal or maternal or mother* or father*) adj6 (death* or absen* or separate? or separation? or depriv* or abandon*)).ti,ab,id. |
| 31 | (family adj6 (separate? or separation? or depriv* or abandon*)).ti,ab,id. |
| 32 | 7 or 8 or 22 or 23 or 24 or 26 or 27 or 29 or 30 or 31 |
| 33 | 6 and 32 |
| 34 | limit 33 to (journal article or retraction or reviews or "erratum/correction") |
| 35 | limit 34 to animal |
| 36 | limit 34 to human |
| 37 | 35 not 36 |
| 38 | (animal model* or mouse or mice or rat or rats).mp. |
| 39 | 34 and 38 |
| 40 | 39 not 36 |
| 41 | 37 or 40 |
| 42 | 34 not 41 |
| 43 | limit 42 to yr="2010 - 2022" |
| 44 | remove duplicates from 43 |
| 45 | limit 42 to yr="1860 - 2009" |
| 46 | remove duplicates from 45 |

**PsycInfo Ovid, October 2023, update search, terms updated due to changes in APA’s Thesaurus of Psychological Index Terms**

| 1 | psychosis/ or brief psychotic disorder/ or affective psychosis/ or exp childhood onset psychosis/ or chronic psychosis/ or exp schizophrenia/ or exp paranoid psychosis/ or prodrome/ |
| --- | --- |
| 2 | hallucinations/ or auditory hallucinations/ or hypnagogic hallucinations/ or visual hallucinations/ or hallucinosis/ |
| 3 | thought disorders/ or confabulation/ or delusions/ or "fantasies (thought disturbances)"/ or "fragmentation (schizophrenia)"/ |
| 4 | "3213".cc. |
| 5 | (psychos#s or schizo* or psychotic* or paranoi* or prodrom* or delusion* or hallucin* or thought disturbance? or thought disorder? or confabulation* or attenuated psycho* or attenuated symptom* or subclinical symptom* or sub-clinical symptom* or "at risk mental state*" or FEP).ab,ti,id. |
| 6 | 1 or 2 or 3 or 4 or 5 |
| 7 | childhood adversity/ |
| 8 | child abuse/ or child neglect/ |
| 9 | physical abuse/ |
| 10 | emotional abuse/ |
| 11 | sexual abuse/ or incest/ or rape/ |
| 12 | (violence/ or domestic violence/ or intimate partner violence/ or aggressive behavior/) and (witness* or expos*).ab,ti,id. |
| 13 | exposure to violence/ |
| 14 | bullying/ or cyberbullying/ or victimization/ |
| 15 | punishment/ or physical discipline/ |
| 16 | trauma/ or emotional trauma/ or trauma reactions/ or traumatic experiences/ or traumatic loss/ |
| 17 | disasters/ or natural disasters/ or accidents/ or war/ or terrorism/ |
| 18 | 9 or 10 or 11 or 12 or 13 or 14 or 15 or 16 or 17 |
| 19 | child*.ab,ti,id. |
| 20 | 18 and 19 |
| 21 | limit 18 to childhood <birth to 12 years> |
| 22 | 20 or 21 |
| 23 | parental death/ or parental absence/ or father absence/ or mother absence/ |
| 24 | (child* adj11 (adversit* or (adverse* adj3 experience*) or trauma* or psychotrauma* or abus* or assault* or rape? or incest* or maltreat* or exploit* or molest* or neglect* or punish* or bully* or cyberbull* or stressful life event* or loss* or victim* or physical discipl* or disaster* or accident or accidents or war or wars or terror* or catastroph*)).ab,ti,id. |
| 25 | (adversit* or (adverse* adj3 experience*) or trauma* or psychotrauma* or abus* or assault* or rape? or incest* or maltreat* or exploit* or molest* or neglect* or punish* or bully* or cyberbull* or stressful life event* or loss* or victim* or physical discipl* or disaster* or accident or accidents or war or wars or terror* or catastroph*).ab,ti,id. |
| 26 | limit 25 to childhood |
| 27 | ((violen* or spous* abus* or aggress* or agonistic*) and (witness* or expos*) and child*).ab,ti,id. |
| 28 | ((violen* or spous* abus* or aggress* or agonistic*) and (witness* or expos*)).ab,ti,id. |
| 29 | limit 28 to childhood |
| 30 | ((parent* or paternal or maternal or mother* or father*) adj6 (death* or absen* or separate? or separation? or depriv* or abandon*)).ti,ab,id. |
| 31 | (family adj6 (separate? or separation? or depriv* or abandon*)).ti,ab,id. |
| 32 | 7 or 8 or 22 or 23 or 24 or 26 or 27 or 29 or 30 or 31 |
| 33 | 6 and 32 |
| 34 | limit 33 to (journal article or retraction or reviews or "erratum/correction") |
| 35 | limit 34 to animal |
| 36 | limit 34 to human |
| 37 | 35 not 36 |
| 38 | (animal model* or mouse or mice or rat or rats).mp. |
| 39 | 34 and 38 |
| 40 | 39 not 36 |
| 41 | 37 or 40 |
| 42 | 34 not 41 |
| 43 | limit 42 to yr="2010 - 2024" |
| 44 | limit 43 to up="20220309-20231017" |

**Medline Ovid, searches March 2022 and October 2023**

| 1 | "schizophrenia spectrum and other psychotic disorders"/ or affective disorders, psychotic/ or paranoid disorders/ or psychotic disorders/ or schizophrenia/ or schizophrenia, catatonic/ or schizophrenia, disorganized/ or schizophrenia, paranoid/ or shared paranoid disorder/ or hallucinations/ or delusion/ or paranoid behavior/ |
| --- | --- |
| 2 | (psychos#s or schizo* or psychotic* or paranoi* or prodrom* or delusion* or hallucin* or thought disturbance? or thought disorder? or confabulation* or attenuated psycho* or attenuated symptom* or subclinical symptom* or sub-clinical symptom* or "at risk mental state*" or FEP).ab,ti,kf. |
| 3 | 1 or 2 |
| 4 | adverse childhood experiences/ or child abuse/ or child abuse, sexual/ |
| 5 | bullying/ or cyberbullying/ or emotional abuse/ or incest/ or physical abuse/ or rape/ or bullying/ or punishment/ or psychological trauma/ or historical trauma/ or sexual trauma/ |
| 6 | (violence/ or domestic violence/ or spouse abuse/ or intimate partner violence/ or aggression/) and (witness* or expos*).ti,ab,kf. |
| 7 | exposure to violence/ |
| 8 | disasters/ or exp natural disasters/ or "warfare and armed conflicts"/ or armed conflicts/ or accidents/ or terrorism/ |
| 9 | maternal deprivation/ or paternal deprivation/ or parental death/ or maternal death/ |
| 10 | child*.ti,ab,kf. |
| 11 | 5 or 6 or 7 or 8 |
| 12 | 10 and 11 |
| 13 | limit 11 to ("infant (1 to 23 months)" or "preschool child (2 to 5 years)" or "child (6 to 12 years)") |
| 14 | 12 or 13 |
| 15 | (child* adj11 (adversit* or (adverse* adj3 experience*) or trauma* or psychotrauma* or abus* or assault* or rape? or incest* or maltreat* or exploit* or molest* or neglect* or punish* or bully* or cyberbull* or stressful life event* or loss* or victim* or physical discipl* or disaster* or accident or accidents or war or wars or terror* or catastroph*)).ab,ti,kf. |
| 16 | (adversit* or (adverse* adj3 experience*) or trauma* or psychotrauma* or abus* or assault* or rape? or incest* or maltreat* or exploit* or molest* or neglect* or punish* or bully* or cyberbull* or stressful life event* or loss* or victim* or physical discipl* or disaster* or accident or accidents or war or wars or terror* or catastroph*).ab,ti,kf. |
| 17 | limit 16 to ("infant (1 to 23 months)" or "preschool child (2 to 5 years)" or "child (6 to 12 years)") |
| 18 | ((violen* or partner violen* or spous* abus* or aggress* or agonistic*) and (witness* or expos*) and child*).ab,ti,kf. |
| 19 | ((violen* or partner violen* or spous* abus* or aggress* or agonistic*) and (witness* or expos*)).ab,ti,kf. |
| 20 | limit 19 to ("infant (1 to 23 months)" or "preschool child (2 to 5 years)" or "child (6 to 12 years)") |
| 21 | ((parent* or paternal or maternal or mother* or father*) adj6 (death* or absen* or separate? or separation? or depriv* or abandon*)).ti,ab,kf. |
| 22 | (family adj6 (separate? or separation? or depriv* or abandon*)).ti,ab,kf. |
| 23 | 4 or 9 or 14 or 15 or 17 or 18 or 20 or 21 or 22 |
| 24 | 3 and 23 |
| 25 | limit 24 to ("corrected and republished article" or festschrift or journal article or retracted publication or "retraction of publication" or "review" or "systematic review" or "case reports" or "introductory journal article" or "published erratum") |
| 26 | 25 not (exp animals/ not humans.sh.) |
| 27 | (animal model* or mouse or mice or rat or rats).mp. |
| 28 | 25 and humans.sh. |
| 29 | 25 and 27 |
| 30 | 29 not 28 |
| 31 | 25 not 30 |
| 32 | 26 or 31 |
| 33 | limit 32 to yr="2010 - 2022" |
| 34 | remove duplicates from 33 |
|  |  |
|  | Last lines for update search were as follows |
| 33 | limit 32 to yr="2010 - 2024" |
| 34 | limit 33 to (dt=20220309-20231017 or rd=20220309-20231017) |
| 35 | remove duplicates from 34 |

**EMBASE Ovid, March 2022, first search**

| 1 | psychosis/ or acute psychosis/ or affective psychosis/ or brief psychotic disorder/ or childhood psychosis/ or manic psychosis/ or paranoid psychosis/ or exp schizophrenia/ or schizophrenia spectrum disorder/ or schizoaffective disorder/ or paranoid psychosis/ or paranoia/ or paranoid schizophrenia/ or delusion/ or delusional disorder/ or somatic delusion/ or schizophrenia spectrum disorder/ or hallucination/ or auditory hallucination/ or gustatory hallucination/ or hallucinosis/ or hypnagogic hallucination/ or olfactory hallucination/ or visual hallucination/ or thought disorder/ or confabulation/ |
| --- | --- |
| 2 | prodromal symptom/ |
| 3 | (psychos#s or schizo* or psychotic* or paranoi* or prodrom* or delusion* or hallucin* or thought disturbance? or thought disorder? or confabulation* or attenuated psycho* or attenuated symptom* or subclinical symptom* or sub-clinical symptom* or "at risk mental state*" or FEP).ab,ti,kf. |
| 4 | 1 or 2 or 3 |
| 5 | childhood adversity/ |
| 6 | child abuse/ or child neglect/ or child sexual abuse/ |
| 7 | child abuse survivor/ or childhood sexual abuse survivor/ |
| 8 | childhood trauma/ |
| 9 | childhood trauma survivor/ |
| 10 | physical abuse/ |
| 11 | emotional abuse/ or emotional neglect/ |
| 12 | sexual assault/ or sexual violence/ or rape/ or sexual abuse/ or incest/ or sexual trauma/ or exposure to violence/ or psychotrauma/ |
| 13 | bullying/ or cyberbullying/ |
| 14 | (violence/ or domestic violence/ or exp partner violence/ or family violence/ or aggression/) and (witness* or expos*).ti,ab,kf. |
| 15 | disaster/ or natural disaster/ or accident/ or war/ or exp terrorism/ |
| 16 | parental death/ or maternal death/ or parental deprivation/ or emotional deprivation/ |
| 17 | 10 or 11 or 12 or 13 or 14 or 15 |
| 18 | child*.ti,ab,kf. |
| 19 | 17 and 18 |
| 20 | limit 17 to (infant or preschool child <1 to 6 years> or school child <7 to 12 years>) |
| 21 | (child* adj11 (adversit* or (adverse* adj3 experience*) or trauma* or psychotrauma* or abus* or assault* or rape? or incest* or maltreat* or exploit* or molest* or neglect* or punish* or bully* or cyberbull* or stressful life event* or loss* or victim* or physical discipl* or disaster* or accident or accidents or war or wars or terror* or catastroph*)).ab,ti,kf. |
| 22 | (adversit* or (adverse* adj3 experience*) or trauma* or psychotrauma* or abus* or assault* or rape? or incest* or maltreat* or exploit* or molest* or neglect* or punish* or bully* or cyberbull* or stressful life event* or loss* or victim* or physical discipl* or disaster* or accident or accidents or war or wars or terror* or catastroph*).ab,ti,kf. |
| 23 | limit 22 to (infant or preschool child <1 to 6 years> or school child <7 to 12 years>) |
| 24 | ((violen* or spous* abus* or aggress* or agonistic*) and (witness* or expos*) and child*).ab,ti,kf. |
| 25 | ((violen* or spous* abus* or aggress* or agonistic*) and (witness* or expos*)).ab,ti,kf. 29232 |
| 26 | limit 25 to (infant or preschool child <1 to 6 years> or school child <7 to 12 years>) |
| 27 | ((parent* or paternal or maternal or mother* or father*) adj6 (death* or absen* or separate? or separation? or depriv* or abandon*)).ti,ab,kf. |
| 28 | (family adj6 (separate? or separation? or depriv* or abandon*)).ti,ab,kf. |
| 29 | 5 or 6 or 7 or 8 or 9 or 16 or 19 or 20 or 21 or 23 or 24 or 26 or 27 or 28 |
| 30 | 4 and 29 |
| 31 | 30 not ((exp animal/ or nonhuman/) not exp human/) |
| 32 | (animal model* or mouse or mice or rat or rats).ti,kf. |
| 33 | 30 and 32 |
| 34 | 30 not 31 |
| 35 | 33 or 34 |
| 36 | 30 not 35 |
| 37 | limit 36 to embase |
| 38 | limit 37 to (article or article in press or "review") |
| 39 | limit 38 to yr="2010 - 2022" |
| 40 | remove duplicates from 39 |

**Embase** **Ovid, October 2023, update search, terms updated due to changes in Emtree thesaurus**

| 1 | exp psychotrauma/ or psychosis/ or acute psychosis/ or affective psychosis/ or brief psychotic disorder/ or childhood psychosis/ or manic psychosis/ or paranoid psychosis/ or exp schizophrenia/ or schizophrenia spectrum disorder/ or schizoaffective disorder/ or paranoid psychosis/ or paranoia/ or paranoid schizophrenia/ or delusion/ or delusional disorder/ or somatic delusion/ or schizophrenia spectrum disorder/ or hallucination/ or auditory hallucination/ or gustatory hallucination/ or hallucinosis/ or hypnagogic hallucination/ or olfactory hallucination/ or visual hallucination/ or thought disorder/ or confabulation/ |
| --- | --- |
| 2 | prodromal symptom/ |
| 3 | (psychos#s or schizo* or psychotic* or paranoi* or prodrom* or delusion* or hallucin* or thought disturbance? or thought disorder? or confabulation* or attenuated psycho* or attenuated symptom* or subclinical symptom* or sub-clinical symptom* or "at risk mental state*" or FEP).ab,ti,kf. |
| 4 | 1 or 2 or 3 |
| 5 | childhood adversity/ |
| 6 | child abuse/ or child neglect/ or child sexual abuse/ |
| 7 | child abuse survivor/ or childhood sexual abuse survivor/ |
| 8 | childhood trauma/ |
| 9 | childhood trauma survivor/ |
| 10 | physical abuse/ |
| 11 | emotional abuse/ or emotional neglect/ |
| 12 | sexual assault/ or sexual violence/ or rape/ or sexual abuse/ or incest/ or sexual trauma/ or exposure to violence/ or psychotrauma/ |
| 13 | bullying/ or cyberbullying/ |
| 14 | (violence/ or domestic violence/ or exp partner violence/ or family violence/ or aggression/) and (witness* or expos*).ti,ab,kf. |
| 15 | disaster/ or natural disaster/ or accident/ or war/ or exp terrorism/ |
| 16 | parental death/ or maternal death/ or parental deprivation/ or emotional deprivation/ |
| 17 | 10 or 11 or 12 or 13 or 14 or 15 |
| 18 | child*.ti,ab,kf. |
| 19 | 17 and 18 |
| 20 | limit 17 to (infant or preschool child <1 to 6 years> or school child <7 to 12 years>) |
| 21 | (child* adj11 (adversit* or (adverse* adj3 experience*) or trauma* or psychotrauma* or abus* or assault* or rape? or incest* or maltreat* or exploit* or molest* or neglect* or punish* or bully* or cyberbull* or stressful life event* or loss* or victim* or physical discipl* or disaster* or accident or accidents or war or wars or terror* or catastroph*)).ab,ti,kf. |
| 22 | (adversit* or (adverse* adj3 experience*) or trauma* or psychotrauma* or abus* or assault* or rape? or incest* or maltreat* or exploit* or molest* or neglect* or punish* or bully* or cyberbull* or stressful life event* or loss* or victim* or physical discipl* or disaster* or accident or accidents or war or wars or terror* or catastroph*).ab,ti,kf. |
| 23 | limit 22 to (infant or preschool child <1 to 6 years> or school child <7 to 12 years>) |
| 24 | ((violen* or spous* abus* or aggress* or agonistic*) and (witness* or expos*) and child*).ab,ti,kf. |
| 25 | ((violen* or spous* abus* or aggress* or agonistic*) and (witness* or expos*)).ab,ti,kf. 29232 |
| 26 | limit 25 to (infant or preschool child <1 to 6 years> or school child <7 to 12 years>) |
| 27 | ((parent* or paternal or maternal or mother* or father*) adj6 (death* or absen* or separate? or separation? or depriv* or abandon*)).ti,ab,kf. |
| 28 | (family adj6 (separate? or separation? or depriv* or abandon*)).ti,ab,kf. |
| 29 | 5 or 6 or 7 or 8 or 9 or 16 or 19 or 20 or 21 or 23 or 24 or 26 or 27 or 28 |
| 30 | 4 and 29 |
| 31 | 30 not ((exp animal/ or nonhuman/) not exp human/) |
| 32 | (animal model* or mouse or mice or rat or rats).ti,kf. |
| 33 | 30 and 32 |
| 34 | 30 not 31 |
| 35 | 33 or 34 |
| 36 | 30 not 35 |
| 37 | limit 36 to embase |
| 38 | limit 37 to (article or article in press or "review") |
| 39 | limit 38 to yr="2010 - 2024" |
| 40 | limit 39 to (dd=20220309-20231017 or rd=20220309-20231017) |
| 41 | remove duplicates from 40 |

**CINAHL EBSCO, March 2022, first search**

| S30 | S3 AND S28 | Limiters - Published Date: 20100101-20221231; Exclude MEDLINE records; Age Groups: Infant: 1-23 months, Child, Preschool: 2-5 years, Child: 6-12 years, All Child Search modes - Boolean/Phrase |
| --- | --- | --- |
| S29 | S3 AND S28 | Limiters - Exclude MEDLINE records Search modes - Boolean/Phrase |
| S28 | S4 OR S5 OR S18 OR S19 OR S20 OR S21 OR S22 OR S23 OR S26 OR S27 | Search modes - Boolean/Phrase |
| S27 | (TI adversit* OR AB adversit*) OR (TI adverse* N2 experience*) or (AB adverse* N2 experience*) or (TI trauma* OR AB trauma*) OR (TI psychotrauma* OR AB psychotrauma*) OR (TI abus* OR AB abus*) OR (TI assault* OR AB assault*) OR (TI rape* OR AB rape*) OR (TI incest* OR AB incest*) OR (TI maltreat* OR AB maltreat*) OR (TI exploit* OR AB exploit*) OR (TI molest* OR AB molest*) OR (TI neglect* OR AB neglect*) OR (TI punish* OR AB punish*) OR (TI bully* OR AB bully*) OR (TI victim* OR AB victim*) OR (TI cyberbull* OR AB cyberbull*) OR (TI "stressful life event*" OR AB "stressful life event*") OR (TI loss* OR AB loss*) OR (TI "physical discipl*" OR AB "physical discipl*") OR (TI disaster* OR AB disaster*) OR (TI accident or AB accident) OR (TI accidents or AB accidents) OR (TI war or AB war) OR (TI wars or AB wars) OR (TI terror* or AB terror*) OR (TI catastroph* or AB catastroph*) | Limiters - Age Groups: Infant: 1-23 months, Child, Preschool: 2-5 years, Child: 6-12 years, All Child Search modes - Boolean/Phrase |
| S26 | S24 OR S25 | Limiters - Published Date: 20100101-20221231  Search modes - Boolean/Phrase |
| S25 | ((TI violen* or AB violen*) or (TI spous* abus* OR AB spous* abus*) or (TI aggress* or AB aggress*) or (TI agonistic* or AB agonistic*)) and ((TI witness* or AB witness*) OR (TI expos* or AB expos*)) | Limiters - Age Groups: Infant: 1-23 months, Child, Preschool: 2-5 years, Child: 6-12 years, All Child Search modes - Boolean/Phrase |
| S24 | ((TI violen* or AB violen*) or (TI spous* abus* OR AB spous* abus*) or (TI aggress* or AB aggress*) or (TI agonistic* or AB agonistic*)) and ((TI witness* or AB witness*) OR (TI expos* or AB expos*)) and (TI child* or AB child*) | Search modes - Boolean/Phrase |
| S23 | (TI family OR AB family) N5 ((TI "separate" OR AB "separate") OR (TI "separated" or AB "separated") OR (TI separation* OR AB separation*) OR (TI depriv* OR AB depriv*) OR (TI abandon* OR AB abandon*)) | Search modes - Boolean/Phrase |
| S22 | ((TI parent* OR AB parent*) OR (TI paternal OR AB paternal) OR (TI maternal or AB maternal) OR (TI mother* OR AB mother*) OR (TI father* OR AB father*)) N5 ((TI death* OR AB death*) OR (TI absen* OR AB absen*) OR (TI "separate" OR TI "separated" OR TI separation*) OR (AB "separate" OR AB "separated" OR AB separation*) OR (TI depriv* OR AB depriv*) OR (TI abandon* OR AB abandon*)) | Search modes - Boolean/Phrase |
| S21 | (TI child* OR AB child*) N10 ((TI adversit* OR AB adversit*) OR (TI adverse* N2 experience*) or (AB adverse* N2 experience*) or (TI trauma* OR AB trauma*) OR (TI psychotrauma* OR AB psychotrauma*) OR (TI abus* OR AB abus*) OR (TI assault* OR AB assault*) OR (TI rape* OR AB rape*) OR (TI incest* OR AB incest*) OR (TI maltreat* OR AB maltreat*) OR (TI exploit* OR AB exploit*) OR (TI molest* OR AB molest*) OR (TI neglect* OR AB neglect*) OR (TI punish* OR AB punish*) OR (TI bully* OR AB bully*) OR (TI victim* OR AB victim*) OR (TI cyberbull* OR AB cyberbull*) OR (TI "stressful life event*" OR AB "stressful life event*") OR (TI loss* OR AB loss*) OR (TI "physical discipl*" OR AB "physical discipl*") OR (TI disaster* OR AB disaster*) OR (TI accident or AB accident) OR (TI accidents or AB accidents) OR (TI war or AB war) OR (TI wars or AB wars) OR (TI terror* or AB terror*) OR (TI catastroph* or AB catastroph*)) | Search modes - Boolean/Phrase |
| S20 | (MH "Family Separation") | Search modes - Boolean/Phrase |
| S19 | (MH "Parental Death") | Search modes - Boolean/Phrase |
| S18 | S16 OR S17 | Search modes - Boolean/Phrase |
| S17 | S6 OR S9 OR S10 OR S11 OR S12 OR S13 | Limiters - Age Groups: Infant: 1-23 months, Child, Preschool: 2-5 years, Child: 6-12 years, All Child Search modes - Boolean/Phrase |
| S16 | S14 AND S15 | Search modes - Boolean/Phrase |
| S15 | (TI child* OR AB child*) | Search modes - Boolean/Phrase |
| S14 | S6 OR S9 OR S10 OR S11 OR S12 OR S13 | Search modes - Boolean/Phrase |
| S13 | (MH "Natural Disasters") OR (MH "Disasters") OR (MH "Accidents") OR (MH "War") OR (MH "Terrorism") | Search modes - Boolean/Phrase |
| S12 | (MH "Punishment") | Search modes - Boolean/Phrase |
| S11 | (MH "Bullying") OR (MH "Cyberbullying") | Search modes - Boolean/Phrase |
| S10 | (MH "Sexual Abuse") OR (MH "Incest") OR (MH "Rape") | Search modes - Boolean/Phrase |
| S9 | S7 AND S8 | Search modes - Boolean/Phrase |
| S8 | (TI witness* OR AB witness*) or (TI expos* OR AB expos*) | Search modes - Boolean/Phrase |
| S7 | (MH "Domestic Violence") OR (MH "Violence") OR (MH "Intimate Partner Violence") OR (MH "Aggression") | Search modes - Boolean/Phrase |
| S6 | (MH "Historical Trauma") OR (MH "Sexual Trauma") OR (MH "Psychological Trauma") OR (MH "Emotional Abuse") | Search modes - Boolean/Phrase |
| S5 | (MH "Child Abuse Survivors") | Search modes - Boolean/Phrase |
| S4 | (MH "Adverse Childhood Experiences") OR (MH "Child Abuse") OR (MH "Child Abuse, Sexual") | Search modes - Boolean/Phrase |
| S3 | S1 OR S2 | Search modes - Boolean/Phrase |
| S2 | (MH "Psychotic Disorders") OR (MH "Affective Disorders, Psychotic") OR (MH "Delusions") OR (MH "Delusions, Parasitosis") OR (MH "Paranoid Disorders") OR (MH "Schizophrenia") OR (MH "Schizophrenia, Childhood") OR (MH "Schizoaffective Disorder") OR (MH "Hallucinations") | Search modes - Boolean/Phrase |
| S1 | ((TI psychos?s OR AB psychos?s) OR (TI schizo* OR AB schizo*) OR (TI psychotic* OR AB psychotic*) OR (TI paranoi* OR AB paranoi*) OR (TI prodrom* OR AB prodrom*) OR (TI delusion* OR AB delusion*) OR (TI hallucin* OR AB hallucin*) OR (TI "thought disturbance*" OR AB "thought disturbance*") OR (TI "thought disorder*" OR AB "thought disorder*") OR (TI confabulation* OR AB confabulation*) OR (TI "attenuated symptom*" OR AB "attenuated symptom*") OR (TI "attenuated psycho*" OR AB "attenuated psycho*") OR (TI "subclinical symptom*" OR AB "subclinical symptom*") OR (TI "sub-clinical symptom*" OR AB "sub-clinical symptom*") OR (TI "at risk mental state*" OR AB "at risk mental state*") OR (TI "FEP" OR AB "FEP")) | Search modes - Boolean/Phrase |

**CINAHL EBSCO, October 2023, update search**

| \| S32 \| S30 AND S31 \| Search modes - Boolean/Phrase \| \| --- \| --- \| --- \| \| S31 \| EM 202203- OR RD 202203- \| Limiters - Exclude MEDLINE records Search modes - Boolean/Phrase \| \| S30 \| S3 AND S28 \| Limiters - Published Date: 20100101-20231031; Exclude MEDLINE records; Age Groups: Infant: 1-23 months, Child, Preschool: 2-5 years, Child: 6-12 years, All Child Search modes - Boolean/Phrase \| \| S29 \| S3 AND S28 \| Limiters - Exclude MEDLINE records Search modes - Boolean/Phrase \| \| S28 \| S4 OR S5 OR S18 OR S19 OR S20 OR S21 OR S22 OR S23 OR S26 OR S27 \| Search modes - Boolean/Phrase \| \| S27 \| (TI adversit* OR AB adversit*) OR (TI adverse* N2 experience*) or (AB adverse* N2 experience*) or (TI trauma* OR AB trauma*) OR (TI psychotrauma* OR AB psychotrauma*) OR (TI abus* OR AB abus*) OR (TI assault* OR AB assault*) OR (TI rape* OR AB rape*) OR (TI incest* OR AB incest*) OR (TI maltreat* OR AB maltreat*) OR (TI exploit* OR AB exploit*) OR (TI molest* OR AB molest*) OR (TI neglect* OR AB neglect*) OR (TI punish* OR AB punish*) OR (TI bully* OR AB bully*) OR (TI victim* OR AB victim*) OR (TI cyberbull* OR AB cyberbull*) OR (TI "stressful life event*" OR AB "stressful life event*") OR (TI loss* OR AB loss*) OR (TI "physical discipl*" OR AB "physical discipl*") OR (TI disaster* OR AB disaster*) OR (TI accident or AB accident) OR (TI accidents or AB accidents) OR (TI war or AB war) OR (TI wars or AB wars) OR (TI terror* or AB terror*) OR (TI catastroph* or AB catastroph*) \| Limiters - Age Groups: Infant: 1-23 months, Child, Preschool: 2-5 years, Child: 6-12 years, All Child Search modes - Boolean/Phrase \| \| S26 \| S24 OR S25 \| Limiters - Published Date: 20100101-20231031  Search modes - Boolean/Phrase \| \| S25 \| ((TI violen* or AB violen*) or (TI spous* abus* OR AB spous* abus*) or (TI aggress* or AB aggress*) or (TI agonistic* or AB agonistic*)) and ((TI witness* or AB witness*) OR (TI expos* or AB expos*)) \| Limiters - Age Groups: Infant: 1-23 months, Child, Preschool: 2-5 years, Child: 6-12 years, All Child Search modes - Boolean/Phrase \| \| S24 \| ((TI violen* or AB violen*) or (TI spous* abus* OR AB spous* abus*) or (TI aggress* or AB aggress*) or (TI agonistic* or AB agonistic*)) and ((TI witness* or AB witness*) OR (TI expos* or AB expos*)) and (TI child* or AB child*) \| Search modes - Boolean/Phrase \| \| S23 \| (TI family OR AB family) N5 ((TI "separate" OR AB "separate") OR (TI "separated" or AB "separated") OR (TI separation* OR AB separation*) OR (TI depriv* OR AB depriv*) OR (TI abandon* OR AB abandon*)) \| Search modes - Boolean/Phrase \| \| S22 \| ((TI parent* OR AB parent*) OR (TI paternal OR AB paternal) OR (TI maternal or AB maternal) OR (TI mother* OR AB mother*) OR (TI father* OR AB father*)) N5 ((TI death* OR AB death*) OR (TI absen* OR AB absen*) OR (TI "separate" OR TI "separated" OR TI separation*) OR (AB "separate" OR AB "separated" OR AB separation*) OR (TI depriv* OR AB depriv*) OR (TI abandon* OR AB abandon*)) \| Search modes - Boolean/Phrase \| \| S21 \| (TI child* OR AB child*) N10 ((TI adversit* OR AB adversit*) OR (TI adverse* N2 experience*) or (AB adverse* N2 experience*) or (TI trauma* OR AB trauma*) OR (TI psychotrauma* OR AB psychotrauma*) OR (TI abus* OR AB abus*) OR (TI assault* OR AB assault*) OR (TI rape* OR AB rape*) OR (TI incest* OR AB incest*) OR (TI maltreat* OR AB maltreat*) OR (TI exploit* OR AB exploit*) OR (TI molest* OR AB molest*) OR (TI neglect* OR AB neglect*) OR (TI punish* OR AB punish*) OR (TI bully* OR AB bully*) OR (TI victim* OR AB victim*) OR (TI cyberbull* OR AB cyberbull*) OR (TI "stressful life event*" OR AB "stressful life event*") OR (TI loss* OR AB loss*) OR (TI "physical discipl*" OR AB "physical discipl*") OR (TI disaster* OR AB disaster*) OR (TI accident or AB accident) OR (TI accidents or AB accidents) OR (TI war or AB war) OR (TI wars or AB wars) OR (TI terror* or AB terror*) OR (TI catastroph* or AB catastroph*)) \| Search modes - Boolean/Phrase \| \| S20 \| (MH "Family Separation") \| Search modes - Boolean/Phrase \| \| S19 \| (MH "Parental Death") \| Search modes - Boolean/Phrase \| \| S18 \| S16 OR S17 \| Search modes - Boolean/Phrase \| \| S17 \| S6 OR S9 OR S10 OR S11 OR S12 OR S13 \| Limiters - Age Groups: Infant: 1-23 months, Child, Preschool: 2-5 years, Child: 6-12 years, All Child Search modes - Boolean/Phrase \| \| S16 \| S14 AND S15 \| Search modes - Boolean/Phrase \| \| S15 \| (TI child* OR AB child*) \| Search modes - Boolean/Phrase \| \| S14 \| S6 OR S9 OR S10 OR S11 OR S12 OR S13 \| Search modes - Boolean/Phrase \| \| S13 \| (MH "Natural Disasters") OR (MH "Disasters") OR (MH "Accidents") OR (MH "War") OR (MH "Terrorism") \| Search modes - Boolean/Phrase \| \| S12 \| (MH "Punishment") \| Search modes - Boolean/Phrase \| \| S11 \| (MH "Bullying") OR (MH "Cyberbullying") \| Search modes - Boolean/Phrase \| \| S10 \| (MH "Sexual Abuse") OR (MH "Incest") OR (MH "Rape") \| Search modes - Boolean/Phrase \| \| S9 \| S7 AND S8 \| Search modes - Boolean/Phrase \| \| S8 \| (TI witness* OR AB witness*) or (TI expos* OR AB expos*) \| Search modes - Boolean/Phrase \| \| S7 \| (MH "Domestic Violence") OR (MH "Violence") OR (MH "Intimate Partner Violence") OR (MH "Aggression") \| Search modes - Boolean/Phrase \| \| S6 \| (MH "Historical Trauma") OR (MH "Sexual Trauma") OR (MH "Psychological Trauma") OR (MH "Emotional Abuse") \| Search modes - Boolean/Phrase \| \| S5 \| (MH "Child Abuse Survivors") \| Search modes - Boolean/Phrase \| \| S4 \| (MH "Adverse Childhood Experiences") OR (MH "Child Abuse") OR (MH "Child Abuse, Sexual") \| Search modes - Boolean/Phrase \| \| S3 \| S1 OR S2 \| Search modes - Boolean/Phrase \| \| S2 \| (MH "Psychotic Disorders") OR (MH "Affective Disorders, Psychotic") OR (MH "Delusions") OR (MH "Delusions, Parasitosis") OR (MH "Paranoid Disorders") OR (MH "Schizophrenia") OR (MH "Schizophrenia, Childhood") OR (MH "Schizoaffective Disorder") OR (MH "Hallucinations") \| Search modes - Boolean/Phrase \| \| S1 \| ((TI psychos?s OR AB psychos?s) OR (TI schizo* OR AB schizo*) OR (TI psychotic* OR AB psychotic*) OR (TI paranoi* OR AB paranoi*) OR (TI prodrom* OR AB prodrom*) OR (TI delusion* OR AB delusion*) OR (TI hallucin* OR AB hallucin*) OR (TI "thought disturbance*" OR AB "thought disturbance*") OR (TI "thought disorder*" OR AB "thought disorder*") OR (TI confabulation* OR AB confabulation*) OR (TI "attenuated symptom*" OR AB "attenuated symptom*") OR (TI "attenuated psycho*" OR AB "attenuated psycho*") OR (TI "subclinical symptom*" OR AB "subclinical symptom*") OR (TI "sub-clinical symptom*" OR AB "sub-clinical symptom*") OR (TI "at risk mental state*" OR AB "at risk mental state*") OR (TI "FEP" OR AB "FEP")) \| Search modes - Boolean/Phrase \| |
| --- | --- | --- | --- | --- | --- | --- | --- | --- | --- | --- | --- | --- | --- | --- | --- | --- | --- | --- | --- | --- | --- | --- | --- | --- | --- | --- | --- | --- | --- | --- | --- | --- | --- | --- | --- | --- | --- | --- | --- | --- | --- | --- | --- | --- | --- | --- | --- | --- | --- | --- | --- | --- | --- | --- | --- | --- | --- | --- | --- | --- | --- | --- | --- | --- | --- | --- | --- | --- | --- | --- | --- | --- | --- | --- | --- | --- | --- | --- | --- | --- | --- | --- | --- | --- | --- | --- | --- | --- | --- | --- | --- | --- | --- | --- | --- | --- |

**Web of Science Core Collection, searches March 2022 and October 2023, publication year limits were used as in the other searches**

| 9 | **#7 not #8 and Article or Review Article or Early Access (**Document Types) |
| --- | --- |
| 8 | **TS=("animal model*" or mouse or mice or rat or rats)** |
| 7 | **#5 AND #6** |
| 6 | **TS=(psychosis or psychoses or schizo* or psychotic* or prodrom* or paranoi* or delusion* or hallucin* or "thought disturbance*" or "thought disorder*" or confabulation* or "attenuated psycho*" or "attenuated symptom*" or "subclinical symptom*" or "sub-clinical symptom*" or "at risk mental state*" or "FEP")** |
| 5 | **#1 OR #2 OR #3 OR #4** |
| 4 | **TS=(family NEAR/5 (separate or separated or separation* or depriv* or abandon*))** |
| 3 | **TS=((parent* or paternal or maternal or mother* or father*) NEAR/5 (death* or absen* or separate or separated or separation* or depriv* or abandon*))** |
| 2 | **TS=((violen* or spous* abus* or aggress* or agonistic*) and (witness* or expos*) and child*)** |
| 1 | **TS=(child* NEAR/10 ((adverse* NEAR/2 experience*) OR adversit* or trauma* or psychotrauma* or abus* or assault* or rape* or incest* or maltreat* or exploit* or molest* or neglect* or punish* or bully* or cyberbull* or "stressful life event*" or loss* or "physical discipl*" or disaster* or accident or accidents or war or wars or terror* or catastroph*))** |

**SCOPUS, searches March 2022 and October 2023, publication year limits were used as in the other searches**

((((TITLE-ABS-KEY(psychosis or psychoses or schizo* or psychotic* or paranoi* or prodrom* or delusion* or hallucin* or {thought disturbance} or {though disturbances} or {thought disorder} or {thought disorders} or confabulation* or {attenuated psychosis} or {attenuated psychoses} or {attenuated symptom} or {attenuated symptoms} or {subclinical symptom} or {subclinical symptoms} or {sub-clinical symptom} or {sub-clinical symptoms} or {at risk mental state} or {at risk mental states} or "FEP")) and ((TITLE-ABS-KEY(child* W/10 (adversit* or (adverse* W/2 experience*) or trauma* or psychotrauma* or abus* or assault* or rape* or incest* or maltreat* or exploit* or molest* or neglect* or punish* or bully* or cyberbull* or "stressful life event" or "stressful life events" or loss* or victim* or "physical discipline" or disaster* or accident or accidents or war or wars or terror* or catastroph*))) or (TITLE-ABS-KEY((violen* or spous* abus* or aggress* or agonistic*) and (witness* or expos*) and child*)) or (TITLE-ABS-KEY((parent* or paternal or maternal or mother* or father*) W/5 (death* or absen* or "separate" or "separated" or separation* or depriv* or abandon*))) or (TITLE-ABS-KEY(family W/5 ("separate" or "separated" or separation* or depriv* or abandon*))))) and not (TITLE-ABS-KEY("animal model*" or mouse or mice or {rat} or {rats}))) AND NOT index)

**AIM - African Index Medicus, searches March 2022 and October 2023, publication year limits were used as in the other searches**

(psychosis OR psychoses OR schizo* OR psychotic* OR paranoi* OR prodrom* OR delusion* OR hallucin* OR "thought disturbance" OR "thought disturbances" OR "thought disorder" OR "thought disorders" OR confabulation* OR "attenuated psychotic" OR "attenuated psychosis" OR "attenuated psychoses" OR "attenuated symptom" OR "attenuated symptoms" OR "subclinical symptom" OR "subclinical symptoms" OR "sub-clinical symptom" OR "sub-clinical symptoms" OR "at risk mental state" OR "at risk mental states" OR fep) AND (advers* OR trauma* OR psychotrauma* OR abus* OR assault* OR rape* OR incest* OR maltreat* OR exploit* OR molest* OR neglect* OR punish* OR bully* OR cyberbull* OR “stressful life event” OR “stressful life events” OR loss* OR victim* OR “physical discipline” OR disaster* OR accident OR accidents OR war OR wars OR terror* OR catastroph*) AND (child*) AND ( db:("AIM"))

(psychosis OR psychoses OR schizo* OR psychotic* OR paranoi* OR prodrom* OR delusion* OR hallucin* OR "thought disturbance" OR "thought disturbances" OR "thought disorder" OR "thought disorders" OR confabulation* OR "attenuated psychotic" OR "attenuated psychosis" OR "attenuated psychoses" OR "attenuated symptom" OR "attenuated symptoms" OR "subclinical symptom" OR "subclinical symptoms" OR "sub-clinical symptom" OR "sub-clinical symptoms" OR "at risk mental state" OR "at risk mental states" OR fep) AND ((violen* OR spous* abus* OR aggress* OR agonistic*) AND (witness* OR expos*)) AND (child*) AND ( db:("AIM"))

(psychosis OR psychoses OR schizo* OR psychotic* OR paranoi* OR prodrom* OR delusion* OR hallucin* OR "thought disturbance" OR "thought disturbances" OR "thought disorder" OR "thought disorders" OR confabulation* OR "attenuated psychotic" OR "attenuated psychosis" OR "attenuated psychoses" OR "attenuated symptom" OR "attenuated symptoms" OR "subclinical symptom" OR "subclinical symptoms" OR "sub-clinical symptom" OR "sub-clinical symptoms" OR "at risk mental state" OR "at risk mental states" OR fep) AND ((parent* OR paternal OR maternal OR mother* OR father*) AND (death* OR absen* OR separat* OR depriv* OR abandon*)) AND (child*) AND ( db:("AIM"))

(psychosis OR psychoses OR schizo* OR psychotic* OR paranoi* OR prodrom* OR delusion* OR hallucin* OR "thought disturbance" OR "thought disturbances" OR "thought disorder" OR "thought disorders" OR confabulation* OR "attenuated psychotic" OR "attenuated psychosis" OR "attenuated psychoses" OR "attenuated symptom" OR "attenuated symptoms" OR "subclinical symptom" OR "subclinical symptoms" OR "sub-clinical symptom" OR "sub-clinical symptoms" OR "at risk mental state" OR "at risk mental states" OR fep) AND (family AND (separat* OR depriv* OR abandon*)) AND (child*) AND ( db:("AIM"))

**LILACS - Latin American and Caribbean Center on Health Sciences Information, searches March 2022 and October 2023, publication year limits were used as in the other searches**

(psychosis OR psychoses OR schizo* OR psychotic* OR paranoi* OR prodrom* OR delusion* OR hallucin* OR "thought disturbance" OR "thought disturbances" OR "thought disorder" OR "thought disorders" OR confabulation* OR "attenuated psychotic" OR "attenuated psychosis" OR "attenuated psychoses" OR "attenuated symptom" OR "attenuated symptoms" OR "subclinical symptom" OR "subclinical symptoms" OR "sub-clinical symptom" OR "sub-clinical symptoms" OR "at risk mental state" OR "at risk mental states" OR fep) AND (advers* OR trauma* OR psychotrauma* OR abus* OR assault* OR rape* OR incest* OR maltreat* OR exploit* OR molest* OR neglect* OR punish* OR bully* OR cyberbull* OR “stressful life event” OR “stressful life events” OR loss* OR victim* OR “physical discipline” OR disaster* OR accident OR accidents OR war OR wars OR terror* OR catastroph*) AND (child*) AND ( db:("LILACS"))

(psychosis OR psychoses OR schizo* OR psychotic* OR paranoi* OR prodrom* OR delusion* OR hallucin* OR "thought disturbance" OR "thought disturbances" OR "thought disorder" OR "thought disorders" OR confabulation* OR "attenuated psychotic" OR "attenuated psychosis" OR "attenuated psychoses" OR "attenuated symptom" OR "attenuated symptoms" OR "subclinical symptom" OR "subclinical symptoms" OR "sub-clinical symptom" OR "sub-clinical symptoms" OR "at risk mental state" OR "at risk mental states" OR fep) AND ((violen* OR spous* abus* OR aggress* OR agonistic*) AND (witness* OR expos*)) AND (child*) AND ( db:("LILACS"))

(psychosis OR psychoses OR schizo* OR psychotic* OR paranoi* OR prodrom* OR delusion* OR hallucin* OR "thought disturbance" OR "thought disturbances" OR "thought disorder" OR "thought disorders" OR confabulation* OR "attenuated psychotic" OR "attenuated psychosis" OR "attenuated psychoses" OR "attenuated symptom" OR "attenuated symptoms" OR "subclinical symptom" OR "subclinical symptoms" OR "sub-clinical symptom" OR "sub-clinical symptoms" OR "at risk mental state" OR "at risk mental states" OR fep) AND ((parent* OR paternal OR maternal OR mother* OR father*) AND (death* OR absen* OR separat* OR depriv* OR abandon*)) AND (child*) AND ( db:("LILACS"))

(psychosis OR psychoses OR schizo* OR psychotic* OR paranoi* OR prodrom* OR delusion* OR hallucin* OR "thought disturbance" OR "thought disturbances" OR "thought disorder" OR "thought disorders" OR confabulation* OR "attenuated psychotic" OR "attenuated psychosis" OR "attenuated psychoses" OR "attenuated symptom" OR "attenuated symptoms" OR "subclinical symptom" OR "subclinical symptoms" OR "sub-clinical symptom" OR "sub-clinical symptoms" OR "at risk mental state" OR "at risk mental states" OR fep) AND (family AND (separat* OR depriv* OR abandon*)) AND (child*) AND ( db:("LILACS"))

**Table 2.** Instrument characteristics

| **Trauma measure** | **Construct** | **Type of exposure** | **Structure** | **Admin** | **Format** | **N=Items** | **Validation study** | **Outcomes** | **Validated for psychosis** |
| --- | --- | --- | --- | --- | --- | --- | --- | --- | --- |
| Childhood Trauma Questionnaire  (CTQ) | Historical childhood trauma | Neglect (emotional, physical)  Abuse (emotional, sexual, physical) | Five scales | SR | 5-point Likert scale | 70 | Bernstein et al (1994). Initial reliability and validity of a new retrospective measure of child abuse and neglect. *The American journal of psychiatry*, *151*(8), 1132-1136.  Bernstein, D., & Fink, L. (1998). Childhood Trauma Questionnaire: A retrospective self-report*. San Antonio, TX: The Psychological Corporation*. | Total/Cut-off value = All items  Subscale/Cut-off= Each subscale | Yes* |
| Childhood Trauma Questionnaire – Short Form  (CTQ-SF) | Historical childhood trauma | Neglect (emotional, physical)   Abuse (emotional, sexual, physical) | Five scales | SR | 5-point Likert scale | 28 (25) | Bernstein et al., (2003). Development and validation of a brief screening version of the Childhood Trauma Questionnaire. *Child Abuse Negl. 2003 Feb;27(2):169-90* | Total/Cut-off value = All items  Subscale/Cut-off= Each subscale | Yes |
| Childhood Experience of Care and Abuse Instrument  (CECA-Q) | Adverse childhood experiences | Abuse (physical, sexual)  Lack of parental care (antipathy and neglect) | Three scales | SR | Yes/No | 50 | Smith et al, (2002). Childhood Experience of Care and Abuse Questionnaire (CECA.Q). Validation of a screening instrument for childhood adversity in clinical population. *Soc Psychiatry Psychiatr Epidemiol. 2002* | Subscale = Items for each subscale | Yes |
| Maltreatment and Abuse Chronology of Exposure  (MACE)  Three different versions (MAES, MACE, MACE-X) | Traumatic life Experiences | Abuse (physical verbal, physical maltreatment, non-verbal emotional, verbal, sexual)  Neglect (physical, emotional)  Bullying (peer physical, peer emotional)  Witnessing interparental violence and witnessing violence to siblings. | Ten scales | SR/IN | Yes/No | MACE= 52  MACE-X=75 | Teicher et al., (2015). The ‘Maltreatment and Abuse Chronology of Exposure’ (MACE) Scale for the Retrospective Assessment of Abuse and Neglect During Development. *PLoS One. 2015 Feb 25;10(2)* | - Overall adversity score (0-100) - Severity scores for each kind of adversity - Overall precence/abscence of adversity. - Information on timing and duration of exposure | Validation study not found |
| Parental rejection (PARQ) | Traumatic experiences | Parental rejection (affection, hostility, aggression, neglect, undifferentiated) | Four scales | SR | 4-point Likert scale | 60 | Rohner et al (2016) *Introduction to interpersonal acceptance–rejection theory (IPAR Theory), methods, evidence, and implications* | Total score | Validation study not found |
| The Adverse Childhood Experiences Scale (ACE) | Range of traumatic experiences | Abuse (psychological, physical, sexual)  Household dysfunction (substance, mental illness, mother treated violently, criminal) | Three scales | SR | Yes/No? | 17 | Felitti et al (1998). Relationship of childhood abuse and household dysfunction to many of the leading  causes of death in adults. *American Journal of Preventive Medicine, 14(4), 245–258.* | Total score from 0 (Unexposed) to 10 (Exposed all) | Validation study not found |
| Stressful life events – Questionnaire/ Adverse life events screening questionnaire (SLESQ/ALE)  Use of SLESQ and ALE are referred to the same validation paper | Lifetime trauma | Accidents or illnesses  Violent crime or victimization  Abuse (physical, emotional, sexual) | ? | SR | Yes/No | 13 | Goodman et al (1998). Assessing traumatic event exposure: General issues and preliminary findings for the stressful life events screening questionnaire. *Journal of TraumaticStress,11(3), 521–542* | Summing the total number of events. | Yes*  Allen et al (2015). Reliability and validity of the stressfull life events questionnaire among inpatiens with severe neuropsychiatric illness. |
| Traumatic life events – Questionnaire (TLEQ) | Life history of abuse | Abuse (physical, psychological, sexual)  Family violence  Neglect, Natural  Disaster/Accident/War/Illness | Five scales? | SR | Yes/No | 22 | Kubany et al (2001) Traumatic Life Events Questionnaire. Test and Instructions Manual (2nd draft). *Los Angeles (CA): Western Psychological Services* | Total score from 0 to 5. | Validation study not found |
| Distressing events (DEQ) | Distressing events that evaluate the presence of PTSD symptoms | PTSD symptoms | ? | SR | Yes/No | 17 | Kubany (2001). Distressing Event Questionnaire. Test and Instructions Manual (2nd draft). *Los Angeles (CA): Western Psychological Services* | Total score | Validation study not found |
| Childhood Victimization/bullying (DIAS-M) | Retrospective measure of childhood victimisation | Victimisation (physical, verbal, indirect) | Three scales | SR | 5-point Likert scale | 18 | Owens et al (2005). Sex and age differences in victimisation and conflict resolution among adolescents in a south Australian school. *Aggressive Behavior, 31, 1-12*. | Total score for each subscale. | Validation study not found |
| Childhood Traumatic Events Scale  (CTES) | Traumatic experiences | Death (family, friend)  Abuse (sexual, physical)  Parental divorce  Accidents/Sickness | ? | SR | 7-point Likert scale | ? | Pennebake et al (1988). Disclosure of traumas and psychosomatic processes. *Social science & medicine*, *26*(3), 327-332. | Sum score = 0-42 | Validation study not found |
| Trauma and Life events checklist (TALE) | Lifelong trauma screening | Abuse (physical, emotional, sexual)  Neglect (physical, emotional)  Bullying  Separation or loss  Violence/war/accidents/illness  Psychosis-related trauma | One scale? | SR/IN | Yes/No | 20 | Carr et al (2018). The Trauma and life events (TALE) checklist: Development of a tool for improving routine screening in people with psychosis. *Eur J Psychotraumatol. 2018 Sep 11;9(1):1512265* | Total items  cumulative items | Yes |
| The Trauma History Questionnaire  (THQ) | Trauma events | Crime related  General disaster  Physical or sexual experiences | ? | SR/IN | Yes/No | 24 | Hooper & Green (2011). Development, use, and psychometric properties of the Trauma History Questionnaire. Journal of Loss and Trauma, 16, 258-283. | No standard scoring method  Total score and sub score by number of types of events endorsed | Validation study not found |
| Trauma Screening Questionnaire  (TSQ) | PTSD symptomology | Traumatic and stressful events  PTSD symptoms | One scale | SR | Yes/No | 10 | Brewin et al (2002). Brief screening instrument for post-traumatic stress disorder. *Br. J. Psychiatry 181 (2), 158–162* | Total items | Yes |
| Trauma and distress scale  (TADS) | Traumatic life experiences | Abuse (physical, emotional, sexual)  Neglect (physical, emotional) | Five scales | SR | 5-point Likert scale | 43 | Patterson et al (2002) Trauma and Distress Scale, TADS—EPOS version 1.2. 2002 | Total score of the five domains. | Validation study not found |
| Childhood Experience of Care and Abuse interview  (CECA-I) | Adverse childhood experiences | Abuse and neglect  Separation, bullying and other | ? | IN | Yes/No | ? | Bifulc et al (1994). Childhood experience of care and abuse (CECA): A retrospective interview measure. *Journal of Child Psychology and psychiatry* | ? | Validation study not found |
| The Neglect Scale (NS) | Neglect | Multidimension report of neglectful behaviour by parents | Three scales | SR | 3-point Likert scale | 25 | Dubowitz et al (2011). Psycho-metric properties of a youth self-report measure of neglectful behaviour by parents. *Child Abuse & Neglect,35(6), 414–42* | Total score of each of the three scales | Validation study not found |
| Early Life Experience Scale  (ELES) | Threat and perceived subordination | Childhood memories of threat and perceived subordination | Three scales | SR | 5-point Likert scale | 15 | Gilbert et al (2003). Recall of threat and submissiveness in childhood: Development of a new scale and its relationship with depression, social comparison and shame. *Clinical Psychology & Psychotherapy, 10, 108–115* | Total score  Sub score | Validation study not found |
| The Victim Subscale of Cyber and Bullying Scale  (CVBS) | Cyber Bullying | Online cyber victimization | One scale | SR | 5-point Likert scale | 22 | Çetin et al (2011). Cyber victim and bullying scale: a study of validity and reliability. *Comput. Educ. 57, 2261–2271* | Total score  Sub score | Validation study not found |
| Multidimensional Peer Victimization Scale  (MPVS) | Evaluation of victimization | Victimization (physical, verbal)  Social manipulation  Attack on property | Four scales | SR | 3-point Likert scale | 16 | Mynard et al (2000). Development of the multidimensional peer-victimization scale. *Aggress. Behav. 26, 169–178* | Total score  Sub score | Validation study not found |
| Adverse Childhood Experiences International Questionnaire  (ACE-IQ) | Traumatic experiences | Abuse (physical, emotional, sexual)  Neglect (physical, emotional)  Household dysfunction (abandonment, substance-abuse, mentally ill, prison) | Tree scales | SR | Yes/No | 10 | Wingenfeld et al (2011). The reliable, valid and economic assessment of early traumatization: First psychometric characteristics of the German version of the Adverse Childhood Experiences Questionnaire (ACE). *Psychother Psychosom Med Psychol. 2011 Jan;61(1):e10-4. German* | Total scores range from 0–10 | Validation study not found |
| The Measure of Parenting Style (MOPS) | Parenting behaviours | Parental indifference, abuse, and over-control separately for mothers and fathers | Three scales | SR | 4-point Likert scale | 15 | Parker (1997) The development of a refined measure of dysfunctional parenting and assessment of its relevance in patients with affective disorders. *Psychol Med. 1997 Sep;27(5):1193-203* | Higher scores indicate more dysfunctional parenting | Validation study not found |
| Traumatic Experience Checklist  (TEC) | Childhood and adolescence trauma | Abuse  Neglect  Bodily threat and sexual harassment | Two scales | SR | Yes/No | 29 | Nijenhuis et al (2002). The psychometric characteristics of the Traumatic Experiences Questionnaire (TEC): First findings among psychiatric out-patients. *Clinical Psychology and Psychotherapy, 9, 200–210* | Total score ranges from 0 to 29. | Yes |
| Bully/Victim Questionnaire  (BVQ) | Measure forms of bullying. | Physical, verbal, or relational bullying | ? | SR | 5-point Likert scale? | 40 | Gothwal et al (2013). Revised Olweus Bully/Victim Questionnaire:evaluation in visually impaired. *Official Publication of the American Academy of Optometry90, 828–835*  Olweus D. The Revised Olweus Bully/Victim Questionnaire. Bergen, Norway: *Research Center for Health Promotion, University of Bergen; 1996*. | Total score | Validation study not found |
| Early Trauma Inventory  (ETI) | Trauma | General trauma  Physical and emotional trauma  Sexual abuse | Four scales | SR/IN | Yes/No | 56/62? | Bremner et al (2007). Psychometric properties of the early trauma inventory—Self report. *J. Nerv. Ment. Dis. 195(3), 211 (2007).* | Total score  Subscale score | Validation study not found |
| Childhood Life Events Questionnaire  (CATS) | Traumatic experiences | Abuse (sexual, physical, emotional)  Neglect  Degree of negativity | Three scales | SR | 5-point Likert scale | 38 | Kent & Waller (1998). The impact of childhood emotional abuse: An extension of the Child Abuse and Trauma Scale. *Child Abuse & Neglect,22(5), 393–399.* | Total and subscale summed scores | Validation study not found |
| Childhoold Life Events Questionnaire (CLEQ/CLES) | Childhood adversities | Death (parent, sibling, friend)  Parental separation/divorce/imprisonment, hospitalization, and suspension form work | ? | In | Yes/No | 9/13? | Upthegrove et al (2015) Adverse childhood events and psychosis in bipolar affective disorder. *Br. J. Psychiatry 206, 191–197* | Total score? | Validation study not found |
| Interview For Traumatic Events in Childhood  (ITEC) | Types of childhood maltreatment | Abuse (sexual, physical, emotional)  Neglect (physical) | Two scales | In | Yes/No  Follow up | 33/41 | Lobbestael et al (2009). Development and psychometric evaluation of a new assessment method for childhood maltreatment experiences: the interview for traumatic events in childhood (ITEC). *Child Abuse Negl. 2009 Aug;33(8):505-17.* | Sum score of abuse/neglect | Validation study not found |
| Juvenile Victimization Questionnaire  (JVQ-R2) | Range of adverse experiences | Crime and maltreatment  Peer and sibling victimization  Sexual victimization and witnessing other exposure to violence | ? | SR/IN | Yes/No Likert scale? | 45? | Flinkelhor et al (2011). The juvenile victimization questionnaire: 2nd revision (JVQ-R2). *Durham, NH: Crimes Against Children Research Center.* | Sum scores  Subscale scores | Validation study not found |
| Life Stressor Checklist  (LSC-R) | Childhood adversities | Victimization (physical and sexual)  Witnessing violence or interpersonal violence  Natural disaster or death of relative | ? | SR | Yes/No  5-point Likert scale | 30 | Wolfe et al (1997). The Life Stressor Checklist-Revised (LSC-R) | Overall life stressor score: 0-30.  Person’s endorsed life stressors (0-150) | Validation study not found |
| Brief Betrayal Trauma Survey  (BBTS) | Childhood exposure | Abuse (physical, sexual, emotional)  Witness of suicide/death/severe injury | Two scales? | SR | 3-point Likert | 12 | Goldberg & Freyd, (2006). Self-reports of potentially traumatic experiences in an adult community sample: Gender differences andtest-retest stabilities of the items in a brief betrayal-trauma survey. *Journal of Trauma & Dissociation,7(3), 39–63* | Total score before 18/After 18  Subscale score before 18/After 18 | Validation study not found |
| Florence Psychiatric Interview  (FPI) | Psychopathological state | Largest possible amount of information relevant to the psychopathological state of an individual | ? | IN | Yes/No  Likert scale | ? | Faravelli et al (2021). The Florence Psychiatric Interview. *Int JMethod Psych. 10:157–171.* | ? | Validation study not found |
| The Parent-Child Relationship Scale (PCRS) | Parent-child relationship | Positive and negative aspects of the parent-child relationship | Two scales? | SR | 5-point Likert scale | 15? | Pianta RC. (1992) *Child-Parent Relationship Scale.* University of Virginia. | Sum scores? | Validation study not found |
| Lifetime Incidence of Traumatic Events-Child  (LITE-C) | Loss and traumatic experiences in children | Accident or death of a family member  Exposure to threats or witness to violence  Sexual assault and many other potentially upsetting events | ? | SR | Yes/No | 16 | Greenwald & Rubin (1999). Assessment of posttraumatic symptoms in children:development and preliminary validation of parent and child scales. *Res SocWork Pract(1999) 9:61–75*. | Total score | Validation study not found |
| Posttraumatic Stress Disorder Checklist Spesific  (PCL-5) | Current PTSD symptomology | Trauma and PTSD Symptoms | One scale | SR | Yes/No | 20 | Weathers et al (2022). The PTSD Checklist for DSM-5 (PCL-5). *National Center for PTSD.* | Total=Sum of all items | Yes |
| Traumatic Events Screening Inventory for Children  (TESI-C) | Variety of traumatic events | Accident and natural disasters  Abuse (verbal, physical, sexual  Violence (domestic, community) | ? | IN | Yes/No/Not Sure | 16? | Ford and Rogers (1997)?  Ford, J. D., & Rogers, K. (1997, November). Empirically-based assessment of trauma and PTSD with children and adolescents. Paper presented at the annual convention of the International Society for Traumatic *Stress Studies, Montreal, Quebec, Canada.* | Clinical summary? | Validation study not found |
| The Trauma Symptom Checklist for Children (TSCC-A) | Traumatic events | Potential self-injury or suicidal tendency  Desire to harm Others or involvement in Fights  Expectations of sexual Maltreatment,  Fear of men or fear of women, fear of being killed | Five scales? | SR | 4-point Likert scale | 44 | Briere, J. (2011). Trauma Symptom Checklist for Children–A (TSCC-A) Italian Version. *Trento: Erickson Ed* | Summed scores | Validation study not found |
| Childhood Adversity Questionnaire  (CAQ) | Traumatic events | Lack of parental affection, parental factors, parental substance abuse  Household conflict, parental divorce, or separation  Abuse and neglect, parental indifference | ? | SR | Yes/No? | 20 | Rosenman & Rodgers (2004). Childhood adversity in an Australian population. *Social Psychiatry and Psychiatric Epidemiology 2004;39:695e702.* | Summed scores? | Validation study not found |
| Retrospective Bullying Questionnaire  (RBQ) | Forms of bullying | Verbal, physical, and indirect bullying | Three scales? | SR | 5-point Likert scale | 44 | Schafer et al (2004). Lonely in the crowd: Recollections of bullying. *British Journal of Developmental Psychology 22, 379-394* | Highest frequency and highest severity scores? | Validation study not found |
| History of Physical and Sexual Abuse Questionnaire  (HPSQ) | Trauma of abuse | Abuse (physical, sexual) | ? | SR | 4-point Likert scale | 14 | Meyer et al (1996). Reliability and validity of a measure of sexual and physical abuse histories among women with serious mental illness. *Child Abuse & Neglect, 20(3), 213–219* | Total score | Validation study not found |
| Recalled Parental Care Questionnaire (FEE) | Parental care | Perceived parental  rearing behaviour separately for the father and mother | ? | ? | ? | ? | Schumacker et al (1999). Der Fragebogen zum erinnerten elterlichen Erziehungsverhalten (FEE) [Retrospect on the parents: The questionnaire on the recalled parental rearing behavior *(FEE)] (GERMAN)* | ? | Validation study not found |
| The Sexual Abuse Questionnaire  (SAQ-2) | Sexual abuse | Traumatic experience of sexual abuse | One scale | SR | Yes/No | 36 | Lock et al (2005). The sexual abuse questionnaire: A preliminary examination of a time and cost efficient method in evaluating the presence of childhood sexual abuse in adult patients. *Journal of Child Sexual Abuse 14(1): 1–26.* | Cut of scores | Validation study not found |
| Brief Life events Questionnaire  (LTE-Q) | Life traumatic events | Serious injury or illness in oneself or a close relative  Death of parent/child/partner, death  of a family member, death of a friend  Separation from a partner, loss of job or financial difficulties | ? | SR | Yes/No | 20 | Brugha et al (1985). The List of Threatening Experiences: a subset of 12 life event categories with considerable long-term contextual threat. *Psychol Med. 1985;15(1):189–194* | Total score from 0-20 | Validation study not found |
| The Chinese Adolescent Self Rating Life Event Checklist  (ASLEC) | Measure of negative life events | Interpersonal difficulties  Academic pressure  Being punished, personal loss, health, and adaptability | Five scales | SR | 5-point Likert scale | 27 | Liu et al (1997). Reliability and validity of the adolescents self-rating life events checklist. *Chinese Journal of Clinical Psychology, 5(1), 34–36.* | Total score? | Validation study not found |
| The Havard Trauma Questionnaire  (HTQ) | Different versions of the questionnaire |  | ? | SR | ? | ? | The Harvard Trauma Questionnaire (HTQ) is a checklist written by HPRT. | ? | Validation study not found |
| Conflict Tactic Scale (CTS) | Intra-family violence | Physical assault scale  Aggression scale  Negotiation scale  Injury scale and sexual coercion scale | Five scales | SR | Yes/No | 39 | Strauss (1979). Measuring intrafamily Conflict and Violence. *National Council on Family Relations* | No total provided  Subscales = Items for each subscale | Yes |
| Adverse Social Experience Questionnaire (ASEQ) | List of traumatic social situations | Abuse (physical, emotional, sexual)  Household dysfunction (substance use, violence) | Two scales | SR | Yes/No | 22 | Felitti et al (1998). | Overall score (0-22/0-44) | Validation study not found |
| Computerized assessment instrument (PEERS) | Computerized peer nominations assessment of common forms of bullying | Bullying (physical, verbal, material and relational). | Four scales | SR | ? | ? | Verlinden et al (2014). Detecting bullying in early elementary school with a computerized peer-nomination instrument. *Psychol Assess. 2014;26(2):628.* | Cut of scores? | Validation study not found |
| The Child Abuse Self‑Report Scale (CASRS) | Childhood adversities | Psychological abuse  Parental neglect  Physical maltreatment and sexual trauma. | Four scales | SR | 4-point Likert scale | 38 | Mohammadkha et al (2003). Development, validation and reliability of child abuse self report scale. *Medical Journal of the Islamic Republic of Iran* | Sum of scores | Validation study not found |
| International trauma exposure measure (ITEM) | ? |  | ? | ? | ? | ? | ? | ? | ? |
| Bully Survey Chinese version (C-BSA) | Bullying | Screening for subjective experiences of being bullied, personal consequences of bullying and experience of acting as a bully perpetrator | Three scales | SR | 5-point Likert scale | 21? | Haidl et al (2020). Validation of the bullying scale for adults - results of the PRONIA-study. *J Psychiatr Res* | Sum scores | Validation study not found |

Note. ? = cannot retrieve original validation paper. * = validation study identified by independent search or reference list.

**Table 3.** Refence list of included studies

1. Ajnakina O, Trotta A, Di Forti M, et al. Different types of childhood adversity and 5-year outcomes in a longitudinal cohort of first-episode psychosis patients. *Psychiatry Research*. 269:199-206. doi:doi:

2. Ajnakina O, Trotta A, Oakley-Hannibal E, et al. Impact of childhood adversities on specific symptom dimensions in first-episode psychosis. *Psychological Medicine*. 46(2):317-326. doi:doi:

3. Akbey ZY, Yildiz M, Gunduz N. Is There Any Association Between Childhood Traumatic Experiences, Dissociation and Psychotic Symptoms in Schziophrenic Patients? *Psychiatry Investigation*. 16(5):346-354. doi:doi:

4. Akun E, Batigun AD. Negative symptoms and recollections of parental rejection: The moderating roles of psychological maladjustment and gender. *Psychiatry Research*. 275:332-337. doi:doi:

5. Akun E, Batigun AD, Ozguven HD, Baskak B. Positive symptoms and perceived parental acceptance-rejection in childhood: The moderating roles of socioeconomic status and gender. *Turk Psikiyatri Dergisi*. 29(2):1-7. doi:doi:

6. Alemany S, Arias B, Aguilera M, et al. Childhood abuse, the BDNF-Val66Met polymorphism and adult psychotic-like experiences. *The British Journal of Psychiatry*. 199(1):38-42. doi:doi:

7. Alemany S, Arias B, Fatjo-Vilas M, et al. Psychosis-inducing effects of cannabis are related to both childhood abuse and COMT genotypes. *Acta Psychiatrica Scandinavica*. 129(1):54-62. doi:doi:

8. Alemany S, Ayesa-Arriola R, Arias B, et al. Childhood abuse in the etiological continuum underlying psychosis from first-episode psychosis to psychotic experiences. *European Psychiatry*. 30(1):38-42. doi:doi:

9. Alvarez M-J, Masramom H, Foguet-Boreu Q, et al. Childhood trauma in schizophrenia spectrum disorders: Dissociative, psychotic symptoms, and suicide behavior. *Journal of Nervous and Mental Disease*. 209(1):40-48. doi:doi:

10. Alvarez M-J, Masramon H, Pena C, et al. Cumulative effects of childhood traumas: Polytraumatization, dissociation, and schizophrenia. *Community Mental Health Journal*. 51(1):54-62. doi:doi:

11. Alvarez M-J, Roura P, Oses A, Foguet Q, Sola J, Arrufat F-X. Prevalence and clinical impact of childhood trauma in patients with severe mental disorders. *Journal of Nervous and Mental Disease*. 199(3):156-161. doi:doi:

12. Anilmis J, Stewart C, Roddy S, et al. Understanding the relationship between schematic beliefs, bullying, and unusual experiences in 8-14year olds. *European Psychiatry*. 30(8):920-923. doi:doi:

13. Appiah-Kusi E, Fisher H, Petros N, et al. Do cognitive schema mediate the association between childhood trauma and being at ultra-high risk for psychosis? *Journal of Psychiatric Research*. 88:89-96. doi:doi:

14. Appiah-Kusi E, Wilson R, Colizzi M, et al. Childhood trauma and being at-risk for psychosis are associated with higher peripheral endocannabinoids. *Psychological Medicine*. 50(11):1862-1871. doi:doi:

15. Arranz S, Monferrer N, Jose Algora M, et al. The relationship between the level of exposure to stress factors and cannabis in recent onset psychosis. *Schizophrenia Research*. 201:352-359. doi:doi:

16. Ashcroft K, Kingdon DG, Chadwick P. Persecutory delusions and childhood emotional abuse in people with a diagnosis of schizophrenia. *Psychosis: Psychological, Social and Integrative Approaches*. 4(2):168-171. doi:doi:

17. Ashford CD, Ashcroft K, Maguire N. Emotions, traits and negative beliefs as possible mediators in the relationship between childhood experiences of being bullied and paranoid thinking in a non-clinical sample. *Journal of Experimental Psychopathology*. 2012 2012;3(4):624-638. doi:doi:

18. Asmal L, Kilian S, du Plessis S, et al. Childhood trauma associated white matter abnormalities in first-episode schizophrenia. *Schizophrenia Bulletin*. 45(2):369-376. doi:doi:

19. Ayesa-Arriola R, Setien-Suero E, Marques-Feixa L, et al. The synergetic effect of childhood trauma and recent stressful events in psychosis: Associated neurocognitive dysfunction. *Acta Psychiatrica Scandinavica*. 141(1):43-51. doi:doi:

20. Badcock JC, Br, R, Thomas N, Hayward M, Paulik G. Multimodal versus unimodal auditory hallucinations in clinical practice: Clinical characteristics and treatment outcomes. *Psychiatry Research*. 297:7. doi:doi:

21. Barnes GL, Emsley R, Garety P, Hardy A. Identifying victimisation profiles in people with psychosis and a history of childhood trauma: a latent class analysis. *Psychosis-Psychological Social and Integrative Approaches*.11. doi:doi:

22. Barnes GL, Emsley R, Garety P, Hardy A. Investigating Specific Associations Between Childhood Victimization Profiles and Positive Psychosis Symptoms: The Mediating Roles of Anxiety, Depression, and Schema. *Schizophrenia Bulletin Open*. 2023-1 2023;4(1):sgad017. doi:doi:https://dx.doi.org/10.1093/schizbullopen/sgad017

23. Barry TJ, Villanueva-Romero CM, Hern, ez-Viadel JV, Ricarte JJ. Early life adversity and the specificity of autobiographical memory amongst people with schizophrenia. *Behaviour Research and Therapy Vol 140 2021, ArtID 103836*. 140doi:doi:

24. Bartels-Velthuis A, Wigman J, Jenner J, Bruggeman R, van Os J. Course of auditory vocal hallucinations in childhood: 11-year follow-up study. *Acta Psychiatrica Scandinavica*. 134(1):6-15. doi:doi:

25. Baryshnikov I, Aaltonen K, Suvisaari J, et al. Features of borderline personality disorder as a mediator of the relation between childhood traumatic experiences and psychosis-like experiences in patients with mood disorder. *European Psychiatry*. 49:9-15. doi:doi:

26. Baudin G, Godin O, Lajnef M, et al. Differential effects of childhood trauma and cannabis use disorders in patients suffering from schizophrenia. *Schizophrenia Research*. 175(1):161-167. doi:doi:

27. Baudin G, Szoke A, Richard J-R, Pelissolo A, Leboyer M, Schurhoff F. Childhood trauma and psychosis: Beyond the association. *Child Abuse & Neglect*. 72:227-235. doi:doi:

28. Baygin C, Sevincok L, Dogan B, Sevincok D, Sair YB. The mediation role of schizotypal traits in the relationship between childhood trauma and earlier onset of panic disorder. *Psychosis*. 2021 2021;doi:doi:

29. Beasley RE, Kivisto AJ, Leonhardt BL, Waldron JS. Childhood Maltreatment and Psychosis: A Comparative Validity Study of Maltreatment Indices. *Child Maltreatment*. 26(2):228-237. doi:doi:

30. Begemann MJH, Sommer IE, Br, et al. Auditory verbal hallucinations and childhood trauma subtypes across the psychosis continuum: a cluster analysis. *Cognitive Neuropsychiatry*.1-19. doi:doi:

31. Bellido-Zanin G, Perona-Garcelan S, Senin-Calderon C, Lopez-Jimenez AM, Ruiz-Veguilla M, Rodriguez-Testal JF. Childhood memories of threatening experiences and submissiveness and its relationship to hallucination proneness and ideas of reference: The mediating role of dissociation. *Scandinavian Journal of Psychology*. 59(4):407-413. doi:doi:

32. Bendall S, Eastwood O, Spelman T, et al. Childhood trauma is prevalent and associated with co-occurring depression, anxiety, mania and psychosis in young people attending Australian youth mental health services. *Australian and New Zealand Journal of Psychiatry*. 2023-5-26 2023:48674231177223. doi:doi:https://dx.doi.org/10.1177/00048674231177223

33. Bendall S, Hulbert CA, Alvarez-Jimenez M, Allott K, McGorry PD, Jackson HJ. Testing a model of the relationship between childhood sexual abuse and psychosis in a first-episode psychosis group: The role of hallucinations and delusions, posttraumatic intrusions, and selective attention. *Journal of Nervous and Mental Disease*. 201(11):941-947. doi:doi:

34. Berg A, Aas M, Larsson S, et al. Childhood trauma mediates the association between ethnic minority status and more severe hallucinations in psychotic disorder. *Psychological Medicine*. 45(1):133-142. doi:doi:

35. Berry K, Fleming P, Wong S, Bucci S, ra. Associations between trauma, dissociation, adult attachment and proneness to hallucinations. *Behavioural and Cognitive Psychotherapy*. 46(3):292-301. doi:doi:

36. Bi XJ, Hu L, Qiao DD, et al. Evidence for an Interaction Between NEDD4 and Childhood Trauma on Clinical Characters of Schizophrenia With Family History of Psychosis. *Frontiers in psychiatry Frontiers Research Foundation*. 2021 2021;12:608231. doi:doi:

37. Bi XJ, Lv XM, Ai XY, et al. Childhood trauma interacted with BDNF Val66Met influence schizophrenic symptoms. *Medicine*. 97(13):e0160. doi:doi:

38. Bird JC, Waite F, Rowsell E, Fergusson EC, Freeman D. Cognitive, affective, and social factors maintaining paranoia in adolescents with mental health problems: A longitudinal study. *Psychiatry Research*. 257:34-39. doi:doi:

39. Blom JD, Mangoenkarso E. Sexual hallucinations in schizophrenia spectrum disorders and their relation with childhood trauma. *Frontiers in Psychiatry Vol 9 2018, ArtID 193*. 9doi:doi:

40. Blose BA, Godleski SA, Houston RJ, Schenkel LS. The Indirect Effect of Peritraumatic Dissociation on the Relationship Between Childhood Maltreatment and Schizotypy. *Journal of Interpersonal Violence*. 2023-11-3 2023;38(5):5282-5304. doi:doi:https://dx.doi.org/10.1177/08862605221122832

41. Bolhuis K, Steenkamp LR, Tiemeier H, et al. A Prospective Cohort Study on the Intergenerational Transmission of Childhood Adversity and Subsequent Risk of Psychotic Experiences in Adolescence. *Schizophrenia Bulletin*. 2023-11-5 2023;49(3):799-808. doi:doi:https://dx.doi.org/10.1093/schbul/sbac195

42. Boonmann C, Grisso T, Guy LS, et al. Childhood traumatic experiences and mental health problems in sexually offending and non-sexually offending juveniles. *Child and Adolescent Psychiatry and Mental Health Vol 10 2016, ArtID 45*. 10doi:doi:

43. Bortolon C, Raffard S. Dissociation mediates the relationship between childhood trauma and experiences of seeing visions in a French sample. *Journal of Nervous and Mental Disease*. 206(11):850-858. doi:doi:

44. Bortolon C, Raffard S. Affective and cognitive factors associated with hallucination proneness in the general population: The role of shame and trauma-related intrusions. *Cognitive Neuropsychiatry*. 24(6):406-420. doi:doi:

45. Bortolon C, Seille J, Raffard S. Exploration of trauma, dissociation, maladaptive schemas and auditory hallucinations in a French sample. *Cognitive Neuropsychiatry*. 22(6):468-485. doi:doi:

46. Boyda D, McFeeters D, Dhingra K, Rhoden L. Childhood maltreatment and psychotic experiences: Exploring the specificity of early maladaptive schemas. *Journal of Clinical Psychology*. 74(12):2287-2301. doi:doi:

47. Braehler C, Valiquette L, Holowka D, et al. Childhood trauma and dissociation in first-episode psychosis, chronic schizophrenia and community controls. *Psychiatry Research*. 210(1):36-42. doi:doi:

48. Brown P, Waite F, Freeman D. Parenting behaviour and paranoia: a network analysis and results from the National Comorbidity Survey-Adolescents (NCS-A). *Social Psychiatry & Psychiatric Epidemiology*. 56(4):593-604. doi:doi:

49. Bruni A, Carbone EA, Pugliese V, et al. Childhood adversities are different in Schizophrenic Spectrum Disorders, Bipolar Disorder and Major Depressive Disorder. *BMC Psychiatry Vol 18 2018, ArtID 391*. 18doi:doi:

50. Cakir S, Durak RT, Ozyildirim I, Ince E, Sar V. Childhood trauma and treatment outcome in bipolar disorder. *Journal of Trauma & Dissociation*. 17(4):397-409. doi:doi:

51. Campbell C, Barrett S, Shannon C, et al. The relationship between childhood trauma and neuropsychological functioning in first episode psychosis. *Psychosis: Psychological, Social and Integrative Approaches*. 5(1):48-59. doi:doi:

52. Cancel A, Comte M, Truillet R, et al. Childhood neglect predicts disorganization in schizophrenia through grey matter decrease in dorsolateral prefrontal cortex. *Acta Psychiatrica Scandinavica*. 132(4):244-256. doi:doi:

53. Capuzzi E, Caldiroli A, Besana F, Cova F, Buoli M, Clerici M. Factors associated with psychotic symptoms among a sample of male prisoners with substance use disorder: A cross-sectional study. *Journal of Substance Abuse Treatment*. 118:108104. doi:doi:

54. Carbone EA, Pugliese V, Bruni A, et al. Adverse childhood experiences and clinical severity in bipolar disorder and schizophrenia: A transdiagnostic two-step cluster analysis. *Journal of Affective Disorders*. 259:104-111. doi:doi:

55. Carlyle M, Constable T, Walter ZC, et al. Cannabis-induced dysphoria/paranoia mediates the link between childhood trauma and psychotic-like experiences in young cannabis users. *Schizophrenia Research*. 238:178-184. doi:doi:

56. Carrillo de Albornoz CM, Gutierrez B, Ibanez-Casas I, Cervilla JA. Paranoia and Suicidality: A Cross-Sectional Study in the General Population. *Archives of Suicide Research*.1-13. doi:doi:

57. Carvalho CB, da Motta C, Pinto-Gouveia J, Peixoto E. Psychosocial roots of paranoid ideation: The role of childhood experiences, social comparison, submission, and shame. *Clinical Psychology & Psychotherapy*. 25(5):650-661. doi:doi:

58. Carvalho CB, Motta C, Pinto-Gouveia J, Peixoto E. Influence of family and childhood memories in the development and manifestation of paranoid ideation. *Clinical Psychology & Psychotherapy*. 23(5):397-406. doi:doi:

59. Catalan A, Angosto V, Diaz A, et al. Relation between psychotic symptoms, parental care and childhood trauma in severe mental disorders. *Psychiatry Research*. 251:78-84. doi:doi:

60. Catalan A, Diaz A, Angosto V, et al. Can childhood trauma influence facial emotion recognition independently from a diagnosis of severe mental disorder? *Revista De Psiquiatria Y Salud Mental*. 13(3):140-149. doi:doi:

61. Catone G, Gritti A, Russo K, et al. Details of the Contents of Paranoid Thoughts in Help-Seeking Adolescents with Psychotic-Like Experiences and Continuity with Bullying and Victimization: A Pilot Study. *Behavioral sciences*. 10(8):29. doi:doi:

62. Catone G, Marotta R, Pisano S, et al. Psychotic-like experiences in help-seeking adolescents: Dimensional exploration and association with different forms of bullying victimization-A developmental social psychiatry perspective. *International Journal of Social Psychiatry*. 63(8):752-762. doi:doi:

63. Chae S, Sim M, Lim M, Na J, Kim D. Multivariate Analysis of Relationship between Childhood Trauma and Psychotic Symptoms in Patients with Schizophrenia. *Psychiatry Investigation*. 12(3):397-401. doi:doi:

64. Chase KA, Melbourne JK, Rosen C, et al. Traumagenics: At the intersect of childhood trauma, immunity and psychosis. *Psychiatry Research*. 273:369-377. doi:doi:

65. Chatziioannidis S, Andreou C, Agorastos A, et al. The role of attachment anxiety in the relationship between childhood trauma and schizophrenia-spectrum psychosis. *Psychiatry Research*. 276:223-231. doi:doi:

66. Chen LH, Toulopoulou T. Pathways linking school bullying and psychotic experiences: Multiple mediation analysis in Chinese adolescents and young adults. *Frontiers in Psychiatry*. 2022-10 2022;13:11. doi:doi:10.3389/fpsyt.2022.1007348

67. Cho Y, Kim D, Kim SH. Prevalence and clinical correlates of childhood trauma among inpatients diagnosed with bipolar disorder: a matched comparison with schizophrenia. *Psychosis*. 2021 2021;13(1):13-23. doi:doi:

68. Choi JY. Posttraumatic stress symptoms and dissociation between childhood trauma and two different types of psychosis-like experience. *Child Abuse & Neglect*. 72:404-410. doi:doi:

69. Choi JY, Choi YM, Kim B, Lee DW, Gim MS, Park SH. The effects of childhood abuse on self-reported psychotic symptoms in severe mental illness: Mediating effects of posttraumatic stress symptoms. *Psychiatry Research*. 229(1):389-393. doi:doi:

70. Chung YC, Yun JY, Nguyen TB, et al. Network analysis of trauma in patients with early-stage psychosis. *Scientific Reports*. 11(1):22749. doi:doi:

71. Clemmensen L, van Os J, Drukker M, et al. Psychotic experiences and hyper-theory-of-mind in preadolescence-A birth cohort study. *Psychological Medicine*. 46(1):87-101. doi:doi:

72. Cole CL, Newman-Taylor K, Kennedy F. Dissociation mediates the relationship between childhood maltreatment and subclinical psychosis. *Journal of Trauma & Dissociation*. 17(5):577-592. doi:doi:

73. Collip D, Myin-Germeys I, Wichers M, et al. FKBP5 as a possible moderator of the psychosis-inducing effects of childhood trauma. *The British Journal of Psychiatry*. 202(4):261-268. doi:doi:

74. Comacchio C, Howard LM, Bonetto C, et al. The impact of gender and childhood abuse on age of psychosis onset, psychopathology and needs for care in psychosis patients. *Schizophrenia Research*. 2019 2019;210:164-171. doi:doi:

75. Corcoran M, Hawkins EL, O'Hora D, et al. Are working memory and glutamate concentrations involved in early-life stress and severity of psychosis? *Brain and Behavior*. 10(6):e01616. doi:doi:

76. Corsi-Zuelli F, Loureiro CM, Shuhama R, et al. Cytokine profile in first-episode psychosis, unaffected siblings and community-based controls: The effects of familial liability and childhood maltreatment. *Psychological Medicine*. 50(7):1139-1147. doi:doi:

77. Crabtree J, Hudson JL, Newton-John T. Anxiety and Adverse Life Events in Professional Creative and Early Psychosis Populations. *Psychiatry*. 2020 2020;83(4):328-343. doi:doi:

78. Cristobal-Narvaez P, Sheinbaum T, Ballespi S, et al. Impact of Adverse Childhood Experiences on Psychotic-Like Symptoms and Stress Reactivity in Daily Life in Nonclinical Young Adults. *PLoS ONE [Electronic Resource]*. 2016 2016;11(4):e0153557. doi:doi:

79. Cristobal-Narvaez P, Sheinbaum T, Myin-Germeys I, et al. The role of stress-regulation genes in moderating the association of stress and daily-life psychotic experiences. *Acta Psychiatrica Scandinavica*. 136(4):389-399. doi:doi:

80. Cristobal-Narvaez P, Sheinbaum T, Rosa A, et al. The interaction between childhood bullying and the FKBP5 gene on psychotic-like experiences and stress reactivity in real life. *PLoS ONE Vol 11(7), 2016, ArtID e0158809*. 11(7)doi:doi:

81. Crush E, Arseneault L, Moffitt TE, et al. Protective factors for psychotic experiences amongst adolescents exposed to multiple forms of victimization. *Journal of Psychiatric Research*. 104:32-38. doi:doi:

82. Daemen M, van Amelsvoort T, Reininghaus U. Momentary Self-esteem as a Process Underlying the Association Between Childhood Trauma and Psychosis: Experience Sampling Study. *JMIR Mental Health*. 2023-11-4 2023;10:e34147. doi:doi:https://dx.doi.org/10.2196/34147

83. Dahoun T, Nour MM, McCutcheon RA, Adams RA, Bloomfield MAP, Howes OD. The relationship between childhood trauma, dopamine release and dexamphetamine-induced positive psychotic symptoms: a [<sup>11</sup>C]-(+)-PHNO PET study. *Transl Psychiatry Psychiatry*. 9(1):287. doi:doi:

84. Dantchev S, Zammit S, Wolke D. Sibling bullying in middle childhood and psychotic disorder at 18 years: A prospective cohort study. *Psychological Medicine*. 48(14):2321-2328. doi:doi:

85. Davis PE, Webster LA, Fernyhough C, Ralston K, Kola-Palmer S, Stain HJ. Adult report of childhood imaginary companions and adversity relates to concurrent prodromal psychosis symptoms. *Psychiatry Research*. 271:150-152. doi:doi:

86. de Castro-Catala M, Pena E, Kwapil TR, et al. Interaction between FKBP5 gene and childhood trauma on psychosis, depression and anxiety symptoms in a non-clinical sample. *Psychoneuroendocrinology*. 85:200-209. doi:doi:

87. de Castro-Catala M, van Nierop M, Barrantes-Vidal N, et al. Childhood trauma, BDNF Val66Met and subclinical psychotic experiences. Attempt at replication in two independent samples. *Journal of Psychiatric Research*. 83:121-129. doi:doi:

88. de Vos C, Thompson A, Amminger P, et al. The relationship between childhood trauma and clinical characteristics in ultra-high risk for psychosis youth. *Psychosis: Psychological, Social and Integrative Approaches*. 11(1):28-41. doi:doi:

89. DeCou CR, Lynch SM, DeHart DD, Belknap J. Evaluating childhood and adulthood victimization as predictors of psychotic disorders: Findings from a nationwide study of women in jail. *Psychosis: Psychological, Social and Integrative Approaches*. 9(3):282-285. doi:doi:

90. Degnan A, Berry K, Humphrey C, Bucci S. The role of attachment and dissociation in the relationship between childhood interpersonal trauma and negative symptoms in psychosis. *Clinical Psychology & Psychotherapy*. 25:25. doi:doi:

91. DeRosse P, Nitzburg GC, Kompancaril B, Malhotra AK. The relation between childhood maltreatment and psychosis in patients with schizophrenia and non-psychiatric controls. *Schizophrenia Research*. 155(1):66-71. doi:doi:

92. Dias A, Sales L, Hessen DJ, Kleber RJ. Child maltreatment and psychological symptoms in a Portuguese adult community sample: The harmful effects of emotional abuse. *European Child & Adolescent Psychiatry*. 24(7):767-778. doi:doi:

93. Dizinger JMB, Doll CM, Rosen M, et al. Does childhood trauma predict schizotypal traits? A path modelling approach in a cohort of help-seeking subjects. *European Archives of Psychiatry & Clinical Neuroscience*. 4:04. doi:doi:

94. Duhig M, Patterson S, Connell M, et al. The prevalence and correlates of childhood trauma in patients with early psychosis. *Australian and New Zealand Journal of Psychiatry*. 49(7):651-659. doi:doi:

95. Daalman K, Diederen K, Derks E, van Lutterveld R, Kahn R, Sommer IE. Childhood trauma and auditory verbal hallucinations. *Psychological Medicine*. 42(12):2475-2484. doi:doi:

96. Egerton A, Valmaggia LR, Howes OD, et al. Adversity in childhood linked to elevated striatal dopamine function in adulthood. *Schizophrenia Research*. 176(2):171-176. doi:doi:

97. Enthoven AD, Gangadin SS, de Haan L, et al. The association of childhood trauma with depressive and negative symptoms in recent onset psychosis: a sex-specific analysis. *Psychological Medicine*. 2023-7-12 2023:1-10. doi:doi:https://dx.doi.org/10.1017/S0033291723001824

98. Ered A, Ellman LM. Specificity of Childhood Trauma Type and Attenuated Positive Symptoms in a Non-Clinical Sample. *Journal of Clinical Medicine*. 8(10):25. doi:doi:

99. Etain B, Lajnef M, Bellivier F, et al. Revisiting the association between childhood trauma and psychosis in bipolar disorder: A quasi-dimensional path-analysis. *Journal of Psychiatric Research*. 84:73-79. doi:doi:

100. Evans GJ, Reid G, Preston P, Palmier-Claus J, Sellwood W. Trauma and psychosis: The mediating role of self-concept clarity and dissociation. *Psychiatry Research*. 228(3):626-632. doi:doi:

101. Faravelli C, Mansueto G, Palmieri S, et al. Childhood adversity, cortisol levels, and psychosis: A retrospective investigation. *Journal of Nervous and Mental Disease*. 205(7):574-579. doi:doi:

102. Fekih-Romdhane F, Tira S, Cheour M. Childhood sexual abuse as a potential predictor of psychotic like experiences in Tunisian college students. *Psychiatry Research*. 275:181-188. doi:doi:

103. Fekih‐Romdhane F, Nsibi T, Sassi H, Cheour M. Link between childhood trauma and psychotic‐like experiences in non‐affected siblings of schizophrenia patients: A case‐control study. *Early Intervention in Psychiatry*. 2021 2021;15(5):1154-1166. doi:doi:

104. Fisher H, Jones P, Fearon P, et al. The varying impact of type, timing and frequency of exposure to childhood adversity on its association with adult psychotic disorder. *Psychological Medicine*. 40(12):1967-1978. doi:doi:

105. Fisher HL, Appiah-Kusi E, Grant C. Anxiety and negative self-schemas mediate the association between childhood maltreatment and paranoia. *Psychiatry Research*. 196(2):323-324. doi:doi:

106. Fisher HL, Craig TK, Fearon P, et al. Reliability and comparability of psychosis patients' retrospective reports of childhood abuse. *Schizophrenia Bulletin*. 2011-5 2011;37(3):546-53. doi:doi:https://dx.doi.org/10.1093/schbul/sbp103

107. Fisher HL, Morgan C, Hutchinson G, et al. Childhood abuse and psychosis in different ethnic groups: A case-control study. *Psychiatry Research Journal*. 2010 2010;1(4):335-343. doi:doi:

108. Freitas EL, Loch A, Andrade r, et al. Childhood maltreatment in individuals at risk of psychosis: Results from the Brazilian SSAPP cohort. *International Journal of Social Psychiatry*. 66(6):566-575. doi:doi:

109. Frissen A, Lieverse R, Drukker M, van Winkel R, Delespaul P. Childhood trauma and childhood urbanicity in relation to psychotic disorder. *Social Psychiatry and Psychiatric Epidemiology: The International Journal for Research in Social and Genetic Epidemiology and Mental Health Services*. 50(10):1481-1488. doi:doi:

110. Frydecka D, Misiak B, Kotowicz K, et al. The interplay between childhood trauma, cognitive biases, and cannabis use on the risk of psychosis in nonclinical young adults in Poland. *European Psychiatry: the Journal of the Association of European Psychiatrists*. 63(1):e35. doi:doi:

111. Fung AL-C, Raine A. Peer victimization as a risk factor for schizotypal personality in childhood and adolescence. *Journal of Personality Disorders*. 26(3):428-434. doi:doi:

112. Gabinio T, Ricci T, Kahn JP, Malaspina D, Moreira H, Veras AB. Early trauma, attachment experiences and comorbidities in schizophrenia. *Trends in Psychiatry and Psychotherapy*. 40(3):179-184. doi:doi:

113. Galletly C, Clark L, McFarlane A, et al. Childhood lead exposure, childhood trauma, substance use and subclinical psychotic experiences-a longitudinal cohort study. *Psychiatry Research*. 239:54-61. doi:doi:

114. Garcia M, Montalvo I, Creus M, et al. Sex differences in the effect of childhood trauma on the clinical expression of early psychosis. *Comprehensive Psychiatry*. 68:86-96. doi:doi:

115. Gaudiano B, Zimmerman M. The relationship between childhood trauma history and the psychotic subtype of major depression. *Acta Psychiatrica Scandinavica*. 121(6):462-470. doi:doi:

116. Gaweda L, Goritz AS, Moritz S. Mediating role of aberrant salience and self-disturbances for the relationship between childhood trauma and psychotic-like experiences in the general population. *Schizophrenia Research*. 206:149-156. doi:doi:

117. Gaweda L, Pionke R, Hartmann J, Nelson B, Cechnicki A, Frydecka D. Toward a Complex Network of Risks for Psychosis: Combining Trauma, Cognitive Biases, Depression, and Psychotic-like Experiences on a Large Sample of Young Adults. *Schizophrenia Bulletin*. 47(2):395-404. doi:doi:

118. Gayer-Anderson C, Fisher HL, Fearon P, et al. Gender differences in the association between childhood physical and sexual abuse, social support and psychosis. *Social Psychiatry and Psychiatric Epidemiology: The International Journal for Research in Social and Genetic Epidemiology and Mental Health Services*. 50(10):1489-1500. doi:doi:

119. Gekker M, Freire Coutinho E, Silva r, et al. Early scars are forever: Childhood abuse in patients with adult-onset PTSD is associated with increased prevalence and severity of psychiatric comorbidity. *Psychiatry Research*. 267:1-6. doi:doi:

120. Gerges S, Haddad C, Daoud T, et al. A cross-sectional study of current and lifetime sexual hallucinations and delusions in lebanese patients with schizophrenia: Frequency, characterization, and association with childhood traumatic experiences and disease severity. *BMC Psychiatry Vol 22, 2022, ArtID 360*. 2022-5 2022;22doi:doi:https://dx.doi.org/10.1186/s12888-022-04012-z

121. Gizdic A, Sheinbaum T, Kwapil TR, Barrantes-Vidal N. Empirically-derived dimensions of childhood adversity and cumulative risk: associations with measures of depression, anxiety, and psychosis-spectrum psychopathology. *European Journal of Psychotraumatology*. 2023 2023;14(2):2222614. doi:doi:https://dx.doi.org/10.1080/20008066.2023.2222614

122. Goldstone E, Farhall J, Ong B. Synergistic pathways to delusions: Enduring vulnerabilities, proximal life stressors and maladaptive psychological coping. *Early Intervention in Psychiatry*. 5(2):122-131. doi:doi:

123. Goldstone E, Farhall J, Ong B. Modelling the emergence of hallucinations: Early acquired vulnerabilities, proximal life stressors and maladaptive psychological processes. *Social Psychiatry and Psychiatric Epidemiology: The International Journal for Research in Social and Genetic Epidemiology and Mental Health Services*. 47(9):1367-1380. doi:doi:

124. Gomez JM, Kaehler LA, Freyd JJ. Are hallucinations related to betrayal trauma exposure? A three-study exploration. *Psychological Trauma: Theory, Research, Practice, and Policy*. 6(6):675-682. doi:doi:

125. Goodall K, Rush R, Grunwald L, Darling S, Tiliopoulos N. Attachment as a partial mediator of the relationship between emotional abuse and schizotypy. *Psychiatry Research*. 230(2):531-536. doi:doi:

126. Green K, Webster A. The relationships between childhood abuse and neglect, sub-clinical symptoms of psychosis and self-harm in a non-clinical community sample. *Journal of Child & Adolescent Trauma*. 2022-9 2022;15(3):605-614. doi:doi:https://dx.doi.org/10.1007/s40653-021-00422-5

127. Gromann P, Goossens F, Olthof T, Pronk J, Krabbendam L. Self-perception but not peer reputation of bullying victimization is associated with non-clinical psychotic experiences in adolescents. *Psychological Medicine*. 43(4):781-787. doi:doi:

128. Guloksuz S, Pries L-K, Delespaul P, et al. Examining the independent and joint effects of molecular genetic liability and environmental exposures in schizophrenia: Results from the EUGEI study. *World Psychiatry*. 18(2):173-182. doi:doi:

129. Haidl TK, Hedderich DM, Rosen M, et al. The non-specific nature of mental health and structural brain outcomes following childhood trauma. *Psychological Medicine*.1-10. doi:doi:

130. Hardy A, Emsley R, Freeman D, et al. Psychological Mechanisms Mediating Effects Between Trauma and Psychotic Symptoms: The Role of Affect Regulation, Intrusive Trauma Memory, Beliefs, and Depression. *Schizophrenia Bulletin*. 42:S34-43. doi:doi:

131. Haug E, Oie M, Andreassen OA, et al. Anomalous self-experience and childhood trauma in first-episode schizophrenia. *Comprehensive Psychiatry*. 56:35-41. doi:doi:

132. Healy C, Coughlan H, Clarke M, Kelleher I, Cannon M. What Mediates the Longitudinal Relationship Between Psychotic Experiences and Psychopathology? *Journal of Abnormal Psychology*. 129(5):505-516. doi:doi:

133. Hegelstad WtV, Berg AO, Bjornestad J, et al. Childhood interpersonal trauma and premorbid social adjustment as predictors of symptom remission in first episode psychosis. *Schizophrenia Research*. 232:87-94. doi:doi:

134. Heins M, Simons C, Lataster T, et al. Childhood trauma and psychosis: A case-control and case-sibling comparison across different levels of genetic liability, psychopathology, and type of trauma. *The American Journal of Psychiatry*. 168(12):1286-1294. doi:doi:

135. Heslin M, Desai R, Lappin J, et al. Biological and psychosocial risk factors for psychotic major depression. *Social Psychiatry and Psychiatric Epidemiology: The International Journal for Research in Social and Genetic Epidemiology and Mental Health Services*. 51(2):233-245. doi:doi:

136. Hirt V, Schalinski I, Rockstroh B. Decoding the impact of adverse childhood experiences on the progression of schizophrenia. *Mental Health and Prevention*. 13:82-91. doi:doi:

137. Holshausen K, Bowie CR, Harkness KL. The relation of childhood maltreatment to psychotic symptoms in adolescents and young adults with depression. *Journal of Clinical Child and Adolescent Psychology*. 45(3):241-247. doi:doi:

138. Hou J, Schmitt S, Meller T, et al. Cortical Complexity in People at Ultra-High-Risk for Psychosis Moderated by Childhood Trauma. *Frontiers in psychiatry Frontiers Research Foundation*. 2020 2020;11:594466. doi:doi:

139. Huang YH, Hu HX, Wang LL, et al. Relationships between childhood trauma and dimensional schizotypy: A network analysis and replication. *Asian Journal of Psychiatry*. 2023-7 2023;85:103598. doi:doi:https://dx.doi.org/10.1016/j.ajp.2023.103598

140. Huang Z-H, Hou C-L, Huang Y-H, et al. Individuals at high risk for psychosis experience more childhood trauma, life events and social support deficit in comparison to healthy controls. *Psychiatry Research*. 273:296-302. doi:doi:

141. Hugill M, Fletcher I, Berry K. Investigation of associations between attachment, parenting and schizotypy during the postnatal period. *Journal of Affective Disorders*. 220:86-94. doi:doi:

142. Humphrey C, Berry K, Degnan A, Bucci S. Childhood interpersonal trauma and paranoia in psychosis: The role of disorganised attachment and negative schema. *Schizophrenia Research*. 241:142-148. doi:doi:

143. Haahr UH, Larsen TK, Simonsen E, et al. Relation between premorbid adjustment, duration of untreated psychosis and close interpersonal trauma in first-episode psychosis. *Early Intervention in Psychiatry*. 2018 2018;12(3):316-323. doi:doi:

144. Iacovino JM, Jackson JJ, Oltmanns TF. The relative impact of socioeconomic status and childhood trauma on Black-White differences in paranoid personality disorder symptoms. *Journal of Abnormal Psychology*. 2014-2 2014;123(1):225-30. doi:doi:https://dx.doi.org/10.1037/a0035258

145. Ingec C, Kilicaslan EE. The effect of childhood trauma on age of onset in patients with schizophrenia. *International Journal of Social Psychiatry*. 66(8):763-769. doi:doi:

146. Isvoranu A-M, van Borkulo CD, Boyette L-L, Wigman JT, Vinkers CH, Borsboom D. A network approach to psychosis: Pathways between childhood trauma and psychotic symptoms. *Schizophrenia Bulletin*. 43(1):187-196. doi:doi:

147. Karsinti E, Jarroir M, Zerdazi E-H, et al. Childhood trauma are not associated with the intensity of transient cocaine induced psychotic symptoms. *Psychiatry Research*. 228(3):941-944. doi:doi:

148. Kasznia J, Pytel A, Stanczykiewicz B, et al. Adverse Childhood Experiences and Neurocognition in Schizophrenia Spectrum Disorders: Age at First Exposure and Multiplicity Matter. *Frontiers in psychiatry Frontiers Research Foundation*. 2021 2021;12:684099. doi:doi:

149. Kelly DL, Rowl, LM, et al. Schizophrenia clinical symptom differences in women vs. men with and without a history of childhood physical abuse. *Child and Adolescent Psychiatry and Mental Health Vol 10 2016, ArtID 5*. 10doi:doi:

150. Kennedy SC, Tripodi SJ, Pettus-Davis C. The relationship between childhood abuse and psychosis for women prisoners: Assessing the importance of frequency and type of victimization. *Psychiatric Quarterly*. 84(4):439-453. doi:doi:

151. Kennedy SC, Tripodi SJ, Pettus-Davis C, Ayers J. Examining Dose–Response Relationships Between Childhood Victimization, Depression, Symptoms of Psychosis, and Substance Misuse for Incarcerated Women. *Women and Criminal Justice*. 2016 2016;26(2):77-98. doi:doi:

152. Khosravi M, Bakhshani N-M, Kamangar N. Dissociation as a causal pathway from sexual abuse to positive symptoms in the spectrum of psychotic disorders. *BMC Psychiatry Vol 21 2021, ArtID 266*. 21doi:doi:

153. Kilicaslan EE, Esen AT, Kasal MI, Ozelci E, Boysan M, Gulec M. Childhood trauma, depression, and sleep quality and their association with psychotic symptoms and suicidality in schizophrenia. *Psychiatry Research*. 258:557-564. doi:doi:

154. Kim H, Kim D, Kim SH. Association of types of delusions and hallucinations with childhood abuse and neglect among inpatients with schizophrenia in South Korea: A preliminary study. *Psychosis: Psychological, Social and Integrative Approaches*. 10(3):208-212. doi:doi:

155. Kisely S, Strathearn L, Najman JM. A comparison of psychosis-like symptoms following self-reported and agency-notified child abuse in a population-based birth cohort at 30-year-follow-up. *Schizophrenia Research*. 239:116-122. doi:doi:

156. Kocsis-Bogar K, Meszaros V, Perczel-Forintos D. Gender differences in the relationship of childhood trauma and the course of illness in schizophrenia. *Comprehensive Psychiatry*. 82:84-88. doi:doi:

157. Kramer I, Simons C, Myin-Germeys I, et al. Evidence that genes for depression impact on the pathway from trauma to psychotic-like symptoms by occasioning emotional dysregulation. *Psychological Medicine*. 42(2):283-294. doi:doi:

158. Kramer I, Simons CJ, Wigman JT, et al. Time-lagged moment-to-moment interplay between negative affect and paranoia: New insights in the affective pathway to psychosis. *Schizophrenia Bulletin*. 40(2):278-286. doi:doi:

159. Kraan T, van Dam DS, Velthorst E, et al. Childhood trauma and clinical outcome in patients at ultra-high risk of transition to psychosis. *Schizophrenia Research*. 169(1):193-198. doi:doi:

160. Kraan TC, Ising HK, Fokkema M, et al. The effect of childhood adversity on 4-year outcome in individuals at ultra high risk for psychosis in the Dutch Early Detection Intervention Evaluation (EDIE-NL) Trial. *Psychiatry Research*. 247:55-62. doi:doi:

161. Kraan TC, Velthorst E, Themmen M, et al. Child maltreatment and clinical outcome in individuals at ultra-high risk for psychosis in the EU-GEI high risk study. *Schizophrenia Bulletin*. 44(3):584-592. doi:doi:

162. Lardinois M, Lataster T, Mengelers R, van Os J, Myin-Germeys I. Childhood trauma and increased stress sensitivity in psychosis. *Acta Psychiatrica Scandinavica*. 123(1):28-35. doi:doi:

163. Larsson S, Andreassen OA, Aas M, et al. High prevalence of childhood trauma in patients with schizophrenia spectrum and affective disorder. *Comprehensive Psychiatry*. 54(2):123-127. doi:doi:

164. Laskemoen JF, Aas M, Vaskinn A, et al. Sleep disturbance mediates the link between childhood trauma and clinical outcome in severe mental disorders. *Psychological Medicine*. 51(14):2337-2346. doi:doi:

165. Lau S, Kirchebner J, Kling S, Euler S, Gunther MP. Childhood Maltreatment, Psychopathology, and Offending Behavior in Patients With Schizophrenia: A Latent Class Analysis Evidencing Disparities in Inpatient Treatment Outcome. *Frontiers in psychiatry Frontiers Research Foundation*. 2021 2021;12:612322. doi:doi:

166. Lebovitz JG, Millett CE, Shanahan M, Levy-Carrick NC, Burdick KE. The impact of lifetime interpersonal and intentional trauma on cognition and vulnerability to psychosis in bipolar disorder. *BJPsych Open Vol 7 2021, ArtID e164*. 7doi:doi:

167. Lecei A, ra, Decoster J, et al. Evidence that the association of childhood trauma with psychosis and related psychopathology is not explained by gene-environment correlation: A monozygotic twin differences approach. *Schizophrenia Research*. 205:58-62. doi:doi:

168. Lee EE, Martin AS, Tu X, Palmer BW, Jeste DV. Childhood Adversity and Schizophrenia: The Protective Role of Resilience in Mental and Physical Health and Metabolic Markers. *Journal of Clinical Psychiatry*. 79(3)doi:doi:

169. Lee J-Y, Ban D, Kim S-Y, et al. Negative life events and problematic Internet use as factors associated with psychotic-like experiences in adolescents. *Frontiers in Psychiatry Vol 10 2019, ArtID 369*. 10doi:doi:

170. Lemvigh C, Brouwer R, Hilker R, et al. The relative and interactive impact of multiple risk factors in schizophrenia spectrum disorders: a combined register-based and clinical twin study. *Psychological Medicine*. 2023-11-3 2023;53(4):1266-1276. doi:doi:https://dx.doi.org/10.1017/S0033291721002749

171. Leonhardt BL, Hamm JA, Belanger EA, Lysaker PH. Childhood sexual abuse moderates the relationship of self-reflectivity with increased emotional distress in schizophrenia. *Psychosis: Psychological, Social and Integrative Approaches*. 7(3):195-205. doi:doi:

172. Levit J, Hansen SK, Salisu M, et al. Childhood Trauma and Psychotic Symptomatology in Ethnic Minorities with Schizophrenia. *Schizophrenia Bulletin Open*. 2021-1-1 2021;2doi:doi:https://dx.doi.org/10.1093/schizbullopen/sgaa068

173. Li DJ, Hsieh YC, Chiu CD, Lin CH, Chou LS. The moderation of maternal parenting on the association of trauma, dissociation, and psychosis in depressive inpatients. *European Journal of Psychotraumatology*. 2022 2022;13(1):2024974. doi:doi:

174. Li X-B, Li Q-Y, Liu J-T, Zhang L, Tang Y-L, Wang C-Y. Childhood trauma associates with clinical features of schizophrenia in a sample of Chinese inpatients. *Psychiatry Research*. 228(3):702-707. doi:doi:

175. Li Y, Jinxiang T, Shu Y, et al. Childhood trauma and the plasma levels of IL-6, TNF-alpha are risk factors for major depressive disorder and schizophrenia in adolescents: a cross-sectional and case-control study. *Journal of Affective Disorders*. 10:10. doi:doi:

176. Liu J, Abdin E, Vaingankar JA, et al. Positive mental health framework of transdiagnostic protective factors in elucidating the association between adverse childhood experiences and severe mental disorders. *Australian & New Zealand Journal of Psychiatry*.48674211053568. doi:doi:

177. Liu J, Mahendran R, Chong SA, Subramaniam M. Elucidating the Impact of Childhood, Adulthood, and Cumulative Lifetime Trauma Exposure on Psychiatric Symptoms in Early Schizophrenia Spectrum Disorders. *Journal of Traumatic Stress*. 34(1):137-148. doi:doi:

178. Liu J, Shahwan S, Abdin E, et al. Adverse childhood experiences and positive psychotic symptoms: A nationally representative study in Singapore. *Child Abuse & Neglect*. 2022-9 2022;131:1-8. doi:doi:https://dx.doi.org/10.1016/j.chiabu.2022.105778

179. Liu X, Wolloh Ii MG, Lin X, et al. The association between sibling bullying and psychotic-like experiences among children age 11-16 years in China. *Journal of Affective Disorders*. 2021-11-4 2021;284:31-37. doi:doi:https://dx.doi.org/10.1016/j.jad.2021.01.073

180. Loewy RL, Corey S, Amirfathi F, et al. Childhood trauma and clinical high risk for psychosis. *Schizophrenia Research*. 205:10-14. doi:doi:

181. Longden E, House AO, Waterman MG. Associations between nonauditory hallucinations, dissociation, and childhood adversity in first-episode psychosis. *Journal of Trauma & Dissociation*. 17(5):545-560. doi:doi:

182. Lopes BC. Differences between victims of bullying and nonvictims on levels of paranoid ideation and persecutory symptoms, the presence of aggressive traits, the display of social anxiety and the recall of childhood abuse experiences in a Portuguese mixed clinical sample. *Clinical Psychology & Psychotherapy*. 20(3):254-266. doi:doi:

183. Loureiro CM, Corsi-Zuelli F, Fachim HA, et al. Lifetime cannabis use and childhood trauma increase risk of psychosis in carriers of CNR1 genetic variants: findings from the STREAM study. *Brazilian Journal of Psychiatry*. 2023 2023;45:226-235. doi:doi:https://dx.doi.org/10.47626/1516-4446-2022-2882

184. Lu D, Wang W, Qiu X, et al. The prevalence of confirmed childhood trauma and its' impact on psychotic-like experiences in a sample of Chinese adolescents. *Psychiatry Research*. 287:112897. doi:doi:

185. Luoni C, Agosti M, Crugnola S, Rossi G, Termine C. Psychopathology, dissociation and somatic symptoms in adolescents who were exposed to traumatic experiences. *Frontiers in Psychology Vol 9 2018, ArtID 2390*. 9doi:doi:

186. Luutonen S, Tikka M, Karlsson H, Salokangas R. Childhood trauma and distress experiences associate with psychotic symptoms in patients attending primary and psychiatric outpatient care. Results of the RADEP study. *European Psychiatry*. 28(3):154-160. doi:doi:

187. Mackie CJ, Castellanos-Ryan N, Conrod PJ. Developmental trajectories of psychotic-like experiences across adolescence: impact of victimization and substance use. *Psychological Medicine*. 41(1):47-58. doi:doi:

188. Mackie CJ, O'Leary-Barrett M, Al-Khudhairy N, et al. Adolescent bullying, cannabis use and emerging psychotic experiences: a longitudinal general population study. *Psychological Medicine*. 43(5):1033-1044. doi:doi:

189. Magaud E, Nyman K, Addington J. Cyberbullying in those at clinical high risk for psychosis. *Early Intervention in Psychiatry*. 7(4):427-430. doi:doi:

190. Mall S, Platt JM, Temmingh H, et al. The relationship between childhood trauma and schizophrenia in the genomics of schizophrenia in the Xhosa people (SAX) study in South Africa. *Psychological Medicine*. 50(9):1570-1577. doi:doi:

191. Mansueto G, Faravelli C. Recent life events and psychosis: The role of childhood adversities. *Psychiatry Research*. 256:111-117. doi:doi:

192. Mansueto G, Faravelli C. Stressful life events and psychosis: Gender differences. *Stress & Health*. 38(1):19-30. doi:doi:

193. Mansueto G, Schruers K, Cosci F, et al. Childhood adversities and psychotic symptoms: The potential mediating or moderating role of neurocognition and social cognition. *Schizophrenia Research*. 206:183-193. doi:doi:

194. Mansueto G, Tosato S, Brondino N, et al. Childhood adversity, symptoms, and cortisol in first episode psychosis: a cross-sectional, secondary, observational analysis of a subsample of FEP patients. *Nordic Journal of Psychiatry*. 2023-7 2023;77(5):432-439. doi:doi:https://dx.doi.org/10.1080/08039488.2022.2137846

195. Marlowe NI, Nicholson Perry K, Lee J. Ontological insecurity II: Relationship to attachment, childhood trauma, and subclinical psychotic-like experiences. *Journal of Clinical Psychology*. 76(3):440-460. doi:doi:

196. Martinez AP, Dorahy MJ, Nesbit A, Palmer R, Middleton W. Delusional beliefs and their characteristics: A comparative study between dissociative identity disorder and schizophrenia spectrum disorders. *Journal of Psychiatric Research*. 131:263-268. doi:doi:

197. McCabe KL, Maloney EA, Stain HJ, Loughl, CM, Carr VJ. Relationship between childhood adversity and clinical and cognitive features in schizophrenia. *Journal of Psychiatric Research*. 46(5):600-607. doi:doi:

198. McCarthy-Jones S, Green MJ, Scott RJ, et al. Preliminary evidence of an interaction between the FOXP2 gene and childhood emotional abuse predicting likelihood of auditory verbal hallucinations in schizophrenia. *Journal of Psychiatric Research*. 50:66-72. doi:doi:

199. McDonnell J, Stahl D, Day F, McGuire P, Valmaggia L. Interpersonal sensitivity in those at clinical high risk for psychosis mediates the association between childhood bullying victimisation and paranoid ideation: A virtual reality study. *Schizophrenia Research*. 192:89-95. doi:doi:

200. Mertens YL, Racioppi A, Sheinbaum T, Kwapil T, Barrantes-Vidal N. Dissociation and insecure attachment as mediators of the relation between childhood emotional abuse and nonclinical paranoid traits. *European Journal of Psychotraumatology*. 2021-3-16 2021;12(1):1888539. doi:doi:https://dx.doi.org/10.1080/20008198.2021.1888539

201. Mertin P, O'Brien N. High emotional arousal and failures in reality monitoring: Pathways to auditory hallucinations in non-psychotic children? *Scandinavian Journal of Psychology*. 54(2):102-106. doi:doi:

202. Metel D, Arciszewska A, ra, et al. Mediating role of cognitive biases, resilience and depressive symptoms in the relationship between childhood trauma and psychotic-like experiences in young adults. *Early Intervention in Psychiatry*. 14(1):87-96. doi:doi:

203. Misiak B, Frydecka D. A history of childhood trauma and response to treatment with antipsychotics in first-episode schizophrenia patients: Preliminary results. *Journal of Nervous and Mental Disease*. 204(10):787-792. doi:doi:

204. Misiak B, Moustafa AA, Kiejna A, Frydecka D. Childhood traumatic events and types of auditory verbal hallucinations in first-episode schizophrenia patients. *Comprehensive Psychiatry*. 66:17-22. doi:doi:

205. Misiak B, Szewczuk-Boguslawska M, Samochowiec J, Moustafa AA, Gaweda L. Unraveling the complexity of associations between a history of childhood trauma, psychotic-like experiences, depression and non-suicidal self-injury: A network analysis. *Journal of Affective Disorders*. 2023-9 2023;337:11-17. doi:doi:https://dx.doi.org/10.1016/j.jad.2023.05.044

206. Mongan D, Shannon C, Hanna D, Boyd A, Mulholl, C. The association between specific types of childhood adversity and attenuated psychotic symptoms in a community sample. *Early Intervention in Psychiatry*. 13(2):281-289. doi:doi:

207. Morgan C, Gayer-Anderson C, Beards S, et al. Threat, hostility and violence in childhood and later psychotic disorder: Population-based case-control study. *The British Journal of Psychiatry*. 217(4):575-582. doi:doi:

208. Morkved N, Endsjo M, Winje D, et al. Childhood trauma in schizophrenia spectrum disorder as compared to other mental health disorders. *Psychosis: Psychological, Social and Integrative Approaches*. 9(1):48-56. doi:doi:

209. Morkved N, Winje D, Dovran A, et al. Childhood trauma in schizophrenia spectrum disorders as compared to substance abuse disorders. *Psychiatry Research*. 261:481-487. doi:doi:

210. Muenzenmaier KH, Seixas AA, Schneeberger AR, Castille DM, Battaglia J, Link BG. Cumulative effects of stressful childhood experiences on delusions and hallucinations. *Journal of Trauma & Dissociation*. 16(4):442-462. doi:doi:

211. Munoz-Samons D, Tor J, Rodriguez-Pascual M, et al. Recent stressful life events and stress sensitivity in children and adolescents at clinical risk for psychosis. *Psychiatry Research Vol 303 2021, ArtID 114017*. 303doi:doi:

212. Murphy J, Shevlin M, Houston JE, Adamson G. Modelling the co-occurrence of psychosis-like experiences and childhood sexual abuse. *Social Psychiatry and Psychiatric Epidemiology: The International Journal for Research in Social and Genetic Epidemiology and Mental Health Services*. 49(7):1037-1044. doi:doi:

213. Murphy S, Murphy J, Shevlin M. Negative evaluations of self and others, and peer victimization as mediators of the relationship between childhood adversity and psychotic experiences in adolescence: The moderating role of loneliness. *British Journal of Clinical Psychology*. 54(3):326-344. doi:doi:

214. Mwansisya TE, Yi W, Wang Z, et al. Comparison of psychosocial determinants in inpatients with first-episode and chronic schizophrenia in China. *Archives of Psychiatric Nursing*. 27(1):32-41. doi:doi:

215. Mwesiga EK, Akena D, Koen N, Nakku J, Nakasujja N, Stein DJ. Comparison of antipsychotic naive first-episode psychosis patients and healthy controls in Uganda. *Early intervention in psychiatry*. 15(6):1713-1720. doi:doi:

216. Neumann E, Juckel G, Haussleiter IS. Quality of parental care and traumatic experiences in childhood related to schizophrenic disorders. *Journal of Nervous and Mental Disease*. 208(10):818-821. doi:doi:

217. Newbury J, Arseneault L, Caspi A, et al. Cumulative effects of neighborhood social adversity and personal crime victimization on adolescent psychotic experiences. *Schizophrenia Bulletin*. 44(2):348-358. doi:doi:

218. Niemantsverdriet MBA, Slotema CW, Blom JD, et al. Hallucinations in borderline personality disorder: Prevalence, characteristics and associations with comorbid symptoms and disorders. *Scientific Reports*. 7(1):13920. doi:doi:

219. O'Neill T, Maguire A, Shevlin M. Sexual trauma in childhood and adulthood as predictors of psychotic-like experiences: The mediating role of dissociation. *Child Abuse Review*. 30(5):431-443. doi:doi:

220. Ottesen A, T. V. Hegelstad W, Joa I, et al. Childhood trauma, antipsychotic medication, and symptom remission in first-episode psychosis. *Psychological Medicine*. 2021 2021;doi:doi:

221. Paetzold I, Myin-Germeys I, Schick A, et al. Stress reactivity as a putative mechanism linking childhood trauma with clinical outcomes in individuals at ultra-high-risk for psychosis: Findings from the EU-GEI High Risk Study. *Epidemiology & Psychiatric Science*. 30:e40. doi:doi:

222. Pan PM, Gadelha A, Argolo FC, et al. Childhood trauma and adolescent psychotic experiences in a community-based cohort: The potential role of positive attributes as a protective factor. *Schizophrenia Research*. 205:23-29. doi:doi:

223. Park YM, Shekhtman T, Kelsoe JR. Effect of the Type and Number of Adverse Childhood Experiences and the Timing of Adverse Experiences on Clinical Outcomes in Individuals with Bipolar Disorder. *Brain Sciences*. 10(5):27. doi:doi:

224. Parsaik AK, Abdelgawad N, Chotalia JK, Lane SD, Pigott TA. Early-life trauma in hospitalized patients with mood disorders and its association with clinical outcomes. *Journal of Psychiatric Practice*. 23(1):36-43. doi:doi:

225. Peach N, Alvarez-Jimenez M, Cropper SJ, Sun P, Bendall S. Testing models of post-traumatic intrusions, trauma-related beliefs, hallucinations, and delusions in a first episode psychosis sample. *British Journal of Clinical Psychology*. 58(2):154-172. doi:doi:

226. Pearce J, Simpson J, Berry K, et al. Attachment and dissociation as mediators of the link between childhood trauma and psychotic experiences. *Clinical Psychology & Psychotherapy*. 24(6):1304-1312. doi:doi:

227. Perry A, Gordon-Smith K, Di Florio A, et al. Adverse childhood life events and postpartum psychosis in bipolar disorder. *Journal of Affective Disorders*. 205:69-72. doi:doi:

228. Petrovic M, Injac Stevovic L. Intergeneration Transmission of Violence in Forensic Patients With a Diagnosis of Schizophrenia and Psychosis: Was Parental Alcoholic Abuse a Significant Factor? *Frontiers in psychiatry Frontiers Research Foundation*. 2021 2021;12:765279. doi:doi:

229. Pfeifer S, Krabbendam L, Myin-Germeys I, et al. A cognitive intermediate phenotype study confirming possible gene-early adversity interaction in psychosis outcome: A general population twin study. *Psychosis-Psychological Social and Integrative Approaches*. 2010 2010;2(1):1-11. doi:doi:

230. Phassouliotis C, Garner BA, Phillips LJ, et al. Enhanced cortisol suppression following administration of low-dose dexamethasone in first-episode psychosis patients. *Australian and New Zealand Journal of Psychiatry*. 47(4):363-370. doi:doi:

231. Pietrek C, Elbert T, Weierstall R, Muller O, Rockstroh B. Childhood adversities in relation to psychiatric disorders. *Psychiatry Research*. 206(1):103-110. doi:doi:

232. Pignon B, Lajnef M, Kirkbride JB, et al. The Independent Effects of Psychosocial Stressors on Subclinical Psychosis: Findings From the Multinational EU-GEI Study. *Schizophrenia Bulletin*. 47(6):1674-1684. doi:doi:

233. Pignon B, Peyre H, Ayrolles A, et al. Genetic and psychosocial stressors have independent effects on the level of subclinical psychosis: findings from the multinational EU-GEI study. *Epidemiology & Psychiatric Science*. 2022-9-27 2022;31:e68. doi:doi:https://dx.doi.org/10.1017/S2045796022000464

234. Pilton M, Bucci S, ra, et al. Does insecure attachment mediate the relationship between trauma and voice-hearing in psychosis? *Psychiatry Research*. 246:776-782. doi:doi:

235. Pinckaers FM, Rotee IL, Nwosu C, et al. Evidence for interaction between genetic liability and childhood trauma in the development of psychotic symptoms. *Social Psychiatry and Psychiatric Epidemiology: The International Journal for Research in Social and Genetic Epidemiology and Mental Health Services*. 54(9):1045-1054. doi:doi:

236. Pionke R, Gidzgier P, Nelson B, Gaweda L. Prevalence, dimensionality and clinical relevance of self-disturbances and psychotic-like experiences in polish young adults: A latent class analysis approach. *International Journal of Methods in Psychiatric Research Vol 29(1), 2020, ArtID e1809*. 29(1)doi:doi:

237. Pionke-Ubych R, Frydecka D, Cechnicki A, Krezolek M, Nelson B, Gaweda L. Integrating trauma, self-disturbances, cognitive biases, and personality into a model for the risk of psychosis: a longitudinal study in a non-clinical sample. *European Archives of Psychiatry and Clinical Neuroscience*.13. doi:doi:

238. Post D, Veling W. Sexual minority status, social adversity and risk for psychotic disorders-results from the GROUP study. *Psychological Medicine*. 51(5):770-776. doi:doi:

239. Powers A, Fani N, Cross D, Ressler KJ, Bradley B. Childhood trauma, PTSD, and psychosis: Findings from a highly traumatized, minority sample. *Child Abuse & Neglect*. 58:111-118. doi:doi:

240. Powers AD, Thomas KM, Ressler KJ, Bradleya B. The differential effects of child abuse and posttraumatic stress disorder on schizotypal personality disorder. *Comprehensive Psychiatry*. 52(4):438-445. doi:doi:

241. Pries L, Klingenberg B, Menne-Lothmann C, et al. Polygenic liability for schizophrenia and childhood adversity influences daily-life emotion dysregulation and psychosis proneness. *Acta Psychiatrica Scandinavica*. 141(5):465-475. doi:doi:

242. Prokopez CR, Vallejos M, Farinola R, et al. The history of multiple adverse childhood experiences in patients with schizophrenia is associated with more severe symptomatology and suicidal behavior with gender-specific characteristics. *Psychiatry Research*. 293:113411. doi:doi:

243. Pruessner M, King S, Veru F, et al. Impact of childhood trauma on positive and negative symptom remission in first episode psychosis. *Schizophrenia Research*. 231:82-89. doi:doi:

244. Pruessner M, King S, Vracotas N, et al. Gender differences in childhood trauma in first episode psychosis: Association with symptom severity over two years. *Schizophrenia Research*. 205:30-37. doi:doi:

245. Qiao Z, Lafit G, Lecei A, et al. Childhood Adversity and Emerging Psychotic Experiences: A Network Perspective. *Schizophrenia Bulletin*. 2023-6-15 2023;15:15. doi:doi:https://dx.doi.org/10.1093/schbul/sbad079

246. Quide Y, Cohen-Woods S, O'Reilly N, Carr VJ, Elzinga BM, Green MJ. Schizotypal personality traits and social cognition are associated with childhood trauma exposure. *British Journal of Clinical Psychology*. 57(4):397-419. doi:doi:

247. Rajkumar RP. The Impact of Childhood Adversity on the Clinical Features of Schizophrenia. *Schizophrenia Research & Treatment Print*. 2015 2015;2015:532082. doi:doi:

248. Ramsay CE, Flanagan P, Gantt S, Broussard B, Compton MT. Clinical correlates of maltreatment and traumatic experiences in childhood and adolescence among predominantly African American, socially disadvantaged, hospitalized, first-episode psychosis patients. *Psychiatry Research*. 188(3):343-349. doi:doi:

249. Rapado-Castro M, Whittle S, Pantelis C, et al. Does cortical brain morphology act as a mediator between childhood trauma and transition to psychosis in young individuals at ultra-high risk? *Schizophrenia Research*. 224:116-125. doi:doi:

250. Ratanatharathorn A, Koenen KC, Chibnik LB, Weisskopf MG, Rich-Edwards JW, Roberts AL. Polygenic risk for autism, attention-deficit hyperactivity disorder, schizophrenia, major depressive disorder, and neuroticism is associated with the experience of childhood abuse. *Molecular Psychiatry*. 26(5):1696-1705. doi:doi:

251. Rauschenberg C, van Os J, Cremers D, Goedhart M, Schieveld J, Reininghaus U. Stress sensitivity as a putative mechanism linking childhood trauma and psychopathology in youth's daily life. *Acta Psychiatrica Scandinavica*. 136(4):373-388. doi:doi:

252. Rauschenberg C, van Os J, Goedhart M, Schieveld JNM, Reininghaus U. Bullying victimization and stress sensitivity in help-seeking youth: findings from an experience sampling study. *European Child & Adolescent Psychiatry*. 2021 2021;30(4):591-605. doi:doi:

253. Reininghaus U, Gayer-Anderson C, Valmaggia L, et al. Psychological processes underlying the association between childhood trauma and psychosis in daily life: An experience sampling study. *Psychological Medicine*. 46(13):2799-2813. doi:doi:

254. Riley G, Perrin M, Vaez-Azizi LM, et al. Telomere length and early trauma in schizophrenia. *Schizophrenia Research*. 199:426-430. doi:doi:

255. Rosen C, McCarthy-Jones S, Chase KA, et al. The role of inner speech on the association between childhood adversity and 'hearing voices'. *Psychiatry Research*. 286:112866. doi:doi:

256. Rossler W, Ajdacic-Gross V, Rodgers S, Haker H, Muller M. Childhood trauma as a risk factor for the onset of subclinical psychotic experiences: Exploring the mediating effect of stress sensitivity in a cross-sectional epidemiological community study. *Schizophrenia Research*. 172(1):46-53. doi:doi:

257. Ruby E, Rothman K, Corcoran C, Goetz RR, Malaspina D. Influence of early trauma on features of schizophrenia. *Early Intervention in Psychiatry*. 11(4):322-333. doi:doi:

258. Russo DA, Stochl J, Painter M, et al. Trauma history characteristics associated with mental states at clinical high risk for psychosis. *Psychiatry Research*. 220(1):237-244. doi:doi:

259. Sahin S, Yuksel C, Guler J, et al. The history of childhood trauma among individuals with ultra high risk for psychosis is as common as among patients with first-episode schizophrenia. *Early Intervention in Psychiatry*. 7(4):414-420. doi:doi:

260. Salokangas RK, Schultze-Lutter F, Hietala J, et al. Depression predicts persistence of paranoia in clinical high-risk patients to psychosis: Results of the EPOS project. *Social Psychiatry and Psychiatric Epidemiology: The International Journal for Research in Social and Genetic Epidemiology and Mental Health Services*. 51(2):247-257. doi:doi:

261. Sayin A, Yuksel N, Konac E, et al. Effects of the adverse life events and Disrupted in Schizophrenia-1 (DISC1) gene polymorphisms on acute symptoms of schizophrenia. *DNA & Cell Biology*. 32(2):73-80. doi:doi:

262. Schalinski I, Breinlinger S, Hirt V, Teicher MH, Odenwald M, Rockstroh B. Environmental adversities and psychotic symptoms: The impact of timing of trauma, abuse, and neglect. *Schizophrenia Research*. 205:4-9. doi:doi:

263. Schalinski I, Fischer Y, Rockstroh B. Impact of childhood adversities on the short-term course of illness in psychotic spectrum disorders. *Psychiatry Research*. 228(3):633-640. doi:doi:

264. Schalinski I, Teicher MH. Type and timing of childhood maltreatment and severity of shutdown dissociation in patients with schizophrenia spectrum disorder. *PLoS ONE [Electronic Resource]*. 2015 2015;10(5):e0127151. doi:doi:

265. Scott M, Rossell SL, Meyer D, Toh WL, Thomas N. Childhood trauma, attachment and negative schemas in relation to negative auditory verbal hallucination (AVH) content. *Psychiatry Research Vol 290 2020, ArtID 112997*. 290doi:doi:

266. Seidenfaden D, Knorr U, Soendergaard MG, et al. The relationship between self-reported childhood adversities, adulthood psychopathology and psychological stress markers in patients with schizophrenia. *Comprehensive Psychiatry*. 72:48-55. doi:doi:

267. Sengutta M, Gaweda L, Moritz S, Karow A. The mediating role of borderline personality features in the relationship between childhood trauma and psychotic-like experiences in a sample of help-seeking non-psychotic adolescents and young adults. *European Psychiatry*. 56:84-90. doi:doi:

268. Sengutta M, Karow A, Gaweda L. Anomalous self-experiences (ASE) in relation to clinical high risk for psychosis (CHRP), childhood trauma and general psychopathology among adolescent and young adult help seekers. *Schizophrenia Research*. 237:182-189. doi:doi:

269. Seo J, Choi JY. Social defeat as a mediator of the relationship between childhood trauma and paranoid ideation. *Psychiatry Research*. 260:48-52. doi:doi:

270. Setien-Suero E, Ayesa-Arriola R, Pena J, Crespo-Facorro B, Ojeda N. Trauma and psychosis: The mediating role of premorbid adjustment and recent stressful events in a 3-year longitudinal study. *Journal of Psychiatric Research*. 2022-11 2022;155:279-285. doi:doi:https://dx.doi.org/10.1016/j.jpsychires.2022.09.029

271. Shakoor S, McGuire P, Cardno AG, Freeman D, Plomin R, Ronald A. A shared genetic propensity underlies experiences of bullying victimization in late childhood and self-rated paranoid thinking in adolescence. *Schizophrenia Bulletin*. 41(3):754-763. doi:doi:

272. Sheffield JM, Williams LE, Blackford JU, Heckers S. Childhood sexual abuse increases risk of auditory hallucinations in psychotic disorders. *Comprehensive Psychiatry*. 54(7):1098-1104. doi:doi:

273. Sheinbaum T, Kwapil TR, Barrantes-Vidal N. Fearful attachment mediates the association of childhood trauma with schizotypy and psychotic-like experiences. *Psychiatry Research*. 220(1):691-693. doi:doi:

274. Sheinbaum T, Racioppi A, Kwapil TR, Barrantes-Vidal N. Attachment as a mechanism between childhood maltreatment and subclinical psychotic phenomena: Results from an eight-year follow-up study. *Schizophrenia Research*. 220:261-264. doi:doi:

275. Sideli L, Fisher HL, Murray RM, et al. Interaction between cannabis consumption and childhood abuse in psychotic disorders: Preliminary findings on the role of different patterns of cannabis use. *Early Intervention in Psychiatry*. 12(2):135-142. doi:doi:

276. Simpson J, Helliwell B, Varese F, Powell P. Self-disgust mediates the relationship between childhood adversities and psychosis. *British Journal of Clinical Psychology*. 59(2):260-275. doi:doi:

277. Simpson S, Phillips L, Baksheev G, et al. Stability of retrospective self-reports of childhood trauma in first-episode psychosis. *Early Intervention in Psychiatry*. 13(4):908-913. doi:doi:

278. Solesvik M, Joa I, Larsen TK, et al. Visual hallucinations in first-episode psychosis: Association with childhood trauma. *PLoS ONE Vol 11(5), 2016, ArtID e0153458*. 11(5)doi:doi:

279. Sommer IE, Daalman K, Rietkerk T, et al. Healthy individuals with auditory verbal hallucinations; Who are they? Psychiatric assessments of a selected sample of 103 subjects. *Schizophrenia Bulletin*. 36(3):633-641. doi:doi:

280. Stain HJ, Bronnick K, Hegelstad WT, et al. Impact of interpersonal trauma on the social functioning of adults with first-episode psychosis. *Schizophrenia Bulletin*. 40(6):1491-1498. doi:doi:

281. Stalheim J, Berglund K, Berggren U, Balldin J, Fahlke C. Psychotic experiences, childhood trauma, and alcohol-related self-efficacy in a nonpsychiatric sample of individuals in alcohol dependence treatment: A pilot study. *Alcoholism Treatment Quarterly*. 2018 2018;36(3):387-398. doi:doi:

282. Steenkamp L, Weijers J, Gerrmann J, Eurelings-Bontekoe E, Selten JP. The relationship between childhood abuse and severity of psychosis is mediated by loneliness: an experience sampling study. *Schizophrenia Research*. 30:30. doi:doi:

283. Steenkamp LR, Tiemeier H, Bolhuis K, Hillegers MHJ, Kushner SA, Blanken LME. Peer-reported bullying, rejection and hallucinatory experiences in childhood. *Acta Psychiatrica Scandinavica*. 143(6):503-512. doi:doi:

284. Stevens LH, Turkington D, Drage L, et al. Investigation of a traumatic psychosis subgroup: A cluster analysis of an antipsychotic free cohort. *Psychosis: Psychological, Social and Integrative Approaches*. 11(4):298-307. doi:doi:

285. Stilo SA, Gayer-Anderson C, Beards S, et al. Further evidence of a cumulative effect of social disadvantage on risk of psychosis. *Psychological Medicine*. 2017-4 2017;47(5):913-924. doi:doi:https://dx.doi.org/10.1017/S0033291716002993

286. Stone LMD, Millman ZB, Ongur D, Shinn AK. The Intersection Between Childhood Trauma, the COVID-19 Pandemic, and Trauma-related and Psychotic Symptoms in People With Psychotic Disorders. *Schizophrenia Bulletin Open*. 2(1):sgab050. doi:doi:

287. Storvestre GB, Jensen A, Bjerke E, et al. Childhood Trauma in Persons With Schizophrenia and a History of Interpersonal Violence. *Frontiers in psychiatry Frontiers Research Foundation*. 2020 2020;11:383. doi:doi:

288. Stramecki F, Frydecka D, Gaweda L, et al. The Impact of the FKBP5 Gene Polymorphisms on the Relationship between Traumatic Life Events and Psychotic-Like Experiences in Non-Clinical Adults. *Brain Sciences*. 11(5):28. doi:doi:

289. Struck N, Krug A, Yuksel D, et al. Childhood maltreatment and adult mental disorders - the prevalence of different types of maltreatment and associations with age of onset and severity of symptoms. *Psychiatry Research*. 293:113398. doi:doi:

290. Sun M, Hu X, Zhang W, et al. Psychotic-like experiences and associated socio-demographic factors among adolescents in China. *Schizophrenia Research*. 166(1):49-54. doi:doi:

291. Sun M, Wang D, Jing L, Zhou L. Changes in psychotic-like experiences and related influential factors in technical secondary school and college students during COVID-19. *Schizophrenia Research*. 231:3-9. doi:doi:

292. Sun M, Xue Z, Zhang W, et al. Psychotic-like experiences, trauma and related risk factors among "left-behind" children in China. *Schizophrenia Research*. 181:43-48. doi:doi:

293. Sun M, Zhang W, Guo R, et al. Psychotic-like experiences and correlation with childhood trauma and other socio-demographic factors: A cross-sectional survey in adolescence and early adulthood in China. *Psychiatry Research*. 255:272-277. doi:doi:

294. Sun P, Alvarez-Jimenez M, Simpson K, Lawrence K, Peach N, Bendall S. Does dissociation mediate the relationship between childhood trauma and hallucinations, delusions in first episode psychosis? *Comprehensive Psychiatry*. 84:68-74. doi:doi:

295. Tang W, Xu D, Yang Y, Xu J. Psychotic-like experiences in Chinese children and adolescents: The effect of earthquake exposure, maltreatment and negative life events. *Early intervention in psychiatry*. 15(3):536-546. doi:doi:

296. Thomas EHX, Rossell SL, Gurvich C. Gender Differences in the Correlations between Childhood Trauma, Schizotypy and Negative Emotions in Non-Clinical Individuals. *Brain Sciences*. 12(2):29. doi:doi:

297. Thompson A, Marwaha S, Nelson B, et al. Do affective or dissociative symptoms mediate the association between childhood sexual trauma and transition to psychosis in an ultra-high risk cohort? *Psychiatry Research*. 236:182-185. doi:doi:

298. Thompson AD, Nelson B, Yuen HP, et al. Sexual trauma increases the risk of developing psychosis in an ultra high-risk "prodromal" population. *Schizophrenia Bulletin*. 40(3):697-706. doi:doi:

299. Tikka M, Luutonen S, Ilonen T, et al. Childhood trauma and premorbid adjustment among individuals at clinical high risk for psychosis and normal control subjects. *Early Intervention in Psychiatry*. 7(1):51-57. doi:doi:

300. Tomassi S, Tosato S, Mondelli V, et al. Influence of childhood trauma on diagnosis and substance use in first-episode psychosis. *The British Journal of Psychiatry*. 211(3):151-156. doi:doi:

301. Tonini E, Quide Y, Whitford TJ, Green MJ. Cumulative sociodemographic disadvantage partially mediates associations between childhood trauma and schizotypy. *British Journal of Clinical Psychology*. 24:24. doi:doi:

302. Toutountzidis D, Gale TM, Irvine K, Sharma S, Laws KR. Sex differences in the association between childhood adversities and schizotypal personality traits. *Psychiatry Research*. 269:31-37. doi:doi:

303. Trauelsen AM, Bendall S, Jansen JE, et al. Childhood adversity specificity and dose-response effect in non-affective first-episode psychosis. *Schizophrenia Research*. 165(1):52-59. doi:doi:

304. Trotta A, Di Forti M, Iyegbe C, et al. Familial risk and childhood adversity interplay in the onset of psychosis. *BJPsych Open*. 1(1):6-13. doi:doi:

305. Trotta A, Iyegbe C, Di Forti M, et al. Interplay between schizophrenia polygenic risk score and childhood adversity in first-presentation psychotic disorder: A pilot study. *PLoS ONE Vol 11(9), 2016, ArtID e0163319*. 11(9)doi:doi:

306. Trotta A, Iyegbe C, Yiend J, et al. Interaction between childhood adversity and functional polymorphisms in the dopamine pathway on first-episode psychosis. *Schizophrenia Research*. 205:51-57. doi:doi:

307. Trotta A, Murray RM, David AS, et al. Impact of different childhood adversities on 1-year outcomes of psychotic disorder in the genetics and psychosis study. *Schizophrenia Bulletin*. 42(2):464-475. doi:doi:

308. Trotta G, Rodriguez V, Quattrone D, et al. Cannabis use as a potential mediator between childhood adversity and first-episode psychosis: results from the EU-GEI case-control study. *Psychological Medicine*. 2023-5 2023:10. doi:doi:10.1017/s0033291723000995

309. Trovao LD, Alves GS, Carrilho CG, et al. Early trauma and schizophrenia onset: preliminary results of an outpatient cohort in Brazil. *Trends in Psychiatry and Psychotherapy*. 2022 2022;44:10. doi:doi:10.47626/2237-6089-2020-0024

310. Uyan TT, Baltacioglu M, Hocaoglu C. Relationships between childhood trauma and dissociative, psychotic symptoms in patients with schizophrenia: a case-control study. *General Psychiatry*. 2022 2022;35(1):e100659. doi:doi:

311. Valmaggia L, Day F, Kroll J, et al. Bullying victimisation and paranoid ideation in people at ultra high risk for psychosis. *Schizophrenia Research*. 168(1):68-73. doi:doi:

312. van Bergen AH, Verkooijen S, Vreeker A, et al. The characteristics of psychotic features in bipolar disorder. *Psychological Medicine*. 49(12):2036-2048. doi:doi:

313. van Dam D, Korver-Nieberg N, Velthorst E, Meijer C, de Haan L. Childhood maltreatment, adult attachment and psychotic symptomatology: A study in patients, siblings and controls. *Social Psychiatry and Psychiatric Epidemiology: The International Journal for Research in Social and Genetic Epidemiology and Mental Health Services*. 49(11):1759-1767. doi:doi:

314. van Dam D, van Nierop M, Viechtbauer W, et al. Childhood abuse and neglect in relation to the presence and persistence of psychotic and depressive symptomatology. *Psychological Medicine*. 45(7):1363-1377. doi:doi:

315. van Nierop M, Bak M, de Graaf R, ten Have M, van Dorsselaer S, van Winkel R. The functional and clinical relevance of childhood trauma-related admixture of affective, anxious and psychosis symptoms. *Acta Psychiatrica Scandinavica*. 133(2):91-101. doi:doi:

316. van Nierop M, Janssens M, Genetic Risk OoPI, et al. Evidence that transition from health to psychotic disorder can be traced to semi-ubiquitous environmental effects operating against background genetic risk. *PLoS ONE [Electronic Resource]*. 2013 2013;8(11):e76690. doi:doi:

317. van Nierop M, Lecei A, ra, et al. Stress reactivity links childhood trauma exposure to an admixture of depressive, anxiety, and psychosis symptoms. *Psychiatry Research*. 260:451-457. doi:doi:

318. van Os J, Pries LK, Ten Have M, et al. Evidence, and replication thereof, that molecular-genetic and environmental risks for psychosis impact through an affective pathway. *Psychological Medicine*.1-13. doi:doi:

319. Varese F, Barkus E, Bentall R. Dissociation mediates the relationship between childhood trauma and hallucination-proneness. *Psychological Medicine*. 42(5):1025-1036. doi:doi:

320. Veling W, Counotte J, Pot-Kolder R, van Os J, van der Gaag M. Childhood trauma, psychosis liability and social stress reactivity: A virtual reality study. *Psychological Medicine*. 46(16):3339-3348. doi:doi:

321. Vila-Badia R, Del Cacho N, Butjosa A, et al. Prevalence and types of childhood trauma in first episode psychosis patients. Relation with clinical onset variables. *Journal of Psychiatric Research*. 146:102-108. doi:doi:

322. Vila-Badia R, Serra-Arumí C, Butjosa A, et al. Risk and protective factors for the appearance of first-episode psychosis: The role of childhood trauma and coping strategies. *Asian Journal of Psychiatry*. 2022 2022;78doi:doi:10.1016/j.ajp.2022.103284

323. Vinkers CH, van Gastel WA, Schubart CD, et al. The effect of childhood maltreatment and cannabis use on adult psychotic symptoms is modified by the COMT Val158 Met polymorphism. *Schizophrenia Research*. 150(1):303-311. doi:doi:

324. Vivalya BM, Bin Kitoko GM, Nzanzu AK, et al. Affective and Psychotic Disorders in War-Torn Eastern Part of the Democratic Republic of the Congo: A Cross-Sectional Study. *Psychiatry Journal Print*. 2020 2020;2020:9190214. doi:doi:

325. Vogel M, Meier J, Gronke S, et al. Differential effects of childhood abuse and neglect: Mediation by posttraumatic distress in neurotic disorder and negative symptoms in schizophrenia? *Psychiatry Research*. 189(1):121-127. doi:doi:

326. Wang L, Yin Y, Zhou Y, et al. The mediating effect of brain-derived neurotrophic factor levels on childhood trauma and psychiatric symptoms in patients with first-episode schizophrenia. *Australian & New Zealand Journal of Psychiatry*.48674211031478. doi:doi:

327. Wang Z, Xue Z, Pu W, et al. Comparison of first-episode and chronic patients diagnosed with schizophrenia: Symptoms and childhood trauma. *Early Intervention in Psychiatry*. 7(1):23-30. doi:doi:

328. Wei Q, Pan Y, Zhang S, et al. Epidemiology of childhood trauma and its association with insomnia and psychotic-like experiences in Chinese Zhuang adolescents. *Frontiers in psychiatry Frontiers Research Foundation*. 2022 2022;13:974674. doi:doi:https://dx.doi.org/10.3389/fpsyt.2022.974674

329. Weijers J, Fonagy P, Eurelings-Bontekoe E, Termorshuizen F, Viechtbauer W, Selten J. Mentalizing impairment as a mediator between reported childhood abuse and outcome in nonaffective psychotic disorder. *Psychiatry Research*. 259:463-469. doi:doi:

330. Weitkamper A, Kellner M, Iffl, et al. Childhood Maltreatment in Individuals With Schizophrenia Spectrum Disorders: The Impact of Cut-Off Scores on Prevalence Rates. *Frontiers in psychiatry Frontiers Research Foundation*. 2021 2021;12:692492. doi:doi:

331. Wickham S, Bentall R. Are specific early-life adversities associated with specific symptoms of psychosis?: A patient study considering just world beliefs as a mediator. *Journal of Nervous and Mental Disease*. 204(8):606-613. doi:doi:

332. Wigman JT, van Winkel R, Jacobs N, et al. A twin study of genetic and environmental determinants of abnormal persistence of psychotic experiences in young adulthood. *American Journal of Medical Genetics Part B: Neuropsychiatric Genetics*. 156(5):546-552. doi:doi:

333. Yamasaki S, Ando S, Koike S, et al. Dissociation mediates the relationship between peer victimization and hallucinatory experiences among early adolescents. *Schizophrenia Research*. 4:18-23. doi:doi:

334. Yousef AM, Mohamed AE, Eldeeb SM, Mahdy RS. Prevalence and clinical implication of adverse childhood experiences and their association with substance use disorder among patients with schizophrenia. *Egyptian Journal of Neurology, Psychiatry and Neurosurgery*. 58doi:doi:

335. Zhang J, Liu Z, Long Y, et al. Mediating role of impaired wisdom in the relation between childhood trauma and psychotic-like experiences in Chinese college students: A nationwide cross-sectional study. *BMC Psychiatry Vol 22, 2022, ArtID 655*. 2022-10 2022;22doi:doi:https://dx.doi.org/10.1186/s12888-022-04270-x

336. Zhang L, Zhao N, Zhu M, Tang M, Liu W, Hong W. Adverse childhood experiences in patients with schizophrenia: related factors and clinical implications. *Frontiers in psychiatry Frontiers Research Foundation*. 2023 2023;14:1247063. doi:doi:https://dx.doi.org/10.3389/fpsyt.2023.1247063

337. Zhang Y, Liu J, Chen L, et al. Prevalence of co-occurring severe depression and psychotic symptoms in college students and its relationship with childhood maltreatment. *Child Abuse & Neglect*. 2023-9-23 2023;146:106470. doi:doi:https://dx.doi.org/10.1016/j.chiabu.2023.106470

338. Zhao J, Lu XH, Liu Y, et al. The Unique Contribution of Past Bullying Experiences to the Presence of Psychosis-Like Experiences in University Students. *Frontiers in psychiatry Frontiers Research Foundation*. 2022 2022;13:839630. doi:doi:https://dx.doi.org/10.3389/fpsyt.2022.839630

339. Zimbron J, de Azua SR, Kh, et al. Clinical and sociodemographic comparison of people at high-risk for psychosis and with first-episode psychosis. *Acta Psychiatrica Scandinavica*. 127(3):210-216. doi:doi:

340. Østefjells T, Lystad JU, Berg AO, et al. Metacognitive beliefs mediate the effect of emotional abuse on depressive and psychotic symptoms in severe mental disorders. *Psychological Medicine*. 2017 2017;47(13):2323-2333. doi:doi:

341. Aas M, Andreassen OA, Aminoff SR, et al. A history of childhood trauma is associated with slower improvement rates: Findings from a one-year follow-up study of patients with a first-episode psychosis. *BMC Psychiatry Vol 16 2016, ArtID 126*. 16doi:doi:

# Table 4. Reference list of excluded studies with reasons after full-text analysis

1. Bi-directional relationship found between child trauma and psychosis. *Brown University Child & Adolescent Psychopharmacology Update*. 2013;15(9):5-6. RAYYAN-INCLUSION: {"Sjur"=>"Included", "Wenche ten Velden"=>"Included"} | RAYYAN-INCLUSION: {"Sjur"=>"Excluded", "Wenche ten Velden"=>"Excluded"} | RAYYAN-EXCLUSION-REASONS: wrong publication type.

2. Adams RE, Ritter C, Bonfine N. Epidemiology of trauma: Childhood adversities, neighborhood problems, discrimination, chronic strains, life events, and daily hassles among people with a severe mental illness. *Psychiatry Research*. 230(2):609-615. RAYYAN-INCLUSION: {"Sjur"=>"Included", "Wenche ten Velden"=>"Included"} | RAYYAN-INCLUSION: {"Sjur"=>"Excluded", "Wenche ten Velden"=>"Excluded"} | RAYYAN-EXCLUSION-REASONS: wrong outcome.

3. Adanty C, Qian J, Wang Y, et al. Childhood trauma exposure and personality traits in schizophrenia patients. *Schizophrenia Research*. 241:221-227. RAYYAN-INCLUSION: {"Sjur"=>"Included", "Wenche ten Velden"=>"Included"} | RAYYAN-INCLUSION: {"Sjur"=>"Excluded", "Wenche ten Velden"=>"Excluded"} | RAYYAN-EXCLUSION-REASONS: wrong outcome.

4. Ajnakina O, Trotta A, Forti MD, et al. Different types of childhood adversity and 5-year outcomes in a longitudinal cohort of first-episode psychosis patients. *Psychiatry Research*. 2018-11-11 2018;269:199-206. doi:doi:https://dx.doi.org/10.1016/j.psychres.2018.08.054. RAYYAN-INCLUSION: {"Sjur"=>"Included"} | RAYYAN-INCLUSION: {"Sjur"=>"Excluded"} | RAYYAN-EXCLUSION-REASONS: wrong outcome

5. Alameda L, Ferrari C, Baumann P, Gholam-Rezaee M, Do K, Conus P. Childhood sexual and physical abuse: Age at exposure modulates impact on functional outcome in early psychosis patients. *Psychological Medicine*. 45(13):2727-2736. RAYYAN-INCLUSION: {"Sjur"=>"Included", "Wenche ten Velden"=>"Included"} | RAYYAN-INCLUSION: {"Sjur"=>"Excluded", "Wenche ten Velden"=>"Excluded"} | RAYYAN-EXCLUSION-REASONS: wrong outcome.

6. Alameda L, Golay P, Baumann PS, et al. Mild depressive symptoms mediate the impact of childhood trauma on long-term functional outcome in early psychosis patients. *Schizophrenia Bulletin*. 43(5):1027-1035. RAYYAN-INCLUSION: {"Sjur"=>"Included", "Wenche ten Velden"=>"Included"} | RAYYAN-INCLUSION: {"Sjur"=>"Excluded", "Wenche ten Velden"=>"Excluded"} | RAYYAN-EXCLUSION-REASONS: wrong outcome.

7. Amone-P'Olak K, Elklit A. Interpersonal sensitivity as mediator of the relations between war experiences and mental illness in war-affected youth in northern Uganda: Findings from the WAYS study. *Traumatology*. 24(3):200-208. RAYYAN-INCLUSION: {"Sjur"=>"Included", "Wenche ten Velden"=>"Included"} | RAYYAN-INCLUSION: {"Sjur"=>"Excluded", "Wenche ten Velden"=>"Excluded"} | RAYYAN-LABELS: uknown age of exposure | RAYYAN-EXCLUSION-REASONS: no age.

8. Amone-P'Olak K, Otim BN, Opio G, Ovuga E, Meiser-Stedman R. War experiences and psychotic symptoms among former child soldiers in Northern Uganda: The mediating role of post-war hardships-The WAYS Study. *South African Journal of Psychology*. 45(2):155-167. RAYYAN-INCLUSION: {"Sjur"=>"Included", "Wenche ten Velden"=>"Included"} | RAYYAN-INCLUSION: {"Sjur"=>"Excluded", "Wenche ten Velden"=>"Excluded"} | RAYYAN-LABELS: wrong independent variable | RAYYAN-EXCLUSION-REASONS: wrong predictor.

9. Amsel LV, Hunter N, Kim S, Fodor KE, Markowitz JC. Does a study focused on trauma encourage patients with psychotic symptoms to seek treatment? *Psychiatric Services*. 63(4):386-389. RAYYAN-INCLUSION: {"Sjur"=>"Included", "Wenche ten Velden"=>"Included"} | RAYYAN-INCLUSION: {"Sjur"=>"Excluded", "Wenche ten Velden"=>"Excluded"} | RAYYAN-EXCLUSION-REASONS: wrong study design.

10. Andorko ND, Millman ZB, Klingaman E, et al. Association between sleep, childhood trauma and psychosis-like experiences. *Schizophrenia Research*. 199:333-340. RAYYAN-INCLUSION: {"Sjur"=>"Included", "Wenche ten Velden"=>"Included"} | RAYYAN-INCLUSION: {"Sjur"=>"Excluded", "Wenche ten Velden"=>"Excluded"} | RAYYAN-LABELS: uknown age of exposure | RAYYAN-EXCLUSION-REASONS: no age.

11. Anglin DM, Espinosa A, Barada B, et al. Comparing the Role of Aberrant Salience and Dissociation in the Relation between Cumulative Traumatic Life Events and Psychotic-Like Experiences in a Multi-Ethnic Sample. *Journal of Clinical Medicine*. 8(8):14. RAYYAN-INCLUSION: {"Sjur"=>"Included", "Wenche ten Velden"=>"Included"} | RAYYAN-INCLUSION: {"Sjur"=>"Excluded", "Wenche ten Velden"=>"Excluded"} | RAYYAN-LABELS: uknown age of exposure | RAYYAN-EXCLUSION-REASONS: no age.

12. Anglin DM, Polanco-Roman L, Lui F. Ethnic Variation in Whether Dissociation Mediates the Relation Between Traumatic Life Events and Attenuated Positive Psychotic Symptoms. *Journal of Trauma & Dissociation*. 16(1):68-85. RAYYAN-INCLUSION: {"Sjur"=>"Included", "Wenche ten Velden"=>"Included"} | RAYYAN-INCLUSION: {"Sjur"=>"Excluded", "Wenche ten Velden"=>"Excluded"} | RAYYAN-LABELS: uknown age of exposure | RAYYAN-EXCLUSION-REASONS: no age.

13. Antonucci LA, Penzel N, Sanfelici R, et al. Using combined environmental-clinical classification models to predict role functioning outcome in clinical high-risk states for psychosis and recent-onset depression. *British Journal of Psychiatry*.1-17. RAYYAN-INCLUSION: {"Sjur"=>"Included", "Wenche ten Velden"=>"Included"} | RAYYAN-INCLUSION: {"Sjur"=>"Excluded", "Wenche ten Velden"=>"Excluded"} | RAYYAN-EXCLUSION-REASONS: wrong outcome.

14. Arabghol F, Derakhshanpour F, Davari Ashtiyani R, Chimeh N, Panaghi L. Identification and Evaluation of Abused Children at Imam Hossein Hospital. *International Journal of High Risk Behaviors & Addiction*. 5(1):e27531. RAYYAN-INCLUSION: {"Sjur"=>"Included", "Wenche ten Velden"=>"Included"} | RAYYAN-INCLUSION: {"Sjur"=>"Excluded", "Wenche ten Velden"=>"Excluded"} | RAYYAN-EXCLUSION-REASONS: wrong outcome.

15. Arm, o M, ini C, et al. Coping Strategies Mediate the Effect of Stressful Life Events on Schizotypal Traits and Psychotic Symptoms in 22q11.2 Deletion Syndrome. *Schizophrenia Bulletin*. 44:S525-S535. RAYYAN-INCLUSION: {"Sjur"=>"Included", "Wenche ten Velden"=>"Included"} | RAYYAN-INCLUSION: {"Sjur"=>"Excluded", "Wenche ten Velden"=>"Excluded"} | RAYYAN-LABELS: uknown age of exposure | RAYYAN-EXCLUSION-REASONS: wrong predictor.

16. Ayub M, Saeed K, Kingdon D, Naeem F. Rate and predictors of psychotic symptoms after Kashmir earthquake. *European Archives of Psychiatry and Clinical Neuroscience*. 265(6):471-481. RAYYAN-INCLUSION: {"Sjur"=>"Included", "Wenche ten Velden"=>"Included"} | RAYYAN-INCLUSION: {"Sjur"=>"Excluded", "Wenche ten Velden"=>"Excluded"} | RAYYAN-LABELS: uknown age of exposure | RAYYAN-EXCLUSION-REASONS: no age.

17. Barker V, Bois C, Neilson E, et al. Childhood adversity and hippocampal and amygdala volumes in a population at familial high risk of schizophrenia. *Schizophrenia Research*. 175(1):42-47. RAYYAN-INCLUSION: {"Sjur"=>"Included", "Wenche ten Velden"=>"Included"} | RAYYAN-INCLUSION: {"Sjur"=>"Excluded", "Wenche ten Velden"=>"Excluded"} | RAYYAN-EXCLUSION-REASONS: wrong outcome.

18. Barzilay R, Calkins ME, Moore TM, et al. Association between traumatic stress load, psychopathology, and cognition in the Philadelphia Neurodevelopmental Cohort. *Psychological Medicine*. 49(2):325-334. RAYYAN-INCLUSION: {"Sjur"=>"Included", "Wenche ten Velden"=>"Included"} | RAYYAN-INCLUSION: {"Sjur"=>"Excluded", "Wenche ten Velden"=>"Excluded"} | RAYYAN-EXCLUSION-REASONS: wrong predictor | USER-NOTES: {"Wenche ten Velden"=>["Ikke spesifisert alder"]}.

19. Barzilay R, Patrick A, Calkins ME, Moore TM, Gur RC, Gur RE. Association between early-life trauma and obsessive compulsive symptoms in community youth. *Depression and Anxiety*. 36(7):586-595. RAYYAN-INCLUSION: {"Sjur"=>"Included", "Wenche ten Velden"=>"Included"} | RAYYAN-INCLUSION: {"Sjur"=>"Excluded", "Wenche ten Velden"=>"Excluded"} | RAYYAN-EXCLUSION-REASONS: wrong outcome.

20. Beards S, Fisher HL, Gayer-Anderson C, et al. Threatening life events and difficulties and psychotic disorder. *Schizophrenia Bulletin*. 46(4):814-822. RAYYAN-INCLUSION: {"Sjur"=>"Included", "Wenche ten Velden"=>"Included"} | RAYYAN-INCLUSION: {"Sjur"=>"Excluded", "Wenche ten Velden"=>"Excluded"} | RAYYAN-LABELS: exposure above age of 18 | RAYYAN-EXCLUSION-REASONS: wrong predictor | USER-NOTES: {"Wenche ten Velden"=>["Alder ved traume"]}.

21. Bechdolf A, Thompson A, Nelson B, et al. Experience of trauma and conversion to psychosis in an ultra-high-risk (prodromal) group. *Acta Psychiatrica Scandinavica*. 121(5):377-384. RAYYAN-INCLUSION: {"Sjur"=>"Included", "Wenche ten Velden"=>"Included"} | RAYYAN-INCLUSION: {"Sjur"=>"Excluded", "Wenche ten Velden"=>"Excluded"} | RAYYAN-LABELS: uknown age of exposure | RAYYAN-EXCLUSION-REASONS: no age.

22. Bendall S, Alvarez-Jimenez M, Hulbert CA, McGorry PD, Jackson HJ. Childhood trauma increases the risk of post-traumatic stress disorder in response to first-episode psychosis. *Australian and New Zealand Journal of Psychiatry*. 46(1):35-39. RAYYAN-INCLUSION: {"Sjur"=>"Included", "Wenche ten Velden"=>"Included"} | RAYYAN-INCLUSION: {"Sjur"=>"Excluded", "Wenche ten Velden"=>"Excluded"} | RAYYAN-EXCLUSION-REASONS: wrong outcome.

23. Bendall S, Jackson HJ, Hulbert CA. What self-generated speech is externally misattributed in psychosis? Testing three cognitive models in a first-episode sample. *Schizophrenia Research*. 129(1):36-41. RAYYAN-INCLUSION: {"Sjur"=>"Included", "Wenche ten Velden"=>"Included"} | USER-NOTES: {"Sjur"=>["Finner i mål på traumer?"]} | RAYYAN-INCLUSION: {"Sjur"=>"Excluded", "Wenche ten Velden"=>"Excluded"} | RAYYAN-EXCLUSION-REASONS: wrong outcome.

24. Bennouna-Greene M, Bennouna-Greene V, Berna F, Defranoux L. History of abuse and neglect in patients with schizophrenia who have a history of violence. *Child Abuse & Neglect*. 35(5):329-332. RAYYAN-INCLUSION: {"Sjur"=>"Included", "Wenche ten Velden"=>"Included"} | RAYYAN-INCLUSION: {"Sjur"=>"Excluded", "Wenche ten Velden"=>"Excluded"} | RAYYAN-EXCLUSION-REASONS: wrong outcome.

25. Berkowski M, MacDonald DA. Childhood Trauma and the Development of Paranormal Beliefs. *Journal of Nervous and Mental Disease*. 202(4):305-312. RAYYAN-INCLUSION: {"Sjur"=>"Included", "Wenche ten Velden"=>"Included"} | RAYYAN-INCLUSION: {"Sjur"=>"Excluded", "Wenche ten Velden"=>"Excluded"} | RAYYAN-EXCLUSION-REASONS: wrong outcome.

26. Berthelot N, Paccalet T, Gilbert E, et al. Childhood abuse and neglect may induce deficits in cognitive precursors of psychosis in high-risk children. *Journal of Psychiatry & Neuroscience*. 40(5):336-343. RAYYAN-INCLUSION: {"Sjur"=>"Included", "Wenche ten Velden"=>"Included"} | RAYYAN-INCLUSION: {"Sjur"=>"Excluded", "Wenche ten Velden"=>"Excluded"} | RAYYAN-EXCLUSION-REASONS: wrong outcome.

27. Betz LT, Penzel N, Kambeitz-Ilankovic L, et al. General psychopathology links burden of recent life events and psychotic symptoms in a network approach. *NPJ Schizophrenia*. 6(1):40. RAYYAN-INCLUSION: {"Sjur"=>"Included", "Wenche ten Velden"=>"Included"} | RAYYAN-INCLUSION: {"Sjur"=>"Excluded", "Wenche ten Velden"=>"Excluded"} | RAYYAN-LABELS: CT as covariat | RAYYAN-EXCLUSION-REASONS: wrong predictor.

28. Bilgi MM, Taspinar S, Aksoy B, Oguz K, Coburn K, Gonul AS. The relationship between childhood trauma, emotion recognition, and irritability in schizophrenia patients. *Psychiatry Research*. 251:90-96. RAYYAN-INCLUSION: {"Sjur"=>"Included", "Wenche ten Velden"=>"Included"} | RAYYAN-INCLUSION: {"Sjur"=>"Excluded", "Wenche ten Velden"=>"Excluded"} | RAYYAN-EXCLUSION-REASONS: wrong outcome.

29. Binbay T, Drukker M, Elbi H, et al. Testing the psychosis continuum: Differential impact of genetic and nongenetic risk factors and comorbid psychopathology across the entire spectrum of psychosis. *Schizophrenia Bulletin*. 38(5):992-1002. RAYYAN-INCLUSION: {"Sjur"=>"Included", "Wenche ten Velden"=>"Included"} | RAYYAN-INCLUSION: {"Sjur"=>"Excluded", "Wenche ten Velden"=>"Excluded"} | RAYYAN-EXCLUSION-REASONS: wrong predictor.

30. Bipin M, Premkumar P, Das MK, Lau JY, Sumich AL, Kumari V. Pituitary volume in people with chronic schizophrenia: Clarifying the roles of serious violence and childhood maltreatment. *Psychiatry Research: Neuroimaging Vol 314 2021, ArtID 111323*. 314RAYYAN-INCLUSION: {"Sjur"=>"Included", "Wenche ten Velden"=>"Included"} | RAYYAN-INCLUSION: {"Sjur"=>"Excluded", "Wenche ten Velden"=>"Excluded"} | RAYYAN-EXCLUSION-REASONS: wrong outcome.

31. Bless JJ, Laroi F, Laloyaux J, et al. Do adverse life events at first onset of auditory verbal hallucinations influence subsequent voice characteristics? Results from an epidemiological study. *Psychiatry Research*. 261:232-236. RAYYAN-INCLUSION: {"Sjur"=>"Included", "Wenche ten Velden"=>"Included"} | RAYYAN-INCLUSION: {"Sjur"=>"Excluded", "Wenche ten Velden"=>"Excluded"} | RAYYAN-LABELS: exposure above age of 18 | RAYYAN-EXCLUSION-REASONS: wrong predictor.

32. Bois C, Johnstone EC, Whalley HC, et al. Childhood adversity and cortical thickness and surface area in a population at familial high risk of schizophrenia. *Psychological Medicine*. 2016;46(4):891-896. RAYYAN-INCLUSION: {"Sjur"=>"Included", "Wenche ten Velden"=>"Included"} | RAYYAN-INCLUSION: {"Sjur"=>"Excluded", "Wenche ten Velden"=>"Excluded"} | RAYYAN-EXCLUSION-REASONS: wrong outcome.

33. Bolhuis K, Steenkamp LR, Blanken LME, et al. Schizophrenia polygenic risk is associated with child mental health problems through early childhood adversity: evidence for a gene-environment correlation. *European Child & Adolescent Psychiatry*. 26:26. RAYYAN-INCLUSION: {"Sjur"=>"Included", "Wenche ten Velden"=>"Included"} | RAYYAN-INCLUSION: {"Sjur"=>"Excluded", "Wenche ten Velden"=>"Excluded"} | RAYYAN-EXCLUSION-REASONS: wrong outcome.

34. Bouhaddani Se, van Domburgh L, Schaefer B, Doreleijers TA, Veling W. Psychotic experiences and trauma predict persistence of psychosocial problems in adolescence. *European Child & Adolescent Psychiatry*. 28(12):1597-1606. RAYYAN-INCLUSION: {"Sjur"=>"Included", "Wenche ten Velden"=>"Included"} | RAYYAN-INCLUSION: {"Sjur"=>"Excluded", "Wenche ten Velden"=>"Excluded"} | RAYYAN-EXCLUSION-REASONS: wrong outcome.

35. Bourgeois C, Lecomte T, McDuff P, Daigneault I. Child sexual abuse and age at onset of psychotic disorders: A matched-cohort study. *The Canadian Journal of Psychiatry / La Revue canadienne de psychiatrie*. 2021-6 2021;66(6):569-576. doi:doi:https://dx.doi.org/10.1177/0706743720970853. RAYYAN-INCLUSION: {"Sjur"=>"Included"} | RAYYAN-INCLUSION: {"Sjur"=>"Excluded"} | RAYYAN-LABELS: Child Service Records. RAYYAN-INCLUSION: {"Sjur"=>"Included"} | RAYYAN-INCLUSION: {"Sjur"=>"Excluded"} | RAYYAN-LABELS: Child Service Records

36. Boyda D, McFeeters D, Dhingra K, Kelleher I. A Population-Based Analysis of Interpersonal Trauma, Psychosis, and Suicide: Evidence, Pathways, and Implications. *Journal of Interpersonal Violence*. 37(1):912-934. RAYYAN-INCLUSION: {"Sjur"=>"Included", "Wenche ten Velden"=>"Included"} | RAYYAN-INCLUSION: {"Sjur"=>"Excluded", "Wenche ten Velden"=>"Excluded"} | RAYYAN-EXCLUSION-REASONS: wrong outcome.

37. Boyer L, Godin O, Misdrahi D, et al. Childhood trauma, depression and negative symptoms are independently associated with impaired quality of life in schizophrenia. Results from the national FACE-SZ cohort. *Schizophrenia Research*. 185:173-181. RAYYAN-INCLUSION: {"Sjur"=>"Included", "Wenche ten Velden"=>"Included"} | RAYYAN-INCLUSION: {"Sjur"=>"Excluded", "Wenche ten Velden"=>"Excluded"} | RAYYAN-EXCLUSION-REASONS: wrong outcome.

38. Boyette LL, van Dam D, Meijer C, et al. Personality compensates for impaired quality of life and social functioning in patients with psychotic disorders who experienced traumatic events. *Schizophrenia Bulletin*. 40(6):1356-65. RAYYAN-INCLUSION: {"Sjur"=>"Included", "Wenche ten Velden"=>"Included"} | RAYYAN-INCLUSION: {"Sjur"=>"Excluded", "Wenche ten Velden"=>"Excluded"} | RAYYAN-EXCLUSION-REASONS: wrong outcome.

39. Brent BK. Increased risk of psychotic symptoms at 12 years in children who have been maltreated by adults or bullied by peers. *Evidence-Based Mental Health*. 14(3):65. RAYYAN-INCLUSION: {"Sjur"=>"Included", "Wenche ten Velden"=>"Included"} | RAYYAN-INCLUSION: {"Sjur"=>"Excluded", "Wenche ten Velden"=>"Excluded"} | RAYYAN-EXCLUSION-REASONS: wrong publication type.

40. Burns JK, Jhazbhay K, Esterhuizen T, Emsley R. Exposure to trauma and the clinical presentation of first-episode psychosis in South Africa. *Journal of Psychiatric Research*. 45(2):179-184. RAYYAN-INCLUSION: {"Sjur"=>"Included", "Wenche ten Velden"=>"Included"} | RAYYAN-INCLUSION: {"Sjur"=>"Excluded", "Wenche ten Velden"=>"Excluded"} | RAYYAN-LABELS: uknown age of exposure | RAYYAN-EXCLUSION-REASONS: wrong predictor | USER-NOTES: {"Wenche ten Velden"=>["Dessverre. Ingen aldersspesifikasjon. Men 11 av 54 hadde \"witnessed someone being killed\" #SouthAfrica"]}.

41. Butjosa A, Gomez-Benito J, Huerta-Ramos E, et al. Incidence of stressful life events and influence of sociodemographic and clinical variables on the onset of first-episode psychosis. *Psychiatry Research*. 245:108-115. RAYYAN-INCLUSION: {"Sjur"=>"Included", "Wenche ten Velden"=>"Included"} | RAYYAN-INCLUSION: {"Sjur"=>"Excluded", "Wenche ten Velden"=>"Excluded"} | RAYYAN-LABELS: exposure above age of 18 | RAYYAN-EXCLUSION-REASONS: wrong predictor | USER-NOTES: {"Wenche ten Velden"=>["ingen aldersspesifikasjon"]}.

42. Butjosa A, Usall J, Vila-Badia R, et al. Impact of traumatic life events on clinical variables of individuals with first-episode psychosis and healthy controls. *International Journal of Social Psychiatry*.12. RAYYAN-INCLUSION: {"Sjur"=>"Included", "Wenche ten Velden"=>"Included"} | RAYYAN-INCLUSION: {"Sjur"=>"Excluded", "Wenche ten Velden"=>"Excluded"} | RAYYAN-LABELS: uknown age of exposure | RAYYAN-EXCLUSION-REASONS: wrong predictor | USER-NOTES: {"Wenche ten Velden"=>["Age. Again. "]}.

43. Campodonico C, Berry K, Haddock G, Varese F. Protective Factors Associated With Post-traumatic Outcomes in Individuals With Experiences of Psychosis. *Frontiers in Psychiatry*. 12:11. RAYYAN-INCLUSION: {"Sjur"=>"Included", "Wenche ten Velden"=>"Included"} | RAYYAN-INCLUSION: {"Sjur"=>"Excluded", "Wenche ten Velden"=>"Excluded"} | RAYYAN-LABELS: uknown age of exposure | RAYYAN-EXCLUSION-REASONS: wrong predictor.

44. Cancel A, Comte M, Boutet C, et al. Childhood trauma and emotional processing circuits in schizophrenia: A functional connectivity study. *Schizophrenia Research*. 184:69-72. RAYYAN-INCLUSION: {"Sjur"=>"Included", "Wenche ten Velden"=>"Included"} | RAYYAN-INCLUSION: {"Sjur"=>"Excluded", "Wenche ten Velden"=>"Excluded"} | RAYYAN-EXCLUSION-REASONS: wrong outcome.

45. Carr S, Hardy A, Fornells-Ambrojo M. The Trauma and Life Events (TALE) checklist: Development of a tool for improving routine screening in people with psychosis. *European Journal of Psychotraumatology Vol 9(1), 2018, ArtID 1512265*. 2018;9(1)RAYYAN-INCLUSION: {"Sjur"=>"Included", "Wenche ten Velden"=>"Included"} | RAYYAN-INCLUSION: {"Sjur"=>"Excluded", "Wenche ten Velden"=>"Excluded"} | RAYYAN-LABELS: exposure above age of 18 | RAYYAN-EXCLUSION-REASONS: no age.

46. Carrilho CG, Cougo SS, Bombassaro T, et al. Early Trauma and Cognitive Functions of Patients With Schizophrenia. *Frontiers in Psychiatry*. 10:7. RAYYAN-INCLUSION: {"Sjur"=>"Included", "Wenche ten Velden"=>"Included"} | RAYYAN-EXCLUSION-REASONS: wrong outcome | RAYYAN-INCLUSION: {"Sjur"=>"Excluded", "Wenche ten Velden"=>"Excluded"} | RAYYAN-EXCLUSION-REASONS: wrong outcome.

47. Catone G, Marwaha S, Kuipers E, et al. Bullying victimisation and risk of psychotic phenomena: analyses of British national survey data. *The Lancet Psychiatry*. 2(7):618-624. RAYYAN-INCLUSION: {"Sjur"=>"Included", "Wenche ten Velden"=>"Included"} | RAYYAN-INCLUSION: {"Sjur"=>"Excluded", "Wenche ten Velden"=>"Excluded"} | RAYYAN-EXCLUSION-REASONS: no age | USER-NOTES: {"Wenche ten Velden"=>["No age"]}.

48. Chalker SA, Parrish EM, Cano M, et al. Childhood Trauma Associations With the Interpersonal Psychological Theory of Suicide and Social Cognitive Biases in Psychotic Disorders. *Journal of Nervous & Mental Disease*. 23:23. RAYYAN-INCLUSION: {"Sjur"=>"Included", "Wenche ten Velden"=>"Included"} | RAYYAN-INCLUSION: {"Sjur"=>"Excluded", "Wenche ten Velden"=>"Excluded"} | RAYYAN-EXCLUSION-REASONS: wrong outcome.

49. Chiesa M, Larsen-Paya M, Martino M, Trinchieri M. The relationship between childhood adversity, psychiatric disorder and clinical severity: results from a multi-centre study. *Psychoanalytic Psychotherapy*. 30:79-95. RAYYAN-INCLUSION: {"Sjur"=>"Included", "Wenche ten Velden"=>"Included"} | RAYYAN-INCLUSION: {"Sjur"=>"Excluded", "Wenche ten Velden"=>"Excluded"} | RAYYAN-EXCLUSION-REASONS: wrong outcome.

50. Church C, Andreassen OA, Lorentzen S, Melle I, Aas M. Childhood trauma and minimization/denial in people with and without a severe mental disorder. *Frontiers in Psychology Vol 8 2017, ArtID 1276*. 8RAYYAN-INCLUSION: {"Sjur"=>"Included", "Wenche ten Velden"=>"Included"} | RAYYAN-INCLUSION: {"Sjur"=>"Excluded", "Wenche ten Velden"=>"Excluded"} | RAYYAN-EXCLUSION-REASONS: wrong outcome.

51. Ciufolini S, Gayer-Anderson C, Fisher HL, et al. Cortisol awakening response is decreased in patients with first-episode psychosis and increased in healthy controls with a history of severe childhood abuse. *Schizophrenia Research*. 205:38-44. RAYYAN-INCLUSION: {"Sjur"=>"Included", "Wenche ten Velden"=>"Included"} | RAYYAN-INCLUSION: {"Sjur"=>"Excluded", "Wenche ten Velden"=>"Excluded"} | RAYYAN-EXCLUSION-REASONS: wrong outcome.

52. Cohen CI, Palekar N, Barker J, Ramirez PM. The Relationship Between Trauma and Clinical Outcome Variables Among Older Adults With Schizophrenia Spectrum Disorders. *American Journal of Geriatric Psychiatry*. 20(5):408-415. RAYYAN-INCLUSION: {"Sjur"=>"Included", "Wenche ten Velden"=>"Included"} | RAYYAN-INCLUSION: {"Sjur"=>"Excluded", "Wenche ten Velden"=>"Excluded"} | RAYYAN-LABELS: sample age above 18,uknown age of exposure | RAYYAN-EXCLUSION-REASONS: no age.

53. Collazzoni A, Laloyaux J, Larøi F. Examination of humiliation and past maladaptive family context in persecutory ideation: An exploratory study. *Comprehensive Psychiatry*. 2017;78:19-24. RAYYAN-INCLUSION: {"Sjur"=>"Included", "Wenche ten Velden"=>"Included"} | RAYYAN-INCLUSION: {"Sjur"=>"Excluded", "Wenche ten Velden"=>"Excluded"} | RAYYAN-EXCLUSION-REASONS: no age | USER-NOTES: {"Wenche ten Velden"=>["no age"]}.

54. Connor C, Birchwood M. Abuse and dysfunctional affiliations in childhood: An exploration of their impact on voice-hearers' appraisals of power and expressed emotion. *Psychosis: Psychological, Social and Integrative Approaches*. 4(1):19-31. RAYYAN-INCLUSION: {"Sjur"=>"Included", "Wenche ten Velden"=>"Included"} | RAYYAN-INCLUSION: {"Sjur"=>"Excluded", "Wenche ten Velden"=>"Excluded"} | RAYYAN-EXCLUSION-REASONS: wrong outcome | USER-NOTES: {"Wenche ten Velden"=>["under tvil- vpd er ikke direkte et psykosemål"]}.

55. Conus P, Cotton S, Schimmelmann BG, et al. Pretreatment and outcome correlates of past sexual and physical trauma in 118 bipolar I disorder patients with a first episode of psychotic mania. *Bipolar Disorders*. 12(3):244-252. RAYYAN-INCLUSION: {"Sjur"=>"Included", "Wenche ten Velden"=>"Included"} | RAYYAN-INCLUSION: {"Sjur"=>"Excluded", "Wenche ten Velden"=>"Excluded"} | RAYYAN-EXCLUSION-REASONS: wrong outcome.

56. Corstens D, Longden E. The origins of voices: Links between life history and voice hearing in a survey of 100 cases. *Psychosis: Psychological, Social and Integrative Approaches*. 5(3):270-285. RAYYAN-INCLUSION: {"Sjur"=>"Included", "Wenche ten Velden"=>"Included"} | RAYYAN-INCLUSION: {"Sjur"=>"Excluded", "Wenche ten Velden"=>"Excluded"} | RAYYAN-EXCLUSION-REASONS: wrong study design.

57. Cosgrave J, Purple RJ, Haines R, et al. Do environmental risk factors for the development of psychosis distribute differently across dimensionally assessed psychotic experiences? *Transl Psychiatry Psychiatry*. 11(1):226. RAYYAN-INCLUSION: {"Sjur"=>"Included", "Wenche ten Velden"=>"Included"} | RAYYAN-INCLUSION: {"Sjur"=>"Excluded", "Wenche ten Velden"=>"Excluded"} | RAYYAN-LABELS: uknown age of exposure | RAYYAN-EXCLUSION-REASONS: wrong predictor.

58. Cristofaro SL, Cleary SD, Wan CR, et al. Measuring trauma and stressful events in childhood and adolescence among patients with first-episode psychosis: Initial factor structure, reliability, and validity of the Trauma Experiences Checklist. *Psychiatry Research*. 210(2):618-625. RAYYAN-INCLUSION: {"Sjur"=>"Included", "Wenche ten Velden"=>"Included"} | RAYYAN-INCLUSION: {"Sjur"=>"Excluded", "Wenche ten Velden"=>"Excluded"} | RAYYAN-EXCLUSION-REASONS: wrong outcome.

59. Cui Y, Kim SW, Lee BJ, et al. Negative Schema and Rumination as Mediators of the Relationship Between Childhood Trauma and Recent Suicidal Ideation in Patients With Early Psychosis. *Journal of Clinical Psychiatry*. 80(3):02. RAYYAN-INCLUSION: {"Sjur"=>"Included", "Wenche ten Velden"=>"Included"} | RAYYAN-INCLUSION: {"Sjur"=>"Excluded", "Wenche ten Velden"=>"Excluded"} | RAYYAN-EXCLUSION-REASONS: wrong outcome.

60. Cullen AE, Addington J, Bearden CE, et al. Stressor-cortisol concordance among individuals at clinical high-risk for psychosis: Novel findings from the NAPLS cohort. *Psychoneuroendocrinology Vol 115 2020, ArtID 104649*. 115RAYYAN-INCLUSION: {"Sjur"=>"Included", "Wenche ten Velden"=>"Included"} | RAYYAN-INCLUSION: {"Sjur"=>"Excluded", "Wenche ten Velden"=>"Excluded"} | RAYYAN-EXCLUSION-REASONS: wrong outcome.

61. Cutajar MC, Mullen PE, Ogloff JR, Thomas SD, Wells DL, Spataro J. Schizophrenia and other psychotic disorders in a cohort of sexually abused children. *Archives of General Psychiatry*. 2010-11 2010;67(11):1114-9. doi:doi:https://dx.doi.org/10.1001/archgenpsychiatry.2010.147. RAYYAN-INCLUSION: {"Sjur"=>"Included"} | RAYYAN-INCLUSION: {"Sjur"=>"Excluded"} | RAYYAN-LABELS: Medical records. RAYYAN-INCLUSION: {"Sjur"=>"Included"} | RAYYAN-INCLUSION: {"Sjur"=>"Excluded"} | RAYYAN-LABELS: Medical records

62. Cutajar MC, Mullen PE, Ogloff JR, Thomas SD, Wells DL, Spataro J. Psychopathology in a large cohort of sexually abused children followed up to 43 years. *Child Abuse & Neglect*. 2010-11 2010;34(11):813-22. doi:doi:https://dx.doi.org/10.1016/j.chiabu.2010.04.004. RAYYAN-INCLUSION: {"Sjur"=>"Included"} | RAYYAN-INCLUSION: {"Sjur"=>"Excluded"} | RAYYAN-LABELS: Medical records. RAYYAN-INCLUSION: {"Sjur"=>"Included"} | RAYYAN-INCLUSION: {"Sjur"=>"Excluded"} | RAYYAN-LABELS: Medical records

63. Cyran A, Piotrowski P, Samochowiec J, Grazlewski T, Misiak B. Risk factors of deficit and non-deficit schizophrenia: Results from a cross-sectional study. *Revista De Psiquiatria Y Salud Mental*. 2022-10 2022;15(4):223-229. doi:doi:https://dx.doi.org/10.1016/j.rpsmen.2022.05.001. RAYYAN-INCLUSION: {"Sjur"=>"Included"} | RAYYAN-INCLUSION: {"Sjur"=>"Excluded"} | RAYYAN-EXCLUSION-REASONS: wrong outcome. RAYYAN-INCLUSION: {"Sjur"=>"Included"} | RAYYAN-INCLUSION: {"Sjur"=>"Excluded"} | RAYYAN-EXCLUSION-REASONS: wrong outcome

64. De Fazio P, Pugliese V, Cattolico M, Aloi M, Segura-Garcia C. The relationship between childhood trauma and aberrant salience: A preliminary study in patients with schizophrenia. *Journal of Psychopathology*. 26(1):28-35. RAYYAN-INCLUSION: {"Sjur"=>"Included", "Wenche ten Velden"=>"Included"} | RAYYAN-INCLUSION: {"Sjur"=>"Excluded", "Wenche ten Velden"=>"Excluded"} | RAYYAN-EXCLUSION-REASONS: wrong outcome.

65. De-Nardin EMS, Muratori CA, Ribeiro IS, Huguete RB, Salgado JV. Childhood trauma is associated with onset of symptoms, functioning and cognition in patients with schizophrenia. *Trends in Psychiatry & Psychotherapy*. 8:08. RAYYAN-INCLUSION: {"Sjur"=>"Included", "Wenche ten Velden"=>"Included"} | RAYYAN-INCLUSION: {"Sjur"=>"Excluded", "Wenche ten Velden"=>"Excluded"} | RAYYAN-EXCLUSION-REASONS: wrong outcome.

66. Debost J-C, Petersen L, Grove J, et al. Investigating interactions between early life stress and two single nucleotide polymorphisms in HSD11B2 on the risk of schizophrenia. *Psychoneuroendocrinology*. 60:18-27. RAYYAN-INCLUSION: {"Sjur"=>"Included", "Wenche ten Velden"=>"Included"} | RAYYAN-INCLUSION: {"Sjur"=>"Excluded", "Wenche ten Velden"=>"Excluded"} | RAYYAN-LABELS: Wrong measure,wrong independent variable | RAYYAN-EXCLUSION-REASONS: wrong predictor.

67. Dugre JR, Guay J-P, Dumais A, re. Risk factors of compliance with self-harm command hallucinations in individuals with affective and non-affective psychosis. *Schizophrenia Research*. 195:115-121. RAYYAN-INCLUSION: {"Sjur"=>"Included", "Wenche ten Velden"=>"Included"} | RAYYAN-INCLUSION: {"Sjur"=>"Excluded", "Wenche ten Velden"=>"Excluded"} | RAYYAN-EXCLUSION-REASONS: wrong outcome.

68. Elklit A, Shevlin M. Female Sexual Victimization Predicts Psychosis: A Case-Control Study Based on the Danish Registry System. *Schizophrenia Bulletin*. 37(6):1305-1310. RAYYAN-INCLUSION: {"Sjur"=>"Included", "Wenche ten Velden"=>"Included"} | RAYYAN-INCLUSION: {"Sjur"=>"Excluded", "Wenche ten Velden"=>"Excluded"} | RAYYAN-LABELS: uknown age of exposure | RAYYAN-EXCLUSION-REASONS: no age.

69. Ellenbogen S, Colin-Vezina D, Sinha V, na, Chabot M, Wells SJ. Contrasting mental health correlates of physical and sexual abuse-related shame. *Journal of Child and Adolescent Mental Health*. 30(2):87-97. RAYYAN-INCLUSION: {"Sjur"=>"Included", "Wenche ten Velden"=>"Included"} | RAYYAN-INCLUSION: {"Sjur"=>"Excluded", "Wenche ten Velden"=>"Excluded"} | RAYYAN-LABELS: Wrong measure,wrong independent variable | RAYYAN-EXCLUSION-REASONS: wrong predictor.

70. Ered A, Gibson LE, Maxwell SD, Cooper S, Ellman LM. Coping as a mediator of stress and psychotic-like experiences. *European Psychiatry*. 43:9-13. RAYYAN-INCLUSION: {"Sjur"=>"Included", "Wenche ten Velden"=>"Included"} | RAYYAN-INCLUSION: {"Sjur"=>"Excluded", "Wenche ten Velden"=>"Excluded"} | RAYYAN-LABELS: uknown age of exposure | RAYYAN-EXCLUSION-REASONS: no age.

71. Evren C, Sar V, Dalbudak E, et al. Childhood trauma and psychopathology among alcohol-dependent men: No interaction with temperament and character. *Psychopathology*. 44(1):34-39. doi:doi:. RAYYAN-INCLUSION: {"Sjur"=>"Included", "Wenche ten Velden"=>"Included"} | RAYYAN-INCLUSION: {"Sjur"=>"Excluded", "Wenche ten Velden"=>"Included"} | RAYYAN-EXCLUSION-REASONS: wrong outcome. RAYYAN-INCLUSION: {"Sjur"=>"Included", "Wenche ten Velden"=>"Included"} | RAYYAN-INCLUSION: {"Sjur"=>"Excluded", "Wenche ten Velden"=>"Included"} | RAYYAN-EXCLUSION-REASONS: wrong outcome

72. Faravelli C, Amedei S, Rotella F, et al. Childhood traumata, Dexamethasone Suppression Test and psychiatric symptoms: A trans-diagnostic approach. *Psychological Medicine*. 40(12):2037-2048. RAYYAN-INCLUSION: {"Sjur"=>"Included", "Wenche ten Velden"=>"Included"} | RAYYAN-INCLUSION: {"Jone"=>"Excluded", "Sjur"=>"Excluded"} | RAYYAN-EXCLUSION-REASONS: wrong outcome.

73. Fekih-Romdhane F, Nsibi T, Sassi H, Cheour M. Link between childhood trauma and psychotic-like experiences in non-affected siblings of schizophrenia patients: A case-control study. *Early intervention in psychiatry*. 15(5):1154-1166. RAYYAN-INCLUSION: {"Sjur"=>"Included", "Wenche ten Velden"=>"Included"} | RAYYAN-INCLUSION: {"Jone"=>"Excluded", "Sjur"=>"Excluded"} | RAYYAN-EXCLUSION-REASONS: wrong population.

74. Ferreira F, Castro D, Araujo AS, Fonseca AR, Ferreira TB. Exposure to Traumatic Events and Development of Psychotic Symptoms in a Prison Population: A Network Analysis Approach. *Psychiatry Research*. 286:8. RAYYAN-INCLUSION: {"Sjur"=>"Included", "Wenche ten Velden"=>"Included"} | RAYYAN-INCLUSION: {"Jone"=>"Excluded", "Sjur"=>"Excluded"} | RAYYAN-LABELS: uknown age of exposure | RAYYAN-EXCLUSION-REASONS: no age.

75. Fisher HL, Craig TK, Fearon P, et al. Reliability and comparability of psychosis patients' retrospective reports of childhood abuse. *Schizophrenia Bulletin*. 37(3):546-553. RAYYAN-INCLUSION: {"Sjur"=>"Included", "Wenche ten Velden"=>"Included"} | RAYYAN-INCLUSION: {"Sjur"=>"Excluded"} | RAYYAN-EXCLUSION-REASONS: wrong study design.

76. Fisher HL, McGuffin P, Boydell J, et al. Interplay between childhood physical abuse and familial risk in the onset of psychotic disorders. *Schizophrenia Bulletin*. 40(6):1443-1451. RAYYAN-INCLUSION: {"Sjur"=>"Included", "Wenche ten Velden"=>"Included"} | RAYYAN-INCLUSION: {"Sjur"=>"Excluded"} | RAYYAN-EXCLUSION-REASONS: wrong outcome.

77. Fitzhenry M, Harte E, Carr A, et al. Child maltreatment and adult psychopathology in an Irish context. *Child Abuse & Neglect*. 45:101-107. RAYYAN-INCLUSION: {"Sjur"=>"Included", "Wenche ten Velden"=>"Included"} | RAYYAN-INCLUSION: {"Jone"=>"Excluded", "Sjur"=>"Excluded"} | RAYYAN-EXCLUSION-REASONS: wrong outcome.

78. Frissen A, Lieverse R, Marcelis M, Drukker M, Delespaul P. Psychotic disorder and educational achievement: A family-based analysis. *Social Psychiatry and Psychiatric Epidemiology: The International Journal for Research in Social and Genetic Epidemiology and Mental Health Services*. 50(10):1511-1518. RAYYAN-INCLUSION: {"Sjur"=>"Included", "Wenche ten Velden"=>"Included"} | RAYYAN-INCLUSION: {"Sjur"=>"Excluded"} | RAYYAN-EXCLUSION-REASONS: wrong outcome.

79. Frost R, Louison Vang M, Karatzias T, Hyl, Philip, Shevlin M. The distribution of psychosis, ICD-11 PTSD and complex PTSD symptoms among a trauma-exposed UK general population sample. *Psychosis: Psychological, Social and Integrative Approaches*. 11(3):187-198. RAYYAN-INCLUSION: {"Sjur"=>"Included", "Wenche ten Velden"=>"Included"} | RAYYAN-INCLUSION: {"Sjur"=>"Excluded"} | RAYYAN-EXCLUSION-REASONS: wrong outcome.

80. Frydecka D, Kotowicz K, Gaweda L, et al. Effects of interactions between variation in dopaminergic genes, traumatic life events, and anomalous self-experiences on psychosis proneness: Results from a cross-sectional study in a nonclinical sample. *European Psychiatry: the Journal of the Association of European Psychiatrists*. 63(1):e104. RAYYAN-INCLUSION: {"Sjur"=>"Included", "Wenche ten Velden"=>"Included"} | RAYYAN-INCLUSION: {"Sjur"=>"Excluded"} | RAYYAN-LABELS: uknown age of exposure | RAYYAN-EXCLUSION-REASONS: no age.

81. Frydecka D, Kotowicz K, Gaweda L, et al. Effects of interactions between variation in dopaminergic genes, traumatic life events, and anomalous self-experiences on psychosis proneness: Results from a cross-sectional study in a nonclinical sample. *European Psychiatry Vol 63, 2020, ArtID e104*. 2020-11 2020;63doi:doi:https://dx.doi.org/10.1192/j.eurpsy.2020.103. RAYYAN-INCLUSION: {"Sjur"=>"Included"} | RAYYAN-INCLUSION: {"Sjur"=>"Excluded"} | RAYYAN-LABELS: Unknown age. RAYYAN-INCLUSION: {"Sjur"=>"Included"} | RAYYAN-INCLUSION: {"Sjur"=>"Excluded"} | RAYYAN-LABELS: Unknown age

82. Fung HW, Chan C, Ross CA. Clinical correlates of hearing voices among people seeking interventions for dissociation: a cross-cultural investigation. *Psychosis-Psychological Social and Integrative Approaches*. 12(4):328-338. RAYYAN-INCLUSION: {"Sjur"=>"Included", "Wenche ten Velden"=>"Included"} | RAYYAN-INCLUSION: {"Sjur"=>"Excluded"} | RAYYAN-LABELS: uknown age of exposure,both child and adult exposure in one measure | RAYYAN-EXCLUSION-REASONS: no age.

83. Fung HW, Liu RKW, Ma YHE. Hearing voices and its psychosocial correlates in four Chinese samples. *Psychosis-Psychological Social and Integrative Approaches*. 11(2):162-173. RAYYAN-INCLUSION: {"Sjur"=>"Included", "Wenche ten Velden"=>"Included"} | RAYYAN-INCLUSION: {"Jone"=>"Excluded", "Sjur"=>"Excluded"} | RAYYAN-LABELS: uknown age of exposure,both child and adult exposure in one measure | RAYYAN-EXCLUSION-REASONS: wrong population.

84. Fusar-Poli P, Sunkel C, Larrauri CA, et al. Violence and schizophrenia: the role of social determinants of health and the need for early intervention. *World Psychiatry*. 2023 2023;22(2):230-231. doi:doi:10.1002/wps.21074. RAYYAN-INCLUSION: {"Sjur"=>"Included"} | RAYYAN-INCLUSION: {"Sjur"=>"Excluded"} | RAYYAN-EXCLUSION-REASONS: wrong publication type. RAYYAN-INCLUSION: {"Sjur"=>"Included"} | RAYYAN-INCLUSION: {"Sjur"=>"Excluded"} | RAYYAN-EXCLUSION-REASONS: wrong publication type

85. Gatov E, Koziel N, Kurdyak P, et al. Discharge and post-discharge outcomes of psychiatric inpatients with a lifetime history of exposure to interpersonal trauma: A population-based study. *General Hospital Psychiatry*. 65:82-90. RAYYAN-INCLUSION: {"Jone"=>"Included", "Sjur"=>"Included"} | RAYYAN-INCLUSION: {"Sjur"=>"Excluded"} | RAYYAN-EXCLUSION-REASONS: wrong outcome.

86. Gaweda L, Pionke R, Krezolek M, Frydecka D, Nelson B, Cechnicki A. The interplay between childhood trauma, cognitive biases, psychotic-like experiences and depression and their additive impact on predicting lifetime suicidal behavior in young adults. *Psychological Medicine*. 50(1):116-124. RAYYAN-INCLUSION: {"Jone"=>"Included", "Sjur"=>"Included"} | RAYYAN-INCLUSION: {"Jone"=>"Excluded", "Sjur"=>"Excluded"} | RAYYAN-EXCLUSION-REASONS: wrong outcome.

87. Gaweda L, Pionke R, Krezolek M, et al. Self-disturbances, cognitive biases and insecure attachment as mechanisms of the relationship between traumatic life events and psychotic-like experiences in non-clinical adults - A path analysis. *Psychiatry Research*. 259:571-578. RAYYAN-INCLUSION: {"Jone"=>"Included", "Sjur"=>"Included"} | RAYYAN-INCLUSION: {"Jone"=>"Excluded", "Sjur"=>"Excluded"} | RAYYAN-LABELS: uknown age of exposure | RAYYAN-EXCLUSION-REASONS: wrong population.

88. Gaweda L, Prochwicz K, Adamczyk P, et al. The role of self-disturbances and cognitive biases in the relationship between traumatic life events and psychosis proneness in a non-clinical sample. *Schizophrenia Research*. 193:218-224. RAYYAN-INCLUSION: {"Jone"=>"Included", "Sjur"=>"Included"} | RAYYAN-INCLUSION: {"Jone"=>"Excluded", "Sjur"=>"Excluded"} | RAYYAN-LABELS: uknown age of exposure | RAYYAN-EXCLUSION-REASONS: wrong population.

89. Gayer-Anderson C, Reininghaus U, Paetzold I, et al. A comparison between self-report and interviewer-rated retrospective reports of childhood abuse among individuals with first-episode psychosis and population-based controls. *Journal of Psychiatric Research*. 123:145-150. RAYYAN-INCLUSION: {"Jone"=>"Included", "Sjur"=>"Included"} | RAYYAN-INCLUSION: {"Jone"=>"Excluded", "Sjur"=>"Excluded"} | RAYYAN-EXCLUSION-REASONS: wrong study design.

90. Gayer-Anderson C, Reininghaus U, Paetzold I, et al. A comparison between self-report and interviewer-rated retrospective reports of childhood abuse among individuals with first-episode psychosis and population-based controls. *Journal of Psychiatric Research*. 2020-11-4 2020;123:145-150. doi:doi:https://dx.doi.org/10.1016/j.jpsychires.2020.02.002. RAYYAN-INCLUSION: {"Sjur"=>"Included"} | RAYYAN-INCLUSION: {"Sjur"=>"Excluded"} | RAYYAN-EXCLUSION-REASONS: wrong outcome. RAYYAN-INCLUSION: {"Sjur"=>"Included"} | RAYYAN-INCLUSION: {"Sjur"=>"Excluded"} | RAYYAN-EXCLUSION-REASONS: wrong outcome

91. Geddes G, Ehlers A, Freeman D. Hallucinations in the months after a trauma: An investigation of the role of cognitive processing of a physical assault in the occurrence of hallucinatory experiences. *Psychiatry Research*. 246:601-605. RAYYAN-INCLUSION: {"Jone"=>"Included", "Sjur"=>"Included"} | RAYYAN-INCLUSION: {"Sjur"=>"Excluded"} | RAYYAN-LABELS: wrong independent variable | RAYYAN-EXCLUSION-REASONS: wrong predictor.

92. Geoffrion S, Nolet K, Giguere CE, et al. Psychosocial Profiles of Patients Admitted to Psychiatric Emergency Services: Results from the Signature Biobank Project: Profils Psychosociaux de Patients Admis Dans des Services d'Urgence Psychiatrique : Resultats du Projet Banque Signature. *Canadian Journal of Psychiatry - Revue Canadienne de Psychiatrie*.7067437211018793. RAYYAN-INCLUSION: {"Jone"=>"Included", "Sjur"=>"Included"} | RAYYAN-INCLUSION: {"Jone"=>"Excluded", "Sjur"=>"Excluded"} | RAYYAN-EXCLUSION-REASONS: wrong population.

93. Gibson LE, Anglin DM, Klugman JT, et al. Stress sensitivity mediates the relationship between traumatic life events and attenuated positive psychotic symptoms differentially by gender in a,college population sample. *Journal of Psychiatric Research*. 53:111-118. RAYYAN-INCLUSION: {"Jone"=>"Included", "Sjur"=>"Included"} | USER-NOTES: {"Sjur"=>["Alder for utsatthet?"]} | RAYYAN-INCLUSION: {"Jone"=>"Excluded", "Sjur"=>"Excluded"} | RAYYAN-LABELS: uknown age of exposure | RAYYAN-EXCLUSION-REASONS: wrong population.

94. Gibson LE, Cooper S, Reeves LE, Anglin DM, Ellman LM. The association between traumatic life events and psychological symptoms from a conservative, transdiagnostic perspective. *Psychiatry Research*. 252:70-74. RAYYAN-INCLUSION: {"Jone"=>"Included", "Sjur"=>"Included"} | RAYYAN-INCLUSION: {"Sjur"=>"Excluded"} | RAYYAN-LABELS: uknown age of exposure | RAYYAN-EXCLUSION-REASONS: no age.

95. Gibson LE, Reeves LE, Cooper S, Olino TM, Ellman LM. Traumatic life event exposure and psychotic-like experiences: A multiple mediation model of cognitive-based mechanisms. *Schizophrenia Research*. 205:15-22. RAYYAN-INCLUSION: {"Jone"=>"Included", "Sjur"=>"Included"} | RAYYAN-INCLUSION: {"Sjur"=>"Excluded"} | RAYYAN-LABELS: uknown age of exposure | RAYYAN-EXCLUSION-REASONS: no age.

96. Gomez JM. Does Gender Matter? An Exploratory Study of Cultural Betrayal Trauma and Hallucinations in Latino Undergraduates at a Predominantly White University. *Journal of Interpersonal Violence*. 36(3):NP1375-NP1390. RAYYAN-INCLUSION: {"Jone"=>"Included", "Sjur"=>"Included"} | RAYYAN-INCLUSION: {"Jone"=>"Excluded", "Sjur"=>"Excluded"} | RAYYAN-LABELS: uknown age of exposure | RAYYAN-EXCLUSION-REASONS: wrong population.

97. Gomez JM, Freyd JJ. High betrayal child sexual abuse and hallucinations: A test of an indirect effect of dissociation. *Journal of Child Sexual Abuse: Research, Treatment, & Program Innovations for Victims, Survivors, & Offenders*. 26(5):507-518. RAYYAN-INCLUSION: {"Jone"=>"Included", "Sjur"=>"Included"} | RAYYAN-INCLUSION: {"Jone"=>"Excluded", "Sjur"=>"Excluded"} | RAYYAN-LABELS: uknown age of exposure | RAYYAN-EXCLUSION-REASONS: wrong population.

98. Gottlieb JD, Mueser KT, Rosenberg SD, Xie HY, Wofe RS. Psychotic depression, posttraumatic stress disorder, and engagement in cognitive-behavioral therapy within an outpatient sample of adults with serious mental illness. *Comprehensive Psychiatry*. 52(1):41-49. RAYYAN-INCLUSION: {"Jone"=>"Included", "Sjur"=>"Included"} | RAYYAN-INCLUSION: {"Sjur"=>"Excluded"} | RAYYAN-LABELS: wrong independent variable | RAYYAN-EXCLUSION-REASONS: wrong predictor.

99. Grattan RE, Lara N, Botello RM, et al. A History of Trauma is Associated with Aggression, Depression, Non-Suicidal Self-Injury Behavior, and Suicide Ideation in First-Episode Psychosis. *Journal of Clinical Medicine*. 8(7):17. RAYYAN-INCLUSION: {"Jone"=>"Included", "Sjur"=>"Included"} | RAYYAN-INCLUSION: {"Sjur"=>"Excluded"} | RAYYAN-EXCLUSION-REASONS: wrong outcome.

100. Groenewold NA. Complex PTSD as mediator of psychosis after childhood trauma. *The Lancet Psychiatry*. 2023-11-10 2023;10(10):735-736. doi:doi:https://dx.doi.org/10.1016/S2215-0366(23)00302-4. RAYYAN-INCLUSION: {"Sjur"=>"Included"} | RAYYAN-INCLUSION: {"Sjur"=>"Excluded"} | RAYYAN-EXCLUSION-REASONS: wrong publication type. RAYYAN-INCLUSION: {"Sjur"=>"Included"} | RAYYAN-INCLUSION: {"Sjur"=>"Excluded"} | RAYYAN-EXCLUSION-REASONS: wrong publication type

101. Gunter TD, Chibnall JT, Antoniak SK, McCormick B, Black DW. Relative Contributions of Gender and Traumatic Life Experience to the Prediction of Mental Disorders in a Sample of Incarcerated Offenders. *Behavioral Sciences & the Law*. 30(5):615-630. RAYYAN-INCLUSION: {"Jone"=>"Included", "Sjur"=>"Included"} | RAYYAN-INCLUSION: {"Jone"=>"Excluded", "Sjur"=>"Excluded"} | RAYYAN-LABELS: uknown age of exposure | RAYYAN-EXCLUSION-REASONS: wrong population.

102. Gur RE, Moore TM, Rosen AF, et al. Burden of environmental adversity associated with psychopathology, maturation, and brain behavior parameters in youths. *JAMA Psychiatry*. 76(9):966-975. RAYYAN-INCLUSION: {"Jone"=>"Included", "Sjur"=>"Included"} | RAYYAN-INCLUSION: {"Jone"=>"Excluded", "Sjur"=>"Excluded"} | RAYYAN-LABELS: uknown age of exposure | RAYYAN-EXCLUSION-REASONS: wrong population.

103. Hachtel H, Fullam R, Malone A, Murphy BP, Huber C, Carroll A. Victimization, violence and facial affect recognition in a community sample of first-episode psychosis patients. *Early Intervention in Psychiatry*. 14(3):283-292. RAYYAN-INCLUSION: {"Jone"=>"Included", "Sjur"=>"Included"} | RAYYAN-INCLUSION: {"Sjur"=>"Excluded"} | RAYYAN-EXCLUSION-REASONS: wrong outcome.

104. Hardy A, O'Driscoll C, Steel C, van der Gaag M, van den Berg D. A network analysis of post-traumatic stress and psychosis symptoms. *Psychological Medicine*. 51(14):2485-2492. RAYYAN-INCLUSION: {"Jone"=>"Included", "Sjur"=>"Included"} | RAYYAN-INCLUSION: {"Sjur"=>"Excluded"} | RAYYAN-LABELS: wrong independent variable | RAYYAN-EXCLUSION-REASONS: wrong predictor.

105. Hassan AN, De Luca V. The effect of lifetime adversities on resistance to antipsychotic treatment in schizophrenia patients. *Schizophrenia Research*. 161(2):496-500. RAYYAN-INCLUSION: {"Jone"=>"Included", "Sjur"=>"Included"} | RAYYAN-INCLUSION: {"Sjur"=>"Excluded"} | RAYYAN-EXCLUSION-REASONS: wrong outcome.

106. Haug E, Oie M, Andreassen OA, et al. High levels of anomalous self-experience are associated with longer duration of untreated psychosis. *Early Intervention in Psychiatry*. 11(2):133-138. RAYYAN-INCLUSION: {"Jone"=>"Included", "Sjur"=>"Included"} | RAYYAN-INCLUSION: {"Sjur"=>"Excluded"} | RAYYAN-EXCLUSION-REASONS: wrong outcome.

107. Hazelgrove K, Biaggi A, ra, et al. Risk factors for postpartum relapse in women at risk of postpartum psychosis: The role of psychosocial stress and the biological stress system. *Psychoneuroendocrinology Vol 128 2021, ArtID 105218*. 128RAYYAN-INCLUSION: {"Jone"=>"Included", "Sjur"=>"Included"} | RAYYAN-INCLUSION: {"Sjur"=>"Excluded"} | RAYYAN-EXCLUSION-REASONS: wrong outcome.

108. Heinonen E, Knekt P, Harkanen T, Virtala E, Lindfors O. Childhood adversities as predictors of improvement in psychiatric symptoms and global functioning in solution-focused and short- and long-term psychodynamic psychotherapy during a 5-year follow-up. *Journal of Affective Disorders*. 235:525-534. RAYYAN-INCLUSION: {"Jone"=>"Included", "Sjur"=>"Included"} | RAYYAN-INCLUSION: {"Jone"=>"Excluded", "Sjur"=>"Excluded"} | RAYYAN-EXCLUSION-REASONS: wrong outcome.

109. Herms EN, Bolbecker AR, Wisner KM. Impaired Sleep Mediates the Relationship Between Interpersonal Trauma and Subtypes of Delusional Ideation. *Schizophrenia Bulletin*. 2023-6 2023:11. doi:doi:10.1093/schbul/sbad081. RAYYAN-INCLUSION: {"Sjur"=>"Included"} | RAYYAN-INCLUSION: {"Sjur"=>"Excluded"} | RAYYAN-LABELS: Unknown age. RAYYAN-INCLUSION: {"Sjur"=>"Included"} | RAYYAN-INCLUSION: {"Sjur"=>"Excluded"} | RAYYAN-LABELS: Unknown age

110. Ho GWK, Hyl, P., Karatzias T, Bressington D, Shevlin M. Traumatic life events as risk factors for psychosis and ICD-11 complex PTSD: a gender-specific examination. *European Journal of Psychotraumatology*. 12(1):10. RAYYAN-INCLUSION: {"Jone"=>"Included", "Sjur"=>"Included"} | RAYYAN-INCLUSION: {"Sjur"=>"Excluded"} | RAYYAN-LABELS: uknown age of exposure | RAYYAN-EXCLUSION-REASONS: no age.

111. Hodann-Caudevilla RM, Garcia JJM, Julian FAB. Childhood trauma and personal recovery in schizophrenia: Mediating role of experiential avoidance and insecure attachment. *Clinical Schizophrenia and Related Psychoses*. 2021;15(2)RAYYAN-INCLUSION: {"Jone"=>"Included", "Sjur"=>"Included"} | RAYYAN-INCLUSION: {"Sjur"=>"Excluded"} | RAYYAN-EXCLUSION-REASONS: wrong outcome.

112. Hodgekins J, Lower R, Wilson J, et al. Clinician‐rated and self‐reported psychotic‐like experiences in individuals accessing a specialist Youth Mental Health Service. *British Journal of Clinical Psychology*. 2018;57(3):367-381. RAYYAN-INCLUSION: {"Jone"=>"Included", "Sjur"=>"Included"} | RAYYAN-INCLUSION: {"Sjur"=>"Excluded"} | RAYYAN-LABELS: uknown age of exposure | RAYYAN-EXCLUSION-REASONS: no age.

113. Jiang MG, Shao X, Zhang Y, et al. Family relationships and personality disorder functioning styles in paranoid schizophrenia. *Personality and Individual Differences*. 169:5. RAYYAN-INCLUSION: {"Jone"=>"Included", "Sjur"=>"Included"} | RAYYAN-INCLUSION: {"Sjur"=>"Excluded"} | RAYYAN-EXCLUSION-REASONS: wrong outcome.

114. Jones N, Godzikovskaya J, Zhao Z, Vasquez A, Gilbert A, Davidson L. Intersecting disadvantage: Unpacking poor outcomes within early intervention in psychosis services. *Early Intervention in Psychiatry*. 13(3):488-494. RAYYAN-INCLUSION: {"Jone"=>"Included", "Sjur"=>"Included"} | RAYYAN-INCLUSION: {"Sjur"=>"Excluded"} | RAYYAN-EXCLUSION-REASONS: wrong outcome.

115. Justo A, Risso A, Moskowitz A, Gonzalez A. Schizophrenia and dissociation: Its relation with severity, self-esteem and awareness of illness. *Schizophrenia Research*. 197:170-175. RAYYAN-INCLUSION: {"Jone"=>"Included", "Sjur"=>"Included"} | RAYYAN-INCLUSION: {"Sjur"=>"Excluded"} | RAYYAN-LABELS: uknown age of exposure | RAYYAN-EXCLUSION-REASONS: no age.

116. Kilian C, Supanya S, Probst C, et al. Traumatic events and psychotic experiences: a nationally representative study in Thailand. *Epidemiology and Psychiatric Sciences*. 30:8. RAYYAN-INCLUSION: {"Jone"=>"Included", "Sjur"=>"Included"} | RAYYAN-INCLUSION: {"Sjur"=>"Excluded"} | RAYYAN-LABELS: uknown age of exposure | RAYYAN-EXCLUSION-REASONS: no age.

117. Kilian S, Asmal L, Chiliza B, et al. Childhood adversity and cognitive function in schizophrenia spectrum disorders and healthy controls: Evidence for an association between neglect and social cognition. *Psychological Medicine*. 48(13):2186-2193. RAYYAN-INCLUSION: {"Jone"=>"Included", "Sjur"=>"Included"} | RAYYAN-INCLUSION: {"Sjur"=>"Excluded"} | RAYYAN-EXCLUSION-REASONS: wrong outcome.

118. Kilian S, Asmal L, Phahladira L, et al. The association between childhood trauma and treatment outcomes in schizophrenia spectrum disorders. *Psychiatry Research*. 289:113004. RAYYAN-INCLUSION: {"Jone"=>"Included", "Sjur"=>"Included"} | RAYYAN-INCLUSION: {"Sjur"=>"Excluded"} | RAYYAN-EXCLUSION-REASONS: wrong outcome.

119. Kingdon DG, Ashcroft K, Bh, et al. Schizophrenia and borderline personality disorder: Similarities and differences in the experience of auditory hallucinations, paranoia, and childhood trauma. *Journal of Nervous and Mental Disease*. 198(6):399-403. RAYYAN-INCLUSION: {"Jone"=>"Included", "Sjur"=>"Included"} | RAYYAN-INCLUSION: {"Sjur"=>"Excluded"} | RAYYAN-EXCLUSION-REASONS: wrong outcome.

120. Klippel A, Myin-Germeys I, Chavez-Baldini U, et al. Modeling the Interplay Between Psychological Processes and Adverse, Stressful Contexts and Experiences in Pathways to Psychosis: An Experience Sampling Study. *Schizophrenia Bulletin*. 43(2):302-315. RAYYAN-INCLUSION: {"Jone"=>"Included", "Sjur"=>"Included"} | RAYYAN-INCLUSION: {"Sjur"=>"Excluded"} | RAYYAN-LABELS: uknown age of exposure | RAYYAN-EXCLUSION-REASONS: no age.

121. Kmett JA, Eack SM. Characteristics of Sexual Abuse Among Individuals With Serious Mental Illnesses. *Journal of Interpersonal Violence*. 33(17):2725-2744. RAYYAN-INCLUSION: {"Jone"=>"Included", "Sjur"=>"Included"} | RAYYAN-INCLUSION: {"Sjur"=>"Excluded"} | RAYYAN-EXCLUSION-REASONS: wrong outcome.

122. Kotowicz K, Frydecka D, Gaweda L, et al. Effects of traumatic life events, cognitive biases and variation in dopaminergic genes on psychosis proneness. *Early intervention in psychiatry*. 15(2):248-255. RAYYAN-INCLUSION: {"Jone"=>"Included", "Sjur"=>"Included"} | RAYYAN-INCLUSION: {"Sjur"=>"Excluded"} | RAYYAN-LABELS: uknown age of exposure | RAYYAN-EXCLUSION-REASONS: no age.

123. Krkovic K, Schlier B, Lincoln T. An experience sampling study on the nature of the interaction between traumatic experiences, negative affect in everyday life, and threat beliefs. *Schizophrenia Research*. 201:381-387. RAYYAN-INCLUSION: {"Jone"=>"Included", "Sjur"=>"Included"} | RAYYAN-INCLUSION: {"Sjur"=>"Excluded"} | RAYYAN-LABELS: uknown age of exposure | RAYYAN-EXCLUSION-REASONS: wrong outcome.

124. Kroll J, Yusuf AI, Fujiwara K. Psychoses, PTSD, and depression in Somali refugees in Minnesota. *Social Psychiatry and Psychiatric Epidemiology: The International Journal for Research in Social and Genetic Epidemiology and Mental Health Services*. 46(6):481-493. RAYYAN-INCLUSION: {"Jone"=>"Included", "Sjur"=>"Included"} | RAYYAN-INCLUSION: {"Sjur"=>"Excluded"} | RAYYAN-EXCLUSION-REASONS: wrong study design.

125. Lecomte T, Giguere C-E, Cloutier B, Potvin S. Comorbidity profiles of psychotic patients in emergency psychiatry. *Journal of Dual Diagnosis*. 16(2):260-270. RAYYAN-INCLUSION: {"Jone"=>"Included", "Sjur"=>"Included"} | RAYYAN-INCLUSION: {"Sjur"=>"Excluded"} | RAYYAN-EXCLUSION-REASONS: wrong outcome.

126. Liu J, Lim MSM, Ng BT, Chong SA, Subramaniam M, Mahendran R. Global emotion dysregulation and maladaptive cognitive emotion regulation strategies mediate the effects of severe trauma on depressive and positive symptoms in early non-affective psychosis. *Schizophrenia Research*. 222:251-257. RAYYAN-INCLUSION: {"Jone"=>"Included", "Sjur"=>"Included"} | RAYYAN-INCLUSION: {"Sjur"=>"Excluded"} | RAYYAN-LABELS: uknown age of exposure | RAYYAN-EXCLUSION-REASONS: no age.

127. Loch AA, Ara A, Hortencio L, et al. Use of a Bayesian Network Model to predict psychiatric illness in individuals with 'at risk mental states' from a general population cohort. *Neuroscience Letters*. 770:136358. RAYYAN-INCLUSION: {"Jone"=>"Included", "Sjur"=>"Included"} | RAYYAN-INCLUSION: {"Sjur"=>"Excluded"} | RAYYAN-EXCLUSION-REASONS: wrong outcome,wrong study design.

128. Loureiro CM, Corsi-Zuelli F, Fachim HA, et al. Lifetime cannabis use and childhood trauma associated with CNR1 genetic variants increase the risk of psychosis: findings from the STREAM study. *Revista Brasileira de Psiquiatria*. 2023-5 2023;45(3):226-235. doi:doi:https://dx.doi.org/10.47626/1516-4446-2022-2882. RAYYAN-INCLUSION: {"Sjur"=>"Included"} | RAYYAN-INCLUSION: {"Daniel Olsen"=>"Excluded"} | RAYYAN-EXCLUSION-REASONS: duplicate | USER-NOTES: {"Daniel Olsen"=>["Er det duplikat??"]}. RAYYAN-INCLUSION: {"Sjur"=>"Included"} | RAYYAN-INCLUSION: {"Daniel Olsen"=>"Excluded"} | RAYYAN-EXCLUSION-REASONS: duplicate | USER-NOTES: {"Daniel Olsen"=>["Er det duplikat??"]}

129. Lovatt A, Mason O, Brett C, Peters E. Psychotic-Like Experiences, Appraisals, and Trauma. *Journal of Nervous and Mental Disease*. 198(11):813-819. RAYYAN-INCLUSION: {"Jone"=>"Included", "Sjur"=>"Included"} | RAYYAN-INCLUSION: {"Sjur"=>"Excluded"} | RAYYAN-LABELS: uknown age of exposure | RAYYAN-EXCLUSION-REASONS: no age.

130. Lu WL, Yanos PT, Silverstein SM, et al. Public Mental Health Clients with Severe Mental Illness and Probable Posttraumatic Stress Disorder: Trauma Exposure and Correlates of Symptom Severity. *Journal of Traumatic Stress*. 26(2):266-273. RAYYAN-INCLUSION: {"Jone"=>"Included", "Sjur"=>"Included"} | RAYYAN-INCLUSION: {"Sjur"=>"Excluded"} | RAYYAN-EXCLUSION-REASONS: wrong outcome.

131. Løberg E-M, Gjestad R, Posserud M-B, Kompus K, Lundervold AJ. Psychosocial characteristics differentiate non-distressing and distressing voices in 10,346 adolescents. *European Child & Adolescent Psychiatry*. 2019;28(10):1353-1363. RAYYAN-INCLUSION: {"Jone"=>"Included", "Sjur"=>"Included"} | RAYYAN-INCLUSION: {"Sjur"=>"Excluded"} | RAYYAN-EXCLUSION-REASONS: wrong outcome.

132. Malcolm CP, Picchioni MM, Ellett L. Intrusive prospective imagery, posttraumatic intrusions and anxiety in schizophrenia. *Psychiatry Research*. 230(3):899-904. RAYYAN-INCLUSION: {"Jone"=>"Included", "Sjur"=>"Included"} | RAYYAN-INCLUSION: {"Sjur"=>"Excluded"} | RAYYAN-EXCLUSION-REASONS: wrong outcome.

133. Mansueto G, Palmieri S, Faravelli C. Parental style and Cloninger's model in psychosis. *Psychiatry Research*. 269:221-228. RAYYAN-INCLUSION: {"Jone"=>"Included", "Sjur"=>"Included"} | RAYYAN-INCLUSION: {"Sjur"=>"Excluded"} | RAYYAN-LABELS: wrong independent variable | RAYYAN-EXCLUSION-REASONS: wrong predictor | USER-NOTES: {"Sjur"=>["not defined focus on childhood adversity"]}.

134. Mansueto G, van Nierop M, Schruers K, et al. The role of cognitive functioning in the relationship between childhood trauma and a mixed phenotype of affective-anxious-psychotic symptoms in psychotic disorders. *Schizophrenia Research*. 192:262-268. RAYYAN-INCLUSION: {"Jone"=>"Included", "Sjur"=>"Included"} | RAYYAN-INCLUSION: {"Sjur"=>"Excluded"} | RAYYAN-EXCLUSION-REASONS: wrong outcome.

135. Marsh JJ, Narita Z, Zhai FH, Fedina L, Schiffman J, DeVylder J. Violence exposure, psychotic experiences, and social disconnection in an urban community sample. *Psychosis-Psychological Social and Integrative Approaches*. 14(1):57-69. RAYYAN-INCLUSION: {"Jone"=>"Included", "Sjur"=>"Included"} | RAYYAN-INCLUSION: {"Sjur"=>"Excluded"} | RAYYAN-LABELS: wrong independent variable | RAYYAN-EXCLUSION-REASONS: wrong study design.

136. Marwaha S, Broome MR, Bebbington PE, Kuipers E, Freeman D. Mood instability and psychosis: Analyses of British national survey data. *Schizophrenia Bulletin*. 40(2):269-277. RAYYAN-INCLUSION: {"Jone"=>"Included", "Sjur"=>"Included"} | RAYYAN-INCLUSION: {"Sjur"=>"Excluded"} | RAYYAN-LABELS: wrong independent variable | RAYYAN-EXCLUSION-REASONS: wrong predictor.

137. Mazor Y, Gelkopf M, Roe D. Posttraumatic growth among people with serious mental illness, psychosis and posttraumatic stress symptoms. *Comprehensive Psychiatry*. 81:1-9. RAYYAN-INCLUSION: {"Jone"=>"Included", "Sjur"=>"Included"} | RAYYAN-INCLUSION: {"Sjur"=>"Excluded"} | RAYYAN-EXCLUSION-REASONS: wrong outcome.

138. McGrath JJ, Saha S, Lim CCW, et al. Trauma and psychotic experiences: transnational data from the World Mental Health Survey. *British Journal of Psychiatry*. 211(6):373-+. RAYYAN-INCLUSION: {"Jone"=>"Included", "Sjur"=>"Included"} | RAYYAN-INCLUSION: {"Sjur"=>"Excluded"} | RAYYAN-LABELS: exposure above age of 18 | RAYYAN-EXCLUSION-REASONS: no age.

139. Memis CO, Dogan B, Sevincok D, Ashik I, Sevincok L. Mediating role of childhood abuse for the relationship between schizotypal traits and obsessive-compulsive disorder. *Revista de Psiquiatria Clinica*. 47:40-44. RAYYAN-INCLUSION: {"Jone"=>"Included", "Sjur"=>"Included"} | RAYYAN-INCLUSION: {"Sjur"=>"Excluded"} | RAYYAN-EXCLUSION-REASONS: wrong outcome.

140. Mohammadzadeh A, Azadi S, King S, Khosravani V, Sharifi Bastan F. Childhood trauma and the likelihood of increased suicidal risk in schizophrenia. *Psychiatry Research*. 275:100-107. RAYYAN-INCLUSION: {"Jone"=>"Included", "Sjur"=>"Included"} | RAYYAN-INCLUSION: {"Sjur"=>"Excluded"} | RAYYAN-EXCLUSION-REASONS: wrong outcome.

141. Murphy S, McElroy E, Elklit A, Shevlin M, Christoffersen M. Child Maltreatment and Psychiatric Outcomes in Early Adulthood. *Child Abuse Review*. 2020;29(4):365-378. RAYYAN-INCLUSION: {"Jone"=>"Included", "Sjur"=>"Included"} | RAYYAN-INCLUSION: {"Sjur"=>"Excluded"} | RAYYAN-EXCLUSION-REASONS: wrong outcome | USER-NOTES: {"Sjur"=>["to broad outcome of psychosis"]}.

142. Murray R, Mondelli V, Stilo S, et al. The influence of risk factors on the onset and outcome of psychosis: What we learned from the GAP study. *Schizophrenia Research*. 225:63-68. RAYYAN-INCLUSION: {"Jone"=>"Included", "Sjur"=>"Included"} | RAYYAN-INCLUSION: {"Sjur"=>"Excluded"} | RAYYAN-EXCLUSION-REASONS: wrong study design.

143. Mørkved N, Johnsen E, Kroken RA, et al. S2. CHILDHOOD TRAUMA SUBTYPES IN RELATION TO COGNITIVE FUNCTIONING IN SCHIZOPHRENIA SPECTRUM DISORDERS...Congress of the Schizophrenia International Research Society, April 10-14, 2019, Orlando, Florida. *Schizophrenia Bulletin*. 2019 2019;45:S306-S306. doi:doi:10.1093/schbul/sbz020.547. RAYYAN-INCLUSION: {"Sjur"=>"Included"} | RAYYAN-INCLUSION: {"Sjur"=>"Excluded"} | RAYYAN-LABELS: congress abstract. RAYYAN-INCLUSION: {"Sjur"=>"Included"} | RAYYAN-INCLUSION: {"Sjur"=>"Excluded"} | RAYYAN-LABELS: congress abstract

144. Neill C, Read J. Adequacy of Inquiry About, Documentation of, and Treatment of Trauma and Adversities: A Study of Mental Health Professionals in England. *Community Mental Health Journal*. 30:30. RAYYAN-INCLUSION: {"Jone"=>"Included", "Sjur"=>"Included"} | RAYYAN-INCLUSION: {"Sjur"=>"Excluded", "Akiah"=>"Excluded"} | RAYYAN-EXCLUSION-REASONS: wrong outcome.

145. Nestor PG, Hasler VC, O'Donovan K, Lapp HE, Boodai SB, Hunter R. Psychiatric risk and resilience: Plasticity genes and positive mental health. *Brain and Behavior*. 11(6):10. RAYYAN-INCLUSION: {"Jone"=>"Included", "Sjur"=>"Included"} | RAYYAN-INCLUSION: {"Sjur"=>"Excluded", "Akiah"=>"Excluded"} | RAYYAN-EXCLUSION-REASONS: wrong outcome | USER-NOTES: {"Sjur"=>["measure prodromal symptoms only"]}.

146. Newman JM, Turnbull A, Berman BA, Rodrigues S, Serper MR. Impact of Traumatic and Violent Victimization Experiences in Individuals With Schizophrenia and Schizoaffective Disorder. *Journal of Nervous and Mental Disease*. 198(10):708-714. RAYYAN-INCLUSION: {"Jone"=>"Included", "Sjur"=>"Included"} | RAYYAN-INCLUSION: {"Sjur"=>"Excluded", "Akiah"=>"Excluded"} | RAYYAN-LABELS: uknown age of exposure | RAYYAN-EXCLUSION-REASONS: no age.

147. Nicol K, Pope M, Romaniuk L, Hall J. Childhood trauma, midbrain activation and psychotic symptoms in borderline personality disorder. *Transl Psychiatry Psychiatry*. 5:e559. RAYYAN-INCLUSION: {"Jone"=>"Included", "Sjur"=>"Included"} | RAYYAN-INCLUSION: {"Sjur"=>"Excluded", "Akiah"=>"Excluded"} | RAYYAN-EXCLUSION-REASONS: wrong outcome | USER-NOTES: {"Sjur"=>["No test of relationship between CTQ and psychosis"]}.

148. Nischk D, Kügler F, Rockstroh B, Schalinski I. Associations of Childhood Adversities with Severity of Illness and Course of Remission from Acute Psychosis. *Zeitschrift fur Klinische Psychologie und Psychotherapie*. 2019;48(4):193-203. RAYYAN-INCLUSION: {"Jone"=>"Included", "Sjur"=>"Included"} | RAYYAN-INCLUSION: {"Sjur"=>"Excluded", "Akiah"=>"Excluded"} | RAYYAN-EXCLUSION-REASONS: foreign language | USER-NOTES: {"Akiah"=>["Fant aldri fulltekst men inkluderer på basis av abstract"]}.

149. O'Hare T, Shen C, Sherrer M. Differences in trauma and posttraumatic stress symptoms in clients with schizophrenia spectrum and major mood disorders. *Psychiatry Research*. 205(1):85-89. RAYYAN-INCLUSION: {"Jone"=>"Included", "Sjur"=>"Included"} | RAYYAN-INCLUSION: {"Sjur"=>"Excluded", "Akiah"=>"Excluded"} | RAYYAN-LABELS: uknown age of exposure | RAYYAN-EXCLUSION-REASONS: no age.

150. O'Hare T, Shen C, Sherrer MV. Lifetime Abuse and Self-Harm in People With Severe Mental Illness: A Structural Equation Model. *Psychological Trauma-Theory Research Practice and Policy*. 7(4):348-355. RAYYAN-INCLUSION: {"Jone"=>"Included", "Sjur"=>"Included"} | RAYYAN-INCLUSION: {"Sjur"=>"Excluded", "Akiah"=>"Excluded"} | RAYYAN-LABELS: uknown age of exposure | RAYYAN-EXCLUSION-REASONS: no age.

151. Okkels N, Trabjerg B, Arendt M, Pedersen CB. Traumatic Stress Disorders and Risk of Subsequent Schizophrenia Spectrum Disorder or Bipolar Disorder: A Nationwide Cohort Study. *Schizophrenia Bulletin*. 43(1):180-186. RAYYAN-INCLUSION: {"Jone"=>"Included", "Sjur"=>"Included"} | RAYYAN-INCLUSION: {"Sjur"=>"Excluded", "Akiah"=>"Excluded"} | RAYYAN-LABELS: wrong independent variable | RAYYAN-EXCLUSION-REASONS: wrong predictor.

152. Paino M, Ordonez-Camblor N, Fonseca-Pedrero E, Garcia-Alvarez L, Pizarro-Ruiz JP. Development and Validation of an Instrument for the Detection of Early Traumatic Experiences (ExpTra-S) in Patients With Psychosis. *Frontiers in Psychology*. 11:9. RAYYAN-INCLUSION: {"Sjur"=>"Included", "Akiah"=>"Included"} | RAYYAN-INCLUSION: {"Sjur"=>"Excluded", "Akiah"=>"Excluded"} | RAYYAN-EXCLUSION-REASONS: wrong study design.

153. Pazar O, Steele H, Todman M. Expressed emotion and reflective functioning across the schizotypy spectrum: Developmental experiential factors. *Psychoanalytic Psychology*.No Pagination Specified. RAYYAN-INCLUSION: {"Sjur"=>"Included", "Akiah"=>"Included"} | RAYYAN-INCLUSION: {"Sjur"=>"Excluded", "Akiah"=>"Excluded"} | RAYYAN-LABELS: wrong independent variable | RAYYAN-EXCLUSION-REASONS: wrong predictor | USER-NOTES: {"Sjur"=>["used CT as a selection of sample"]}.

154. Peralta V, Garcia de Jalon E, Moreno-Izco L, et al. Long-Term Outcomes of First-Admission Psychosis: A Naturalistic 21-Year Follow-Up Study of Symptomatic, Functional and Personal Recovery and Their Baseline Predictors. *Schizophrenia Bulletin*. 6:06. RAYYAN-INCLUSION: {"Sjur"=>"Included", "Akiah"=>"Included"} | RAYYAN-INCLUSION: {"Sjur"=>"Excluded", "Akiah"=>"Excluded"} | RAYYAN-LABELS: no instruement,wrong independent variable | RAYYAN-EXCLUSION-REASONS: wrong predictor,wrong population.

155. Picken A, Tarrier N. Trauma and comorbid posttraumatic stress disorder in individuals with schizophrenia and substance abuse. *Comprehensive Psychiatry*. 52(5):490-497. RAYYAN-INCLUSION: {"Sjur"=>"Included", "Akiah"=>"Included"} | RAYYAN-LABELS: Unsure age range of instrument | RAYYAN-INCLUSION: {"Sjur"=>"Excluded", "Akiah"=>"Excluded"} | RAYYAN-LABELS: uknown age of exposure.

156. Rajan G, Ljunggren G, ell P, Wahlstrom L, Svedin C-G, Carlsson AC. Diagnoses of sexual abuse and their common registered comorbidities in the total population of Stockholm. *Journal of Epidemiology and Community Health*. 71(6):592-598. RAYYAN-INCLUSION: {"Sjur"=>"Included", "Akiah"=>"Included"} | RAYYAN-INCLUSION: {"Sjur"=>"Excluded", "Akiah"=>"Excluded"} | RAYYAN-EXCLUSION-REASONS: no age | USER-NOTES: {"Akiah"=>["Det er registrert kontakt med lege om seksuelle overgrep men ikke nødvendigvis i barndommen. Tenker du at den er med fordi de også inkluderer barn? "]}.

157. Rajkumar AP, Chitra C, Bhuvaneshwari S, Poonkuzhali B, Kuruvilla A, Jacob KS. Clinical Predictors of Response to Clozapine in Patients with Treatment Resistant Schizophrenia. *Psychopharmacology Bulletin*. 44(3):51-65. RAYYAN-INCLUSION: {"Sjur"=>"Included", "Wenche ten Velden"=>"Included"} | RAYYAN-INCLUSION: {"Sjur"=>"Excluded", "Wenche ten Velden"=>"Excluded"} | RAYYAN-EXCLUSION-REASONS: wrong outcome.

158. Reiff M, Castille DM, Muenzenmaier K, Link B. Childhood abuse and the content of adult psychotic symptoms. *Psychological Trauma: Theory, Research, Practice, and Policy*. 4(4):356-369. RAYYAN-INCLUSION: {"Sjur"=>"Included", "Akiah"=>"Included"} | USER-NOTES: {"Akiah"=>["Jeg tenker fortsatt at denne kanskje bør leses mer nøye. De kartlegger barndomstraume hos mennesker med alvorlig psykisk lidelse og utfall er spesifikke psykose symptomer. Men si i fra hvis det e rnoe jeg ikke har forstått med denne. \n\nAkiah "]} | RAYYAN-INCLUSION: {"Sjur"=>"Excluded", "Akiah"=>"Excluded"} | RAYYAN-EXCLUSION-REASONS: wrong study design.

159. Rokita KI, Dauvermann MR, Mothersill D, et al. Childhood trauma, parental bonding, and social cognition in patients with schizophrenia and healthy adults. *Journal of Clinical Psychology*. 77(1):241-253. RAYYAN-INCLUSION: {"Sjur"=>"Included", "Akiah"=>"Included"} | RAYYAN-INCLUSION: {"Sjur"=>"Excluded", "Akiah"=>"Excluded"} | RAYYAN-EXCLUSION-REASONS: wrong outcome | USER-NOTES: {"Akiah"=>["Er ikke utfall schizofreni? "]}.

160. Rosen C, Jones N, Longden E, et al. Exploring the Intersections of Trauma, Structural Adversity, and Psychosis among a Primarily African-American Sample: A Mixed-Methods Analysis. *Frontiers in Psychiatry*. 8:11. RAYYAN-INCLUSION: {"Sjur"=>"Included", "Akiah"=>"Included"} | RAYYAN-INCLUSION: {"Sjur"=>"Excluded", "Akiah"=>"Excluded"} | RAYYAN-LABELS: uknown age of exposure.

161. Russo DA, Stochl J, Hodgekins J, et al. Attachment styles and clinical correlates in people at ultra high risk for psychosis. *British Journal of Psychology*. 109(1):45-62. RAYYAN-INCLUSION: {"Sjur"=>"Included", "Akiah"=>"Included"} | RAYYAN-INCLUSION: {"Sjur"=>"Excluded", "Akiah"=>"Excluded"} | RAYYAN-EXCLUSION-REASONS: wrong outcome | USER-NOTES: {"Akiah"=>["Instrumentet spør om alder når trauma inntraff men jeg kan ikke se at det brukes i analysene - altså at det spesifiseres at det var barndomstraumer. "]}.

162. Samplin E, Ikuta T, Malhotra AK, Szeszko PR, DeRosse P. Sex differences in resilience to childhood maltreatment: Effects of trauma history on hippocampal volume, general cognition and subclinical psychosis in healthy adults. *Journal of Psychiatric Research*. 47(9):1174-1179. RAYYAN-INCLUSION: {"Sjur"=>"Included", "Akiah"=>"Included"} | RAYYAN-INCLUSION: {"Sjur"=>"Excluded", "Akiah"=>"Excluded"} | RAYYAN-EXCLUSION-REASONS: wrong outcome | USER-NOTES: {"Akiah"=>["De skriver subclinical psychosis i tittelen men alt jeg finner i resultater er at emotional abuse har sammenheng med positive, negative and depressive symtpoms. Jeg regner med at de mener positive psykose symptomer og derfor nevner det i tittelen - men jeg synes ikke at de begrunner dette godt i teksten eller i funnene for den saks skyld. \nAltså de kartlegger barndomstraume og blant annet det de kaller for positive symptomer (av hva) hos friske voksen. Men om det er uttrykk for psykosenære symptomer det er jeg usikker på altså. "]}.

163. Saxena A, Dodell-Feder D. Explaining the Association Between Urbanicity and Psychotic-Like Experiences in Pre-Adolescence: The Indirect Effect of Urban Exposures. *Frontiers in Psychiatry*. 2022-3 2022;13:11. doi:doi:10.3389/fpsyt.2022.831089. RAYYAN-INCLUSION: {"Sjur"=>"Included"} | RAYYAN-INCLUSION: {"Sjur"=>"Excluded"} | RAYYAN-LABELS: no childhood adversity. RAYYAN-INCLUSION: {"Sjur"=>"Included"} | RAYYAN-INCLUSION: {"Sjur"=>"Excluded"} | RAYYAN-LABELS: no childhood adversity

164. Schafer I, Eiroa-Orosa FJ, Schroeder K, Harfst T, Aderhold V. Post-traumatic Disorders in Patients with schizophrenia Spectrum Disorders. *Nervenarzt*. 86(7):818-825. RAYYAN-INCLUSION: {"Sjur"=>"Included", "Akiah"=>"Included"} | RAYYAN-INCLUSION: {"Sjur"=>"Excluded", "Akiah"=>"Excluded"} | USER-NOTES: {"Akiah"=>["På tysk"]}.

165. Schafer I, Fisher HL, Aderhold V, et al. Dissociative symptoms in patients with schizophrenia: Relationships with childhood trauma and psychotic symptoms. *Comprehensive Psychiatry*. 53(4):364-371. RAYYAN-INCLUSION: {"Sjur"=>"Included", "Akiah"=>"Included"} | RAYYAN-INCLUSION: {"Sjur"=>"Excluded", "Akiah"=>"Excluded"} | RAYYAN-EXCLUSION-REASONS: wrong outcome.

166. Schlesselmann AJ, Huntjens RJC, Renard SB, et al. A Network Approach to Trauma, Dissociative Symptoms, and Psychosis Symptoms in Schizophrenia Spectrum Disorders. *Schizophrenia Bulletin*. 2022-9 2022:10. doi:doi:10.1093/schbul/sbac122. RAYYAN-INCLUSION: {"Sjur"=>"Included"} | RAYYAN-INCLUSION: {"Sjur"=>"Excluded"} | RAYYAN-LABELS: Unknown age. RAYYAN-INCLUSION: {"Sjur"=>"Included"} | RAYYAN-INCLUSION: {"Sjur"=>"Excluded"} | RAYYAN-LABELS: Unknown age

167. Schroeder K, Ehlers S, Schafer I. Differences in the phenomenology of verbal acoustic hallucinations in patients with schizophrenia-spectrum disorders with and without exposure to violence in childhood. *Trauma & Gewalt*. 11(3):196-205. RAYYAN-INCLUSION: {"Sjur"=>"Included", "Akiah"=>"Included"} | RAYYAN-INCLUSION: {"Sjur"=>"Excluded", "Akiah"=>"Excluded"} | RAYYAN-EXCLUSION-REASONS: foreign language.

168. Schroeder K, Langel, Willemien, Fisher HL, Huber CG, Schafer I. Dissociation in patients with schizophrenia spectrum disorders: What is the role of different types of childhood adversity? *Comprehensive Psychiatry*. 68:201-208. RAYYAN-INCLUSION: {"Sjur"=>"Included", "Akiah"=>"Included"} | RAYYAN-INCLUSION: {"Sjur"=>"Excluded", "Akiah"=>"Excluded"} | RAYYAN-EXCLUSION-REASONS: wrong outcome.

169. Sener A, Tomruk NB, Evren C, Karaytug MO. The relation of childhood trauma with dissociation in patients with schizophrenia. *Cukurova Medical Journal*. 2020;45(3):1024-1032. RAYYAN-INCLUSION: {"Sjur"=>"Included", "Akiah"=>"Included"} | RAYYAN-INCLUSION: {"Sjur"=>"Excluded", "Akiah"=>"Excluded"} | RAYYAN-EXCLUSION-REASONS: foreign language.

170. Shalev A, Merranko J, Gill MK, et al. Longitudinal course and risk factors associated with psychosis in bipolar youths. *Bipolar Disorders*. 22(2):139-154. RAYYAN-INCLUSION: {"Sjur"=>"Included", "Akiah"=>"Included"} | RAYYAN-INCLUSION: {"Sjur"=>"Excluded", "Akiah"=>"Excluded"} | RAYYAN-LABELS: wrong independent variable | RAYYAN-EXCLUSION-REASONS: wrong predictor | USER-NOTES: {"Akiah"=>["Jeg tror at physical/sexual abuse er registrert gjennom intervju med Kiddie-Sads uten at jeg fant det ut helt sikkert ved å lese gjennom metoden. "]}.

171. Sheinbaum T, Bifulco A, Ballespi S, Mitjavila M, Kwapil TR, Barrantes-Vidal N. Interview investigation of insecure attachment styles as mediators between poor childhood care and schizophrenia- spectrum phenomenology. *PLoS ONE Vol 10(8), 2015, ArtID e0135150*. 10(8)RAYYAN-INCLUSION: {"Sjur"=>"Included", "Akiah"=>"Included"} | RAYYAN-INCLUSION: {"Sjur"=>"Excluded", "Akiah"=>"Excluded"} | RAYYAN-EXCLUSION-REASONS: wrong study design.

172. Smeets F, Lataster T, Viechtbauer W, Delespaul P. Evidence that environmental and genetic risks for psychotic disorder may operate by impacting on connections between core symptoms of perceptual alteration and delusional ideation. *Schizophrenia Bulletin*. 41(3):687-697. RAYYAN-INCLUSION: {"Sjur"=>"Included", "Akiah"=>"Included"} | RAYYAN-INCLUSION: {"Sjur"=>"Excluded", "Akiah"=>"Excluded"} | RAYYAN-LABELS: wrong independent variable | RAYYAN-EXCLUSION-REASONS: wrong predictor | USER-NOTES: {"Akiah"=>["De har med CTQ som en kontroll/confounding variabel - skal den ikke med da? "]}.

173. Someshwar A, Holla B, Pansari Agarwal P, et al. Adverse childhood experiences in families with multiple members diagnosed to have psychiatric illnesses. *Australian and New Zealand Journal of Psychiatry*. 54(11):1086-1094. RAYYAN-INCLUSION: {"Sjur"=>"Included", "Akiah"=>"Included"} | RAYYAN-INCLUSION: {"Sjur"=>"Excluded", "Akiah"=>"Excluded"} | RAYYAN-EXCLUSION-REASONS: wrong outcome | USER-NOTES: {"Akiah"=>["52 hadde schizofreni? "]}.

174. Strauss GP, Raugh IM, Mittal VA, Gibb B, E o, Coles ME. Bullying victimization and perpetration in a community sample of youth with psychotic like experiences. *Schizophrenia Research*. 195:534-536. RAYYAN-INCLUSION: {"Sjur"=>"Included", "Akiah"=>"Included"} | RAYYAN-INCLUSION: {"Sjur"=>"Excluded", "Akiah"=>"Excluded"} | RAYYAN-EXCLUSION-REASONS: wrong publication type | USER-NOTES: {"Akiah"=>["Letter to the editor"]}.

175. Sweeney S, Air T, Zannettino L, Galletly C. Gender Differences in the Physical and Psychological Manifestation of Childhood Trauma and/or Adversity in People with Psychosis. *Frontiers in Psychology*. 2015 2015;6:1768. doi:doi:https://dx.doi.org/10.3389/fpsyg.2015.01768. RAYYAN-INCLUSION: {"Sjur"=>"Included"} | RAYYAN-INCLUSION: {"Sjur"=>"Excluded"} | RAYYAN-EXCLUSION-REASONS: wrong outcome. RAYYAN-INCLUSION: {"Sjur"=>"Included"} | RAYYAN-INCLUSION: {"Sjur"=>"Excluded"} | RAYYAN-EXCLUSION-REASONS: wrong outcome

176. Tessner KD, Mittal V, Walker EF. Longitudinal study of stressful life events and daily stressors among adolescents at high risk for psychotic disorders. *Schizophrenia Bulletin*. 37(2):432-441. RAYYAN-INCLUSION: {"Sjur"=>"Included", "Akiah"=>"Included"} | RAYYAN-INCLUSION: {"Sjur"=>"Excluded", "Akiah"=>"Excluded"} | RAYYAN-LABELS: wrong independent variable | RAYYAN-EXCLUSION-REASONS: wrong predictor.

177. Thonney J, Conus P, Golay P. Sexual and physical abuse during childhood; what is the impact on outcome in first episode psychosis patients? *L'Encephale: Revue de psychiatrie clinique biologique et therapeutique*. 47(3):215-220. RAYYAN-INCLUSION: {"Sjur"=>"Included", "Akiah"=>"Included"} | RAYYAN-INCLUSION: {"Sjur"=>"Excluded", "Akiah"=>"Excluded"} | RAYYAN-EXCLUSION-REASONS: foreign language.

178. Tognin S, Catalan A, Modinos G, et al. Emotion recognition and adverse childhood experiences in individuals at clinical high risk of psychosis. *Schizophrenia Bulletin*. 46(4):823-833. RAYYAN-INCLUSION: {"Sjur"=>"Included", "Akiah"=>"Included"} | RAYYAN-INCLUSION: {"Sjur"=>"Excluded", "Akiah"=>"Excluded"} | RAYYAN-EXCLUSION-REASONS: wrong outcome.

179. Tolmeijer E, Hardy A, Jongeneel A, Staring ABP, van der Gaag M, Berg DVD. Voice-hearers' beliefs about the causes of their voices. *Psychiatry Research*. 302RAYYAN-INCLUSION: {"Sjur"=>"Included", "Akiah"=>"Included"} | RAYYAN-INCLUSION: {"Sjur"=>"Excluded", "Akiah"=>"Excluded"} | RAYYAN-LABELS: uknown age of exposure,wrong independent variable | RAYYAN-EXCLUSION-REASONS: no age.

180. Tomassi S, Tosato S. Epigenetics and gene expression profile in first-episode psychosis: The role of childhood trauma. *Neuroscience and Biobehavioral Reviews*. 83:226-237. RAYYAN-INCLUSION: {"Sjur"=>"Included", "Akiah"=>"Included"} | RAYYAN-INCLUSION: {"Sjur"=>"Excluded", "Akiah"=>"Excluded"} | RAYYAN-EXCLUSION-REASONS: wrong study design.

181. Trotta A, Arseneault L, Danese A, Mondelli V, Rasmussen LJH, Fisher HL. Associations between childhood victimization, inflammatory biomarkers and psychotic phenomena in adolescence: A longitudinal cohort study. *Brain, Behavior, & Immunity*. 2021-11-11 2021;98:74-85. doi:doi:https://dx.doi.org/10.1016/j.bbi.2021.08.209. RAYYAN-INCLUSION: {"Sjur"=>"Included"} | RAYYAN-INCLUSION: {"Sjur"=>"Excluded"} | RAYYAN-EXCLUSION-REASONS: wrong outcome. RAYYAN-INCLUSION: {"Sjur"=>"Included"} | RAYYAN-INCLUSION: {"Sjur"=>"Excluded"} | RAYYAN-EXCLUSION-REASONS: wrong outcome

182. Vaskinn A, Engelstad KN, Torgalsboen A-K, Rund BR. Childhood trauma, social cognition and schizophrenia: Specific association between physical neglect and cognitive theory of mind in homicide offenders. *Psychiatry Research Vol 303 2021, ArtID 114093*. 303RAYYAN-INCLUSION: {"Sjur"=>"Included", "Akiah"=>"Included"} | RAYYAN-INCLUSION: {"Sjur"=>"Excluded", "Akiah"=>"Excluded"} | RAYYAN-EXCLUSION-REASONS: wrong outcome.

183. Vaskinn A, Melle I, Aas M, Berg AO. Sexual abuse and physical neglect in childhood are associated with affective theory of mind in adults with schizophrenia. *Schizophrenia Research*. 23:100189. RAYYAN-INCLUSION: {"Sjur"=>"Included", "Akiah"=>"Included"} | RAYYAN-INCLUSION: {"Sjur"=>"Excluded", "Akiah"=>"Excluded"} | RAYYAN-EXCLUSION-REASONS: wrong outcome | USER-NOTES: {"Sjur"=>["TOM som utfall?"]}.

184. Velikonja T, Velthorst E, McClure M, et al. Severe childhood trauma and clinical and neurocognitive features in schizotypal personality disorder. *Acta Psychiatrica Scandinavica*. 140(1):50-64. RAYYAN-INCLUSION: {"Sjur"=>"Included", "Akiah"=>"Included"} | RAYYAN-INCLUSION: {"Sjur"=>"Excluded", "Akiah"=>"Excluded"} | RAYYAN-EXCLUSION-REASONS: wrong outcome | USER-NOTES: {"Sjur"=>["TOM som utfall?"]}.

185. Wang D-S, Chung C-H, Chang H-A, et al. Association between child abuse exposure and the risk of psychiatric disorders: A nationwide cohort study in Taiwan. *Child Abuse & Neglect Vol 101 2020, ArtID 104362*. 101RAYYAN-INCLUSION: {"Sjur"=>"Included", "Akiah"=>"Included"} | RAYYAN-INCLUSION: {"Sjur"=>"Excluded", "Akiah"=>"Excluded"} | RAYYAN-EXCLUSION-REASONS: wrong outcome | USER-NOTES: {"Sjur"=>["For uspesifikk avhengig variabel? psykiatrisk lidelse"]}.

186. Wearne D, Curtis GJ, Genetti A, Samuel M, Sebastian J. Where pseudo-hallucinations meet dissociation: A cluster analysis. *Australasian Psychiatry*. 25(4):364-368. RAYYAN-INCLUSION: {"Sjur"=>"Included", "Akiah"=>"Included"} | RAYYAN-INCLUSION: {"Sjur"=>"Excluded", "Akiah"=>"Excluded"} | RAYYAN-EXCLUSION-REASONS: wrong outcome | USER-NOTES: {"Akiah"=>["Det står ikke hvordan de har målt det så jeg tenker denne skal eksluderes - men skriver et notat dersom du er uenig. "]}.

187. Wearne D, Curtis GJ, Melvill-Smith P, et al. Exploring the relationship between auditory hallucinations, trauma and dissociation. *BJPsych Open Vol 6 2020, ArtID e54*. 6RAYYAN-INCLUSION: {"Sjur"=>"Included", "Akiah"=>"Included"} | RAYYAN-INCLUSION: {"Sjur"=>"Excluded", "Akiah"=>"Excluded"} | RAYYAN-LABELS: wrong independent variable | RAYYAN-EXCLUSION-REASONS: wrong outcome.

188. Xiang Z, Liu Z, Cao H, Wu Z, Long Y. Evaluation on Long-Term Test-Retest Reliability of the Short-Form Childhood Trauma Questionnaire in Patients with Schizophrenia. *Psychology Research & Behavior Management*. 2021;14:1033-1040. RAYYAN-INCLUSION: {"Sjur"=>"Included", "Akiah"=>"Included"} | RAYYAN-INCLUSION: {"Sjur"=>"Excluded", "Akiah"=>"Excluded"} | RAYYAN-EXCLUSION-REASONS: wrong study design | USER-NOTES: {"Sjur"=>["Validering av et instrument er ikke design vi tar med"]}.

189. Xie P, Wu K, Zheng Y, et al. Prevalence of childhood trauma and correlations between childhood trauma, suicidal ideation, and social support in patients with depression, bipolar disorder, and schizophrenia in southern China. *Journal of Affective Disorders*. 228:41-48. RAYYAN-INCLUSION: {"Sjur"=>"Included", "Akiah"=>"Included"} | RAYYAN-INCLUSION: {"Sjur"=>"Excluded", "Akiah"=>"Excluded"} | RAYYAN-EXCLUSION-REASONS: wrong outcome | USER-NOTES: {"Sjur"=>["For uspesifikt? Ser på pasientgruppen samlet sett og ikke på psykose spesifikt?"]}.

190. Yung A, Cotter J, Wood S, et al. Childhood maltreatment and transition to psychotic disorder independently predict long-term functioning in young people at ultra-high risk for psychosis. *Psychological Medicine*. 45(16):3453-3465. RAYYAN-INCLUSION: {"Sjur"=>"Included", "Akiah"=>"Included"} | RAYYAN-INCLUSION: {"Sjur"=>"Excluded", "Akiah"=>"Excluded"} | RAYYAN-EXCLUSION-REASONS: wrong outcome | USER-NOTES: {"Sjur"=>["fungering som DV"]}.

191. Zincir SB, Yanartas O, Zincir S, Semiz UB. Clinical correlates of childhood trauma and dissociative phenomena in patients with severe psychiatric disorders. *Psychiatric Quarterly*. 85(4):417-426. RAYYAN-INCLUSION: {"Sjur"=>"Included", "Akiah"=>"Included"} | RAYYAN-INCLUSION: {"Sjur"=>"Excluded", "Akiah"=>"Excluded"} | RAYYAN-EXCLUSION-REASONS: wrong outcome | USER-NOTES: {"Sjur"=>["Kvalifiseres ikke som prevalens studie og feil utfall (DES)"]}.

192. Zouraraki C, Kyriklaki A, Economou E, Giakoumaki SG. The moderating role of early traumatic experiences on the association of schizotypal traits with visual perception. *Scandinavian Journal of Psychology*. 2023-2 2023;64(1):10-20. doi:doi:https://dx.doi.org/10.1111/sjop.12859. RAYYAN-INCLUSION: {"Sjur"=>"Included"} | RAYYAN-INCLUSION: {"Sjur"=>"Excluded"} | RAYYAN-EXCLUSION-REASONS: wrong outcome. RAYYAN-INCLUSION: {"Sjur"=>"Included"} | RAYYAN-INCLUSION: {"Sjur"=>"Excluded"} | RAYYAN-EXCLUSION-REASONS: wrong outcome

193. Aakre JM, Brown CH, Benson KM, Drapalski AL, Gearon JS. Trauma exposure and PTSD in women with schizophrenia and coexisting substance use disorders: Comparisons to women with severe depression and substance use disorders. *Psychiatry Research*. 220(3):840-845. RAYYAN-INCLUSION: {"Sjur"=>"Included", "Akiah"=>"Included"} | RAYYAN-INCLUSION: {"Sjur"=>"Excluded", "Akiah"=>"Excluded"} | RAYYAN-LABELS: uknown age of exposure | RAYYAN-EXCLUSION-REASONS: no age | USER-NOTES: {"Sjur"=>["Traumatic Life Events Questionnaire (TLEQ, Kubany et al., 2000). This instrument \ncovers a range of commonly-assessed childhood and adulthood traumatic events. Evidence \nindicates that the reliability over time of self-reports of trauma in childhood and adulthood \nby a subsample of the current sample of women with SUDs and schizophrenia is quite \nsatisfactory"]}.

194. Aas M, Dazzan P, Fisher HL, et al. Childhood trauma and cognitive function in first-episode affective and non-affective psychosis. *Schizophrenia Research*. 129(1):12-19. RAYYAN-INCLUSION: {"Sjur"=>"Included", "Akiah"=>"Included"} | RAYYAN-INCLUSION: {"Sjur"=>"Excluded", "Akiah"=>"Excluded"} | RAYYAN-EXCLUSION-REASONS: wrong outcome.

195. Aas M, Dieset I, Hope S, et al. Childhood maltreatment severity is associated with elevated C-reactive protein and body mass index in adults with schizophrenia and bipolar diagnoses. *Brain, Behavior, and Immunity*. 65:342-349. RAYYAN-INCLUSION: {"Sjur"=>"Included", "Akiah"=>"Included"} | RAYYAN-INCLUSION: {"Sjur"=>"Excluded", "Akiah"=>"Excluded"} | RAYYAN-EXCLUSION-REASONS: wrong outcome.

196. Aas M, Djurovic S, Athanasiu L, et al. Serotonin transporter gene polymorphism, childhood trauma, and cognition in patients with psychotic disorders. *Schizophrenia Bulletin*. 38(1):15-22. RAYYAN-INCLUSION: {"Sjur"=>"Included", "Akiah"=>"Included"} | RAYYAN-INCLUSION: {"Sjur"=>"Excluded", "Akiah"=>"Excluded"} | RAYYAN-EXCLUSION-REASONS: wrong outcome.

197. Aas M, Elvsåshagen T, Westlye LT, et al. O1.6. TELOMERE LENGTH IS ASSOCIATED WITH CHILDHOOD TRAUMA IN PATIENTS WITH SEVERE MENTAL DISORDERS...Congress of the Schizophrenia International Research Society, April 10-14, 2019, Orlando, Florida. *Schizophrenia Bulletin*. 2019 2019;45:S160-S161. doi:doi:10.1093/schbul/sbz021.182. RAYYAN-INCLUSION: {"Sjur"=>"Included"} | RAYYAN-INCLUSION: {"Sjur"=>"Excluded"} | RAYYAN-LABELS: congress abstract. RAYYAN-INCLUSION: {"Sjur"=>"Included"} | RAYYAN-INCLUSION: {"Sjur"=>"Excluded"} | RAYYAN-LABELS: congress abstract

198. Aas M, Haukvik UK, Djurovic S, et al. BDNF val66met modulates the association between childhood trauma, cognitive and brain abnormalities in psychoses. *Progress in Neuro-Psychopharmacology & Biological Psychiatry*. 46:181-188. RAYYAN-INCLUSION: {"Sjur"=>"Included", "Akiah"=>"Included"} | RAYYAN-INCLUSION: {"Sjur"=>"Excluded", "Akiah"=>"Excluded"} | RAYYAN-EXCLUSION-REASONS: wrong outcome.

199. Aas M, Haukvik UK, Djurovic S, et al. Interplay between childhood trauma and BDNF val66met variants on blood BDNF mRNA levels and on hippocampus subfields volumes in schizophrenia spectrum and bipolar disorders. *Journal of Psychiatric Research*. 59:14-21. RAYYAN-INCLUSION: {"Sjur"=>"Included", "Akiah"=>"Included"} | RAYYAN-INCLUSION: {"Sjur"=>"Excluded", "Akiah"=>"Excluded"} | RAYYAN-EXCLUSION-REASONS: wrong outcome.

200. Aas M, Kauppi K, Br, et al. Childhood trauma is associated with increased brain responses to emotionally negative as compared with positive faces in patients with psychotic disorders. *Psychological Medicine*. 47(4):669-679. RAYYAN-INCLUSION: {"Sjur"=>"Included", "Akiah"=>"Included"} | RAYYAN-INCLUSION: {"Sjur"=>"Excluded", "Akiah"=>"Excluded"} | RAYYAN-EXCLUSION-REASONS: wrong outcome.

201. Aas M, Navari S, Gibbs A, et al. Is there a link between childhood trauma, cognition, and amygdala and hippocampus volume in first-episode psychosis? *Schizophrenia Research*. 137(1):73-79. RAYYAN-INCLUSION: {"Sjur"=>"Included", "Akiah"=>"Included"} | RAYYAN-INCLUSION: {"Sjur"=>"Excluded", "Akiah"=>"Excluded"} | RAYYAN-EXCLUSION-REASONS: wrong outcome.

202. Aas M, Uel, T., et al. Childhood Trauma Is Nominally Associated With Elevated Cortisol Metabolism in Severe Mental Disorder. *Frontiers in psychiatry Frontiers Research Foundation*. 2020;11:391. RAYYAN-INCLUSION: {"Sjur"=>"Included", "Akiah"=>"Included"} | RAYYAN-INCLUSION: {"Sjur"=>"Excluded", "Akiah"=>"Excluded"} | RAYYAN-EXCLUSION-REASONS: wrong outcome.

203. Aas M, Uel, Torill, et al. Physical activity and childhood trauma experiences in patients with schizophrenia or bipolar disorders. *The World Journal of Biological Psychiatry*. 22(8):637-645. RAYYAN-INCLUSION: {"Sjur"=>"Included", "Akiah"=>"Included"} | RAYYAN-INCLUSION: {"Sjur"=>"Excluded", "Akiah"=>"Excluded"} | RAYYAN-EXCLUSION-REASONS: wrong outcome.
